# Advances in Research at Synthesis Process Optimization and Quality Standard Improvement of *O*-desmethylvenlafaxine Succinate

**DOI:** 10.3389/fchem.2022.860292

**Published:** 2022-08-17

**Authors:** Shiwei Yang, Shiyun Chen, Cheng Wang, Shibo Zhang, Shuaifei Li, Xinsong Yuan, Fuyun Peng, Yong He

**Affiliations:** ^1^ Department of Chemistry and Chemical Engineering, Hefei Normal University, Hefei, China; ^2^ Department of Chemistry and Chemical Engineering, Hefei University of Technology, Hefei, China; ^3^ Department of Energy Materials and Chemical Engineering, Hefei University, Hefei, China

**Keywords:** O-desmethylvenlafaxine, genotoxic impurities, XRD powder diffraction, 1, 2-nucleophilic addition, antidepressants

## Abstract

We herein describe an optimal approach for the efficient synthesis of *O*-desmethylvenlafaxine succinate monohydrate (DVS) with high yield and high purity through 5-step reactions, including benzyl protection of the phenolic hydroxyl group, cyclohexanone condensation, deprotection, cyano reduction, dimethylation, and succinic acid salt formation from *p*-hydroxybenzene acetonitrile as a starting material. 4-Benzyloxyphenylacetonitrile (Intermediate I) was prepared by the hydroxyl protection of the bromide benzyl-*p*-hydroxyphenylacetonitrile catalyzed by potassium carbonate with 99.83% purity and 98.92% yields. The 1, 2-nucleophilic addition of intermediate I to cyclohexanone promoted by sodium hydroxide with the homogeneous catalyst (n-Bu)_4_N^+^Br^−^ to the preparation of 1-[Cyano(4-benzyloxyphenyl)methyl]cyclohexanol (Intermediate II) was obtained by 99.13% purity and 99.71% yields. Cyclohexanone residues and benzyl bromide residues were trace, and tetrabutylammonium bromide residues were UNDER 0.7 ppm, which further improves the residual standards for genotoxic impurities (GIs). 1-[2-amino-1-(4-hydroxyphenyl)ethyl]cyclohexanol hydrochloride (Intermediate III) was prepared by 10% palladium-carbon under 2.0 MPa up to 98.32% purity and 94.20% yields. *O*-desmethylvenlafaxine (ODV) was synthesized by dimethylation of intermediate III with 37% formaldehyde solution and 85% formic acid solution. The highest purity was up to 99.20% and the yield was up to 84.77%. *O*-desmethylvenlafaxine succinate monohydrate (DVS) was formed from succinic acid and *O*-desmethylvenlafaxine (ODV) and crystallized in a mixed solvent of acetone and water (3:1) to obtain 99.92% purity and 90.27% yields. The 5-step total yields of desvenlafaxine succinate monohydrate is 71.09%, and its crystal form has characteristic peaks at 5, 10, 21, and 26 min by XRD powder diffraction, which is consistent with the crystalline form I. Compared with conventional synthesis strategy, we revealed a novel and green process with a high total yield, high atomic economy, low environmental pollution, high operational safety, and high residual standards for genotoxic impurities (GIs), which improves drug safety.

## Introduction

*O*-desmethylvenlafaxine (Deecher et al., 2006; Sopko et al., 2008; Venu et al., 2008; Liu et al., 2016; Da Silva et al., 2020; Gahr et al., 2020) is the major active metabolite of the third serotonin-norepinephrine reuptake inhibitor (SNRI) venlafaxine approved by the US Food and Drug Administration for major depressive disorder (MDD), used to treat mild or severe depression, anxiety, and other mental disorders with superior clinical advantages (Klamerus et al., 1992; Safer and Zito, 2019; Zhang et al., 2019; Agüera-Ortiz et al., 2020). The efficacy, safety, or tolerability of preparation prescription drugs developed through *O*-desmethylvenlafaxine succinate (@Pristiq) has been favored by more and more psychiatrists as their first choice for the treatment of depression and anxiety (Khandpekar et al., 2014; Llorca et al., 2019). Therefore, the improvement of the novel synthesis process and quality standards of desvenlafaxine succinate has great practical significance and market value, attracting more and more synthetic scholars to conduct research (Kamath and Handratta, 2008; Duggirala et al., 2009; Colvard, 2014; Dichiarante et al., 2015; Furlan et al., 2015).

**FIGURE 1:**
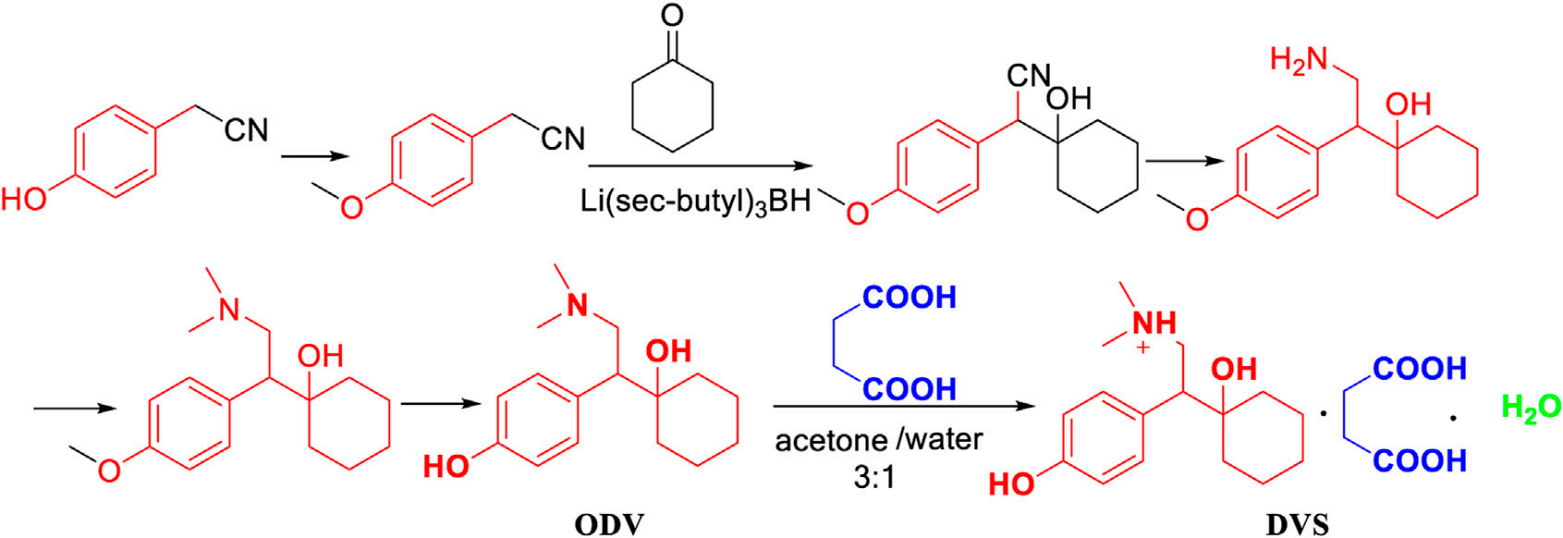
The synthesis of desvenlafaxine catalyzed by Li[sec-butyl]_3_BH.

Hadfield, A. F. (Hadfield et al., 2009) reported that *p*-hydroxybenzene acetonitrile as the starting material was used to produce *p*-methoxybenzene acetonitrile with the action of methylating reagents (Figure 1). In addition, the flammable and irritating compound diphenylphosphine were used in relatively harsh conditions of the cyano group reduction, which does not meet the economic efficiency and is not suitable for industrial production.

**FIGURE 2:**
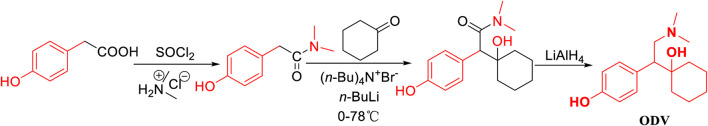
The synthesis of desvenlafaxine from p-hydroxyphenylacetic acid.

Zuo, M. H. (Zuo et al., 2019) reported that the amide compound was prepared by acid halogenation and aminolysis and condensed with cyclohexanone from *p*-hydroxyphenylacetic acid as the starting material (Figure 2). In this method, the operation in the first two steps was complicated due to its use of acyl chloride reagent and dimethylamine aqueous solution in the condensation and amide reduction reaction steps.

**FIGURE 3:**
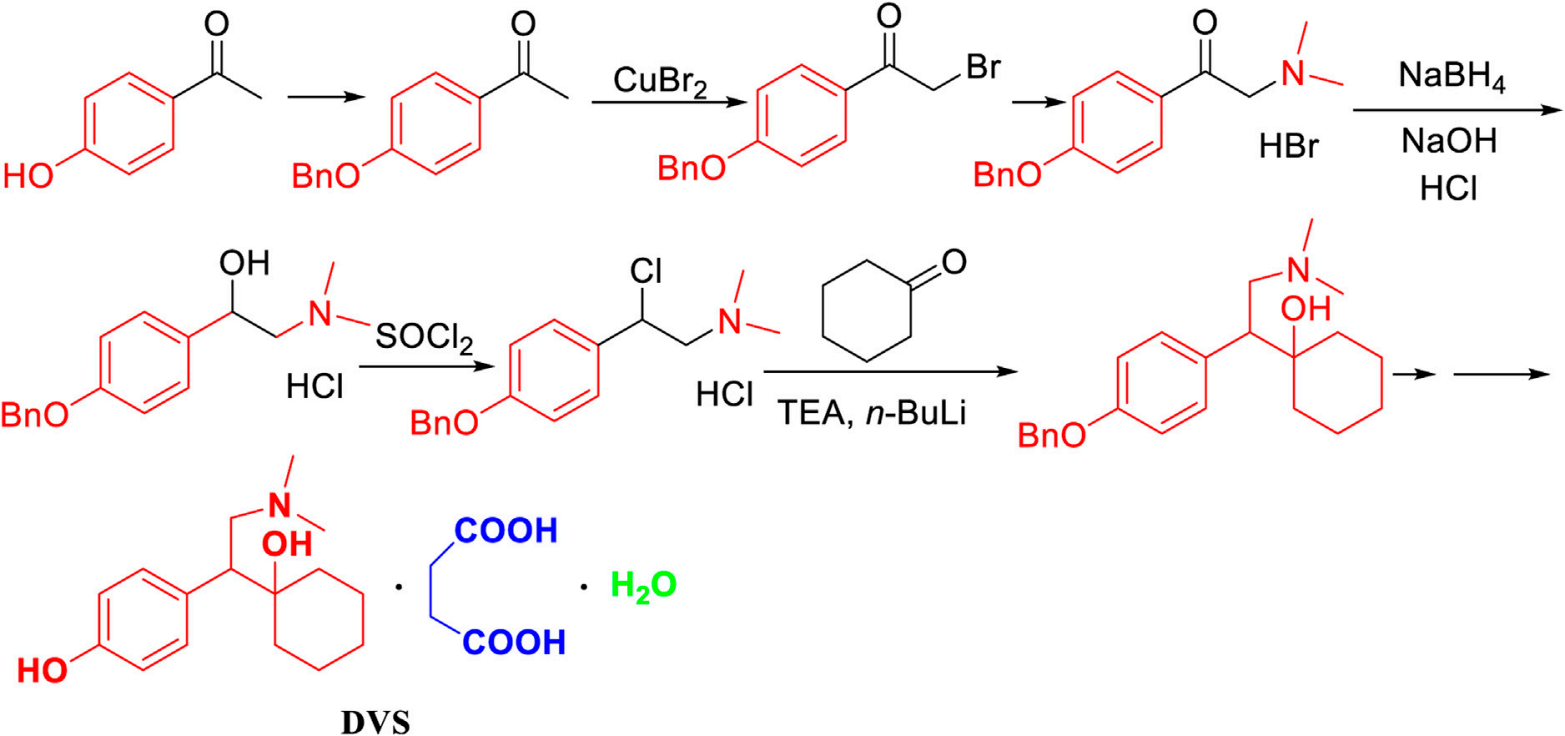
The synthesis of desvenlafaxine from p-hydroxyacetophenone.

Zhao, J. (Zhao et al., 2014) reported that benzyl-protected *p*-hydroxyacetophenone, which was syhthesized by benzyl bromide under the existence of potassium carbonate, was halogenated under the action of copper dibromide and the dimethylation with 33% dimethylamine solution in bromine to prepared 1-(4-(benzyloxy)phenyl)-2-(dimethylamino)ethan-1-one hydrobromide (Figure 3). The method had long reaction steps and low yield. The dimethylamine aqueous solution and hydrogen bromide solution have a negative environmental impact. Moreover, the use of sodium borohydride greatly increases the risk factor of the reaction. The use of the *n*-butyl lithium and 10% Pd/C in the hydrogenation reaction of H_2_ imposes higher requirements on the laboratory, which is not conducive to industrial production.

**FIGURE 4:**
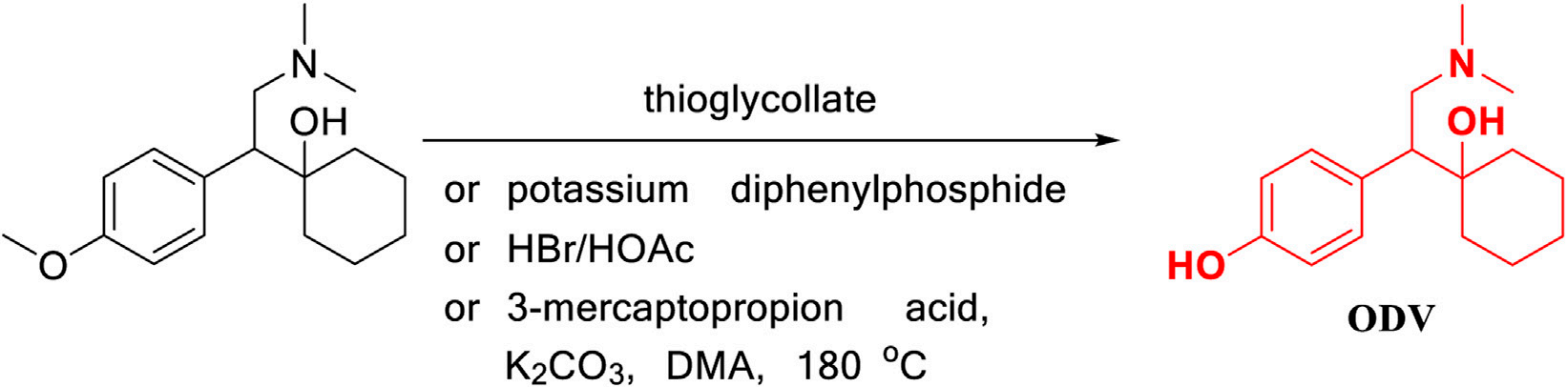
Removal of phenol methyl from venlafaxine.

Jerussi, T. (Jerussi and Senanayake, 2005) reported that *O*-desmethylvenlafaxine was prepared using demethylation reagents such as mercaptan, diphenylphosphine, and HBr hydrobromide to remove phenol methyl from venlafaxine as the starting material (Figure 4). In this method, the demethylation step uses toxic compounds that easily pollute the air, as well as mercaptans, flammable and irritating compounds such as diphenylphosphine, and a more corrosive compound HBr, which increases the difficulty of operation and subsequent production. The treatment process makes this type of reaction unsuitable for industrial production. In addition, Furlan, B. (Furlan et al., 2015) also reported a new strategy of 3-mercaptopropionic acid, which was introduced as a cheap new *O*-demethylating agent as exemplified by its application in the synthesis of antidepressant *O*-desmethylvenlafaxine from venlafaxine. The development of this method provides us with a new direction for the one-step synthesis of desvenlafaxine by venlafaxine.

**FIGURE 5:**
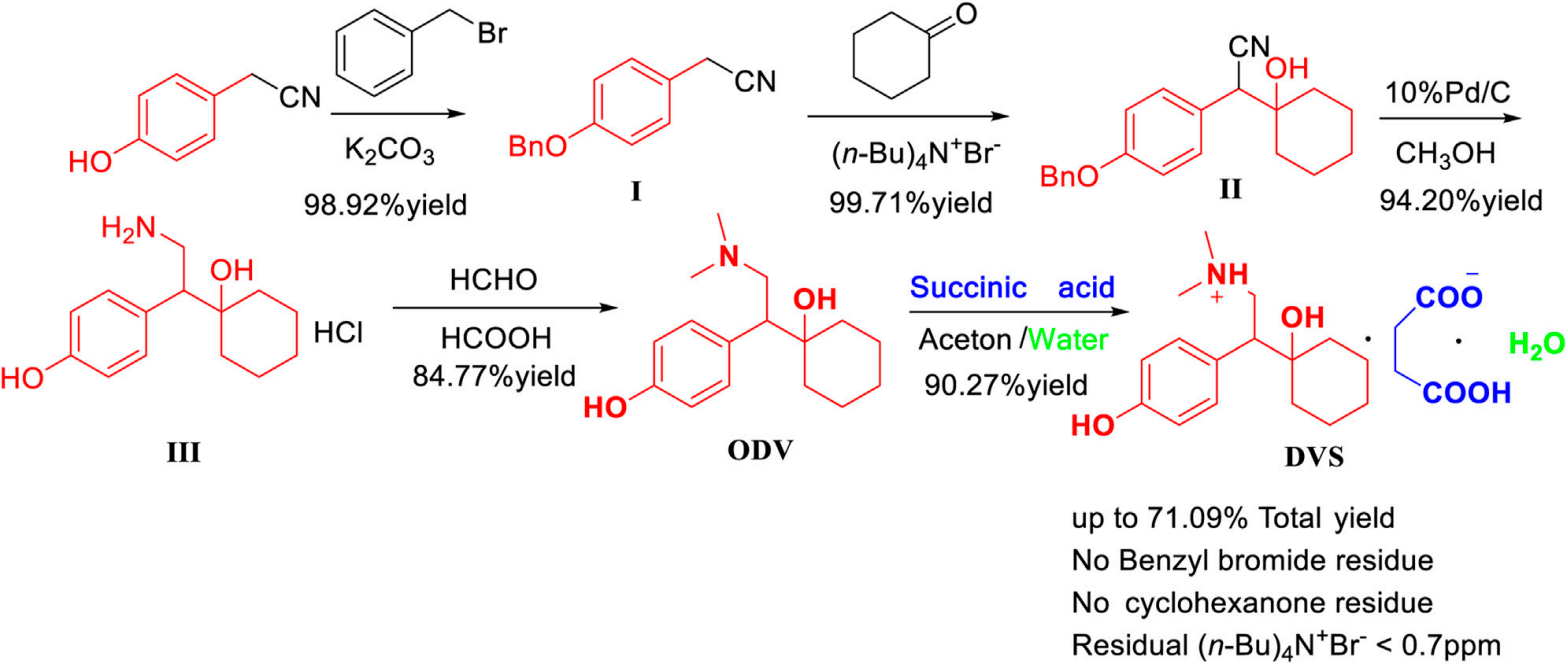
The efficient synthesis of *O*-desmethylvenlafaxine succinate monohydrate (DVS) with high yield and high purity.

The process route method we designed overcomes the problem of cyclohexanone pollution in route five, and it uses *p*-hydroxyphenyl acetonitrile as the starting material to optimize the synthesis of desvenlafaxine with high yield. The yield of each step is high, and the raw materials are easily available. Reagent price is low, and no column chromatography is needed, which is conducive to industrial production (Figure 5).

## Results and Discussion

### Condition Optimization of Intermediate I

The synthesis of Intermediate I was based on *p*-hydroxybenzene acetonitrile as the starting material, under the action of an inorganic base to protect the hydroxyl group of benzyl bromide, wherein the molar equivalent ratio of *p*-hydroxybenzene acetonitrile to benzyl bromide had little effect on the purity of the product but much influence on the product yield. The more benzyl bromide equivalent, the more thorough the reaction. Under the premise of ensuring that the residual excess of benzyl bromide does not exceed the standard, 1.2 equivalent of benzyl bromide is selected for the reaction, and Intermediate I can be obtained with 99.83% purity and 90.10% yield (Table 1, I-2). The reaction time is optimized from 2 to 4 h. The reaction progress of different reaction times is tracked by high performance liquid chromatography. The results show that too short a time is not conducive to the formation of Intermediate I. The reaction time of 4 h reaches the highest reaction progress (Table 1, I-6). The reaction temperature mainly affects the yield of Intermediate I. The higher the temperature, the more favorable the nucleophilic attack of the phenoxy anion on the benzyl methyl cation and the leaving of the bromide ion (Table 1, I-10). The reaction did not produce a solid product in dichloromethane. The purity of acetone, ethyl acetate, and methanol were similar, and the yield of ethyl acetate was not dominant (Table 1, I-14). Under the conditions of similar yield and purity, acetone is the best reaction solvent (Table 1, I-11) from the perspective of atom economy and raw material production cost. Among them, the alkalinity of the inorganic base has a direct impact on the reaction. Too strong an alkalinity will increase by-products and affect the content of related substances and impurities in Intermediate I (Table 1, I-16). The alkalinity is too weak to prevent phenol. The H atom of the hydroxyl group undergoes anionization, thereby reducing the nucleophilic offensive activity of the oxygen anion on the benzyl methyl group of the benzyl bromide, which is not conducive to the departure of the bromide ion. Compared with other works, the synthesis process of Intermediate I has the advantages of higher yield and better purity, and the acetone solvent used can be continuously recovered by rotary evaporator, and has the advantage of continuous recycling after simple treatment.

**TABLE 1:** Condition optimization of Intermediate I.

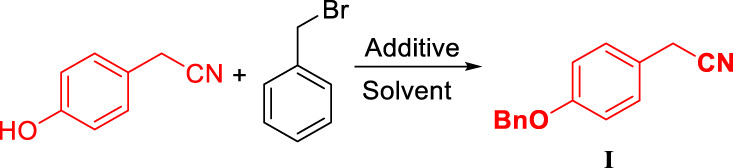								
**No.**	**n(Benzene acetonitrile)**	**n(Benzyl bromide)**	**Time/h**	**T/°C**	**Solvent**	**Additive**	**Purity/%** ^a^	**Yield/%** ^b^ ^,^ ^e^
I-1	1	1.1	3	45	Acetone	K_2_CO_3_	99.60	87.80
**I-2**	**1**	**1.2**	3	45	Acetone	K_2_CO_3_	**99.83**	90.10
I-3	1	1.3	3	45	Acetone	K_2_CO_3_	99.60	90.82
I-4	1	1.2	2	45	Acetone	K_2_CO_3_	75.50^c^	—
I-5	1	1.2	3	45	Acetone	K_2_CO_3_	87.06^c^	—
**I-6**	**1**	**1.2**	**4**	45	Acetone	K_2_CO_3_	**99.56** ^c^	—
I-7	1	1.2	4	25	Acetone	K_2_CO_3_	99.79	80.98
I-8	1	1.2	4	35	Acetone	K_2_CO_3_	99.69	92.19
I-9	1	1.2	4	45	Acetone	K_2_CO_3_	99.71	90.28
**I-10**	**1**	**1.2**	**4**	**55**	Acetone	K_2_CO_3_	**98.93**	**97.94**
**I-11**	**1**	**1.2**	**4**	**55**	**Acetone**	K_2_CO_3_	**98.22**	93.00
I-12	1	1.2	4	55	Methanol	K_2_CO_3_	99.53	89.27
I-13	1	1.2	4	55	DCM	K_2_CO_3_	0^d^	0^d^
I-14	1	1.2	4	55	Ethylacetate	K_2_CO_3_	99.64	86.76
**I-15**	**1**	**1.2**	**4**	**55**	**Acetone**	**K**_**2**_ **CO**_**3**_	**99.42**	**98.92**
I-16	1	1.2	4	55	Acetone	NaOH	99.04	83.96

a Detected by HPLC.

b One recrystallization without column chromatography separation.

c HPLC detects the reaction progress of the reaction solution.

d No solid product is precipitated.

e Isolated yields % = (Actual isolated amount of product/Theoretical amount of product) × 100%.

The meaning of the bold values is to emphasize that this group of data with obvious advantages over other groups, and might be used as a potential optimal condition for further optimization.

### Condition Optimization of Intermediate II

Intermediate I and cyclohexanone are condensed to synthesize Intermediate II under the condition of inorganic base catalysis. Under the action of phase transfer catalyst (*n*-Bu)_4_N^+^Br^−^, the reaction solvent has good yield and purity in water. When the molar equivalent ratio of cyclohexanone to Intermediate I is 2.0, it is beneficial to the 1, 2-nucleophilic addition reaction of Intermediate I to the unsaturated carbonyl group on cyclohexanone. The yield is high, and the residual cyclohexanone is also removed in the subsequent process (Table 2, II-2). (*n*-Bu)_4_N^+^Br^−^ is a genotoxic impurity, so the catalytic dosage is relatively strict. It ensures that the benzyl anion of Intermediate I undergoes a homogeneous reaction with cyclohexanone through phase transfer catalysis, and no more residues are allowed. To mitigate the risk of exceeding the standard, when the equivalent ratio of (*n*-Bu)_4_N^+^Br^−^ to Intermediate I is 0.08, high yield and low residue results can be obtained (Table 2, II-5). Carbanionization of the benzyl methyl group of Intermediate I is also a crucial step, which directly determines the 1, 2-nucleophilic addition reaction activity to the unsaturated carbonyl group on cyclohexanone, so the choice of additives and additive equivalents is also very important. The condensation product was synthesized from cyclohexanone and *p*-methoxybenzene acetonitrile catalyzed by Li[sec-butyl]_3_BH. The cyanide was used as the raw material in this method, but at a slightly higher price. The reducing reagent of Li[sec-butyl]_3_BH has a high risk factor for fire or explosion. The results show that when sodium hydroxide is used as an inorganic base, by-products increase at 1.5 equivalents (Table 2, II-9) and, at 0.5 equivalents, the reaction activity decreases and the reaction is incomplete (Table 2, II-7). In addition, the weak alkaline condition of the inorganic base is not conducive to the formation of benzyl methyl carbanion (Table 2, II-20). When the alkalinity is strong, the generated carbanion continues to produce intermediate 1, 2-nucleophilic addition to the unsaturated carbonyl group. Form II, the yield is higher (Table 2, II-21), the reaction is 4 h, the reaction is complete, and the reaction progress of the reaction solution is 95.66% (Table 2, II-12). The 1,2-nucleophilic addition reaction of the carbanion to the unsaturated carbonyl group obtained by the action of sodium hydroxide with the benzyl methyl group of Intermediate I is relatively active, and the reaction temperature at the beginning of the condensation reaction should not be too high to reduce side effects. The formation of the product (Table 2, II-14) was compared with organic solvents as reaction solvents (Table 2, II-16, 17, 18), higher yield and purity could be obtained in water, with no organic waste liquid, and with the process being more green and economical. Moreover, the detection results of (*n*-Bu)_4_N^+^Br^−^ and cyclohexanone showed that cyclohexanone was not detected, and the residual content of (*n*-Bu)_4_N^+^Br^−^ was below the standard, with a minimum of 0.7 ppm, which is of great significance for improving the quality of APIs. Compared with other works, our synthesis process of intermediate II not only has the advantages of higher yield and better purity, but also does not involve the participation of organic solvents, which greatly reduces pollution and is environmentally friendly.

**TABLE 2:** Condition optimization of Intermediate II.

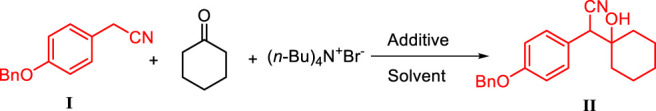									
**No.**	**n(I)**	**n(Cyclohexanone)**	**n((n-Bu)4N + Br-)**	**n(NaOH)**	**Time/h**	**T/^o^C**	**Solvent**	**Purity/%^a^**	**Yield/%^b^ ^*,*^ ^g^**
II-1	1	1.0	0.08	1.0	4	25	H_2_O	94.42	90.00
**II-2**	1	**2.0**	0.08	1.0	4	25	H_2_O	**97.83**	98.59
II-3	1	3.0	0.08	1.0	4	25	H_2_O	95.62	94.64
II-4	1	2.0	0.06	1.0	4	25	H_2_O	96.51	98.43
**II-5**	1	**2.0**	**0.08**	1.0	4	25	H_2_O	**93.15**	**99.71**
II-6	1	2.0	1.00	1.0	4	25	H_2_O	98.85	99.90
II-7	1	2.0	0.08	0.5	4	25	H_2_O	93.50	93.00
**II-8**	1	**2.0**	**0.08**	**1.0**	4	25	H_2_O	**94.18**	96.57
II-9	1	2.0	0.08	1.5	4	25	H_2_O	94.72	92.15
II-10	1	2.0	0.08	1.0	2	25	H_2_O	0.04^c^	—
II-11	1	2.0	0.08	1.0	3	25	H_2_O	72.92^c^	—
**II-12**	1	**2.0**	**0.08**	**1.0**	**4**	25	H_2_O	**95.66** ^c^	—
II-13	1	2.0	0.08	1.0	4	10	H_2_O	96.97	95.57
**II-14**	1	**2.0**	**0.08**	**1.0**	**4**	**25**	H_2_O	**98.69**	**99.71**
II-15	1	2.0	0.08	1.0	4	35	H_2_O	96.97	94.86
II-16	1	2.0	0.08	1.0	4	25	H_2_O	95.51	99.29
II-17	1	2.0	0.08	1.0	4	25	Acetone	5.71^d^	30.01
II-18	1	2.0	0.08	1.0	4	25	CH_3_OH	47.46^d^	78.03
**II-19**	1	**2.0**	**0.08**	**1.0**	**4**	**25**	**H**_**2**_ **O**	**99.13**	95.44
II-20	1	2.0	0.08	1.0^e^	4	25	H_2_O	48.99	2.70
II-21	1	2.0	0.08	1.0^f^	4	25	H_2_O	99.42	95.86

a Detected by HPLC.

b One recrystallization without column chromatography separation.

c HPLC detects the reaction progress of the reaction solution.

d A small amount of solid product is precipitated.

e K_2_CO_3_.

f KOH.

g Isolated yields % = (Actual isolatedamount o produt/Theoretical amount of product) × 100%.

The meaning of the bold values is to emphasize that this group of data with obvious advantages over other groups, and might be used as a potential optimal condition for further optimization.

### Condition Optimization of Intermediate III

**FIGURE 6:**
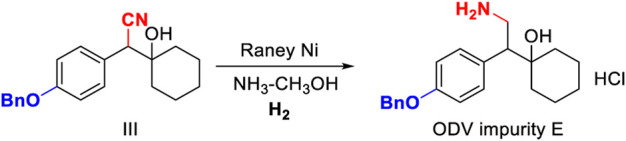
Synthesis and structure confirmation of *O*-desvenlafaxine impurity E.

The deprotection of the benzyl group and the reduction of the cyano group can be achieved by the palladium-carbon hydrogenation method at the same time. The harsh reaction conditions of a low temperature environment was used for the condensation step with *n*-butyl lithium, and used in the last step as the carbonyl reduction reagent reduced the convenience and safety of industrialized production. From the perspective of cost saving, we have optimized the process of palladium-carbon catalyst equivalent and recycling. The catalytic equivalent of 10% palladium-carbon reductant is 0.30, 87% yield (Table 3, III-3) can be obtained, and 0.60 equivalent of recovered palladium-carbon reducing agent has the same reducing effect. The addition of hydrochloric acid is beneficial to form a salt with the amine group in the product, so that the cyano group is continuously reduced to an amine group, and when the equivalent weight is 0.3, a yield of 94.20% and a purity of 94.44% (Table 3, III-4) are obtained. The increase in the equivalent of hydrochloric acid increases the by-products and causes the isomerization of *tertiary* alcohols in an unstable state under acidic conditions (Table 3, III-6). When the benzyl group is deprotected and the cyano group is reduced under pressure, the increase in temperature is favorable for the reaction to proceed (Table 3, III-7, 8, 9). When the protic solvent isopropanol is used as the reaction solvent, more raw materials remain (Table 3, III-12), and methanol has an advantage over ethanol in yield (Table 3, III-10, 11). The reaction time should not be less than 5 h. It can be seen from the results of HPLC tracking the reaction progress of the reaction solution (Table 3, III-13, 14, 15) that the reaction time is short to obtain the by-product desvenlafaxine impurity E, which is not sufficiently reduced (Figure 6).

**TABLE 3:** Condition optimization of Intermediate III.

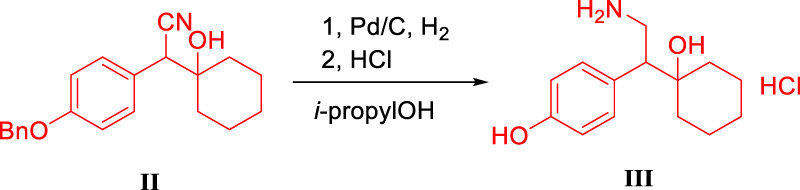								
**No.**	**n(II)**	**10%Pd/C (m/m)**	**HCl (m/v)**	**Time/h**	**T/^o^C**	**Solvent**	**Purity/%** ^a^	**Yield/%** ^b^ ^,^ ^e^
III-1	1	0.50	0.3	5	45	CH_3_OH	0.07	33.97
III-2	1	0.40	0.3	5	45	CH_3_OH	99.11	72.00
**III-3**	**1**	**0.30**	0.3	5	45	CH_3_OH	**98.82**	87.00
**III-4**	**1**	**0.30**	**0.3**	5	45	CH_3_OH	**94.44**	**94.20**
III-5	1	0.30	0.5	5	45	CH_3_OH	91.31	85.13
III-6	1	0.30	1.0	5	45	CH_3_OH	76.79	69.54
III-7	1	0.30	0.3	5	25	CH_3_OH	90.42	77.32
III-8	1	0.30	0.3	5	35	CH_3_OH	83.01	61.48
**III-9**	**1**	**0.30**	**0.3**	**5**	**45**	CH_3_OH	**90.82**	86.75
**III-10**	**1**	**0.30**	**0.3**	**5**	**45**	**CH**_**3**_ **OH**	**81.09**	87.53
III-11	1	0.30	0.3	4	45	Ethanol	86.12	70.90
III-12	1	0.30	0.3	4	45	*i-*propanol	4.07^d^	2.40^d^
III-13	1	0.30	0.3	2	45	CH_3_OH	66.93^c^	—
III-14	1	0.30	0.3	3	45	CH_3_OH	70.69^c^	—
**III-15**	**1**	**0.30**	**0.3**	**4**	**45**	**CH**_**3**_ **OH**	**74.15** ^c^	—

a Detected by HPLC.

b One recrystallization without column chromatography separation.

c HPLC detects the reaction progress of the reaction slution.

d Little amount of solid product is precipitated.

e Isolated yields % = (Actual isolated amount of produt/Theoretical amount of product) x 100%.

The meaning of the bold values is to emphasize that this group of data with obvious advantages over other groups, and might be used as a potential optimal condition for further optimization.

### Condition Optimizationof ODV

The methylation reaction of the amine group is achieved by using formaldehyde solution and intermediate III to form an imine intermediate, and using formic acid as a hydrogen source to reduce the imine intermediate to an amine methyl. However, excess formaldehyde is unfavorable to the reaction, and the best equivalent range is 2.0-3.0 equivalents (Table 4, ODV-1,2,3). When the equivalent of formaldehyde exceeds 4 equivalents, the imine intermediate formed is more stable and not easily destroyed to obtain lower yield (Table 4, ODV-6). Similarly, the equivalent of formic acid is 5 equivalents. By continuing to increase the equivalent of formic acid, the product yield will not increase significantly, and the material cost will be increased at the same time (Table 4, ODV-7, 8, 9). We were surprised to find that the reaction obtained unexpectedly good results when isopropanol was used as the reaction solvent (Table 4, ODV-12, 18). The highest yield was 84.77%, and the highest purity was 99.20%. The liquid progress, product yield, and purity are far higher than the results of using water or methanol as the solvent (Table 4, ODV-10, 11). It is also a protic solvent. Methanol and water are more polar than isopropanol and are more likely to form a solvation effect with Intermediate III, which is not conducive to the formation of imine intermediates of formaldehyde. When formic acid is used as the reaction solvent and when the reaction is carried out under solvation-free conditions, few products are produced and the yield is very low. This also proves that the greater the polarity of the protic solvent, the more unfavorable the progress of the reaction (Table 4, ODV -13).

**TABLE 4:** Condition optimization of ODV.

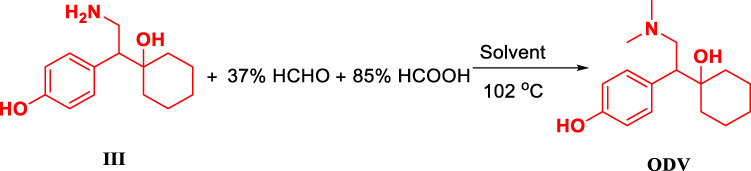							
**NO.**	**n(III)**	**37%HCHO**	**85%HCOOH**	**Solvent**	**Time/h**	**Purity/%^a^**	**Yield/%^b^ ^,^ ^e^**
ODV-1	1	2.0	7.0	H_2_O	24	94.87	64.28
ODV-2	1	2.5	7.0	H_2_O	24	94.17	66.61
ODV-3	1	3.0	7.0	H_2_O	24	94.38	69.64
ODV-4	1	4.0	7.0	H_2_O	24	97.64	57.51
ODV-5	1	5.0	7.0	H_2_O	24	98.77	56.33
ODV-6	1	6.0	7.0	H_2_O	24	98.00	56.17
**ODV-7**	**1**	**3.0**	**5.0**	H_2_O	24	**98.13**	68.74
ODV-8	1	3.0	7.0	H_2_O	24	95.03	69.64
ODV-9	1	3.0	10.0	H_2_O	24	98.11	67.04
ODV-10	1	3.0	5.0	CH_3_OH	24	99.13	48.27
ODV-11	1	3.0	5.0	H_2_O	24	95.12	70.24
**ODV-12**	**1**	**3.0**	**5.0**	***i-*propylOH**	24	**99.20**	84.38
ODV-13	1	3.0	5.0	HCOOH	24	/^d^	—
ODV-14	1	3.0	5.0	*i-*propylOH	8	61.09^c^	57.13
**ODV-15**	**1**	**3.0**	**5.0**	***i-*propylOH**	**24**	**84.18** ^c^	**83.04**
**ODV-16**	**1**	**3.0**	**5.0**	***i-*propylOH**	**28**	**84.02** ^c^	**83.79**
ODV-17	1	3.0	5.0	*i-*propylOH	30	85.19^c^	84.62
ODV-18	1	3.0	5.0	*i-*propylOH	48	**99.02**	**84.77**

a Detected by HPLC.

b One recrystallization without column chromatography separation.

c HPLC detects the reaction progress of the reaction solution.

d A small amount of solid product is precipitated.

e Isolated yields % = (Actual isolated amount of product/Theoretical amount of product) x 100%.

The meaning of the bold values is to emphasize that this group of data with obvious advantages over other groups, and might be used as a potential optimal condition for further optimization.

### Condition Optimization of DVS

The synthesis process of *O*-desmethylvenlafaxine succinate monohydrate crystalline form I is prepared by complete solution reaction and natural cooling and crystallization in a mixed solvent of acetone and water, and then purified by acetone cleaning to obtain qualified products. When the volume-to-mass ratio of the reaction solvent is 10 times the amount, 0.5 equivalent of succinic acid can obtain 99.72% purity and 59.47% yield (Table 5, DVS-2). The results of the reaction time of 2–4 h (Table 5, DVS-4, 5, 6) show that the longer the reaction time, the more conducive to crystallization, with a highest purity of 99.90% and a yield of 90.27% (Table 5, DVS-12) possible to obtain. Since *O*-desmethylvenlafaxine succinate monohydrate has a certain solubility in water, the more the amount of solvent, the less the product will be precipitated, and reducing the amount of solvent has a good effect on the product yield (Table 5, DVS-8). By changing the cleaning solvent acetone to a mixed solvent of ethyl acetate and isopropanol to achieve the maximum single impurity content control, it was found that as the amount of mixed solvent increased, the purity of the product was 99.91%, and the yield was proportional to the amount of solvent. The inverse ratio shows that the refining effect of the mixed solvent at room temperature is not obvious (Table 5, DVS-13, 14, 15), and the highest refining effect of the mixed solvent under heating conditions is 99.92%, the single impurity content is 0.08%, and the yield is low (Table 5, DVS- 16, 17, 18), which has a greater impact on yield. XRD powder diffraction and DSC-TGA of the products prepared by mixed solvents have done XRD powder diffraction and DSC-TGA to detect the crystal form and crystal water of the product, showing that the crystal form has changed compared with the refining process of acetone (see SI).

**TABLE 5:** Condition optimization of DVS.

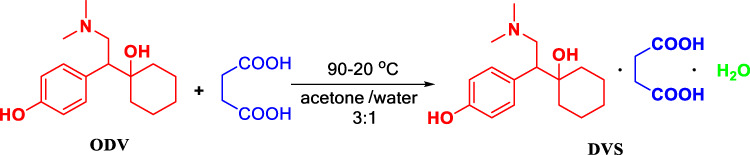						
**No.**	**n(ODV)**	**n(Succinic acid)**	**Time/h**	**Solvent (v/m)**	**Purity/%^a^**	**Yield/%^b^ ^,^ ^i^**
DVS-1	1	0.90	4	10	99.79	51.72
**DVS-2**	**1**	**0.95**	4	10	99.72	59.47
DVS-3	1	1.00	4	10	99.69	58.00
DVS-4	1	0.95	2	10	99.90 (0918)	46.51
DVS-5	1	0.95	3	10	99.90 (0918)	53.97
**DVS-6**	**1**	**0.95**	**4**	10	99.92 (0918)	62.49
DVS-7	1	0.95	4	3	99.90 (0922)	68.74
**DVS-8**	**1**	**0.95**	**4**	**5**	99.90 (0922)	69.64
DVS-9	1	0.95	4	7	99.89 (0922)	67.04
DVS-10	1	0.95	4	5	99.90 (0924)	73.81
DVS-11	1	0.95	5	5	99.90 (0924)	85.95
**DVS-12**	**1**	**0.95**	**6**	**5**	**99.90 (0924)**	**90.27**
DVS-13^c^	1	0.95	6	5	**99.91 (0926)**	**89.46**
DVS-14^d^	1	0.95	6	5	99.90 (0926)	85.17
DVS-15^e^	1	0.95	6	5	99.91 (0926)	82.39
DVS-16^f^	1	0.95	6	5	**99.92 (0927)**	**82.15**
DVS-17^g^	1	0.95	6	5	99.90 (0927)	80.62
DVS-18^h^	1	0.95	6	5	99.90 (0927)	81.79

a Detected by HPLC. (HPLC conditions: the filler is octadecylsilane-bonded silica gel; the mobile phase A is phosphate buffer (adjusted to pH 3.0 with phosphoric acid)-acetonitrile (90:10), and phosphate buffer (adjusted with phosphoric acid) is used as mobile phase A. pH to 3.0)-acetonitrile (40:60) as mobile phase B; detection wavelength at 225 nm; flow rate at 1.0 ml/min; column temperature at 35°C; injection volume at 10 µl).

b One recrystallization without column chromatography separation.

c Change the cleaning solvent from acetone to ethyl acetate and isopropanol (1:1) (5 v/m).

d Change the cleaning solvent from acetone to ethyl acetate and isopropanol (1:1) (10 v/m).

e Change the cleaning solvent from acetone to ethyl acetate and isopropanol (1:1) (15 v/m).

f Crystallization under complete solution conditions in ethyl acetate and isopropanol (1:1) (10 v/m).

g Beating at 50 °C in ethyl acetate and isopropanol (1:1) (10 v/m).

h Beating at room temperature in ethyl acetate and isopropanol (1:1) (10 v/m).

i Isolated yields % = (Actual isolated amount of product/Theoretical amount of product) × 100%.

The meaning of the bold values is to emphasize that this group of data with obvious advantages over other groups, and might be used as a potential optimal condition for further optimization.

## Experimental Section

### Synthesis of 4-Benzyloxyphenylacetonitrile

Add 200.0 g *p*-hydroxybenzene acetonitrile, 282.56 g benzyl bromide, and 1,000 ml acetone to a 3.0 L reaction flask. After the addition is complete, slowly add 320.28 g anhydrous potassium carbonate in batches, stir and react overnight at 55°C, and monitor the reaction by TLC (ethyl acetate: *n*-hexane = 5:1). After the reaction is complete, the solvent is directly removed by rotary evaporation and a large amount of white solid precipitates out. Filter, rinse the filter cake with a large amount of water, and dry to obtain 331.19 g, 98.68% yield. C_15_H_13_NO, ^1^H NMR (400 MHz, Chloroform-*d*) δ 7.45–7.28 (m, 5H), 7.25–7.18 (m, 2H), 6.96 (d, *J* = 8.7 Hz, 2H), 5.05 (s, 2H), 3.65 (s, 2H). ^13^C NMR (101 MHz, Chloroform-*d*) δ 158.57, 136.72, 129.17, 128.69, 128.13, 127.50, 122.14, 118.24, 115.53, 70.14, 22.85.

### Synthesis of 1-[Cyano(4-benzyloxyphenyl)methyl]cyclohexanol

Add 250.0 g of Intermediate I and 500 ml of water to a 1.0 L three-necked reaction flask. Slowly add 176.02 g of cyclohexanone under stirring. After the addition is complete, add 22.0 g of (n-Bu)_4_N^+^Br^−^, and then slowly add 100 ml of 36.0 g sodium hydroxide aqueous solution at the temperature of the reaction solution lower than 5°C, mechanically stir at lower than 20°C for 4 h after the addition is complete, and monitor the reaction by TLC (ethyl acetate: n-hexane = 5:1), a large amount of white solid precipitates, Filter, rinse the filter cake with a lot of water, and dry it to get 359.69g, the yield is 99.94%. C_21_H_23_NO_2_, ^1^H NMR (400 MHz, Chloroform-*d*) δ 7.46–7.35 (m, 4H), 7.33 (dd, *J* = 8.3, 5.5 Hz, 1H), 7.26 (d, *J* = 8.7 Hz, 2H), 6.97 (d, *J* = 8.7 Hz, 2H), 5.06 (s, 2H), 3.72 (s, 1H), 1.71 (d, *J* = 13.1 Hz, 1H), 1.67–1.43 (m, 9H), 1.18 (dd, *J* = 12.7, 6.2 Hz, 1H). ^13^C NMR (101 MHz, Chloroform-*d*) δ 158.97, 136.68, 130.72, 128.69, 128.15, 127.52, 123.98, 119.88, 115.00, 72.77, 70.14, 49.38, 34.98, 34.90, 25.22, 21.59, 21.51.

### Synthesis of 1-[2-amino-1-(4-hydroxyphenyl)ethyl]cyclohexanol Hydrochloride

Add 300.0 g of Intermediate II, 3,000 ml of industrial methanol to a 5.0 L high pressure hydrogenation reactor, slowly add 99.99 g 10% Pd/C under stirring. Then slowly add 90.00 ml of concentrated hydrochloric acid, reacting at 45°C and 2.0 MPa pressure for 4 h, monitored by TLC (ethyl acetate: n-hexane = 2.5:1). After the reaction is complete, the solvent is directly removed by rotary evaporation and a large amount of white solid precipitates. The solid is recrystallized twice with ethyl acetate and dried to obtain 179.90 g. The yield is 82.52%. C_14_H_22_NO_2_Cl, ^1^H NMR (400 MHz, DMSO-*d6*) δ 9.43 (s, 1H), 7.78 (s, 3H), 7.06 (s, 2H), 6.75 (s, 2H), 4.46 (s, 1H), 3.06 (t, *J* = 11.8 Hz, 1H), 2.82 (s, 1H), 1.78–0.86 (m, 11H). ^13^C NMR (101 MHz, DMSO-*d6*) δ 157.00, 131.02, 128.53, 115.51, 72.24, 53.20, 36.80, 33.81, 25.81, 21.89, 21.57.

### Synthesis of *O*-Desvenlafaxine

Add 150.0 g of Intermediate III and 1,500 ml of isopropanol to a 3,000 ml single-necked reaction flask. Slowly add 153.30 g of 37% formaldehyde solution with stirring, stir for 2 h at 20°C, and then slowly add 176.70 g of 85% formic acid solution, at 103°C. The reaction is monitored by TLC (dichloromethane: methanol: trimethylamine = 2.5:10:1.0 drop). After the reaction is complete, the solvent is removed by rotary evaporation to a syrupy state, 500 ml of water is added, and filtered to remove floating insoluble matter. The filterate is added for cleaning with 300 ml of toluene, liquid separation, and removal. Water layer is adjusted to pH 9.5 with 50% sodium hydroxide solution and filtered to obtain crude solids. Recrystallize once with 1,500 ml water at 90°C, filter the residue with 300 ml ethyl acetate, and then 300 ml isopropyl alcohol is recrystallized once at 70°C, recrystallized twice, and dried to obtain 146.47 g, with a yield of 87.70%. C_16_H_25_NO_2_, ^1^H NMR (400 MHz, DMSO-*d6*) δ 9.11 (s, 1H), 6.96 (d, *J* = 6.9 Hz, 2H), 6.64 (d, *J* = 6.9 Hz, 2H), 5.41 (s, 1H), 3.12–2.86 (m, 1H), 2.72 (s, 1H), 2.40–2.25 (m, 1H), 2.14 (s, 6H), 1.61–1.25 (m, 8H), 1.19–0.81 (m, 3H). ^13^C NMR (101 MHz, DMSO-*d6*) δ 156.04, 132.18, 130.55, 114.87, 73.03, 60.92, 52.10, 45.77, 37.64, 32.86, 26.18, 21.74, 21.69. ESI[M + H]^+^ 264.10.

### Synthesis of O-Desmethylvenlafaxine Succinate Monohydrate (DVS)

Add 64 g desvenlafaxine, 27.30 g succinic acid, 300 ml acetone, and 100 ml water to an 1,000 ml round bottom flask, and stir well. Slowly raise the temperature to 90°C and stir for 1 h to dissolve all into a colorless and transparent shape. Cool down to 60°C and filter to remove trace insoluble matter. Continue to lower the temperature to 35°C, stir for 6 h, and make the product fully discharged. Cool and filter in an ice-water bath, wash the filter cake with 100 ml ethyl acetate and 100 ml isopropanol, drain well, and dry at 60°C for 3 h to obtain the finished product of desvenlafaxine succinate monohydrate, a total of 82.40 g. The yield is 85.83%. C_20_H_32_NO_7_, ^1^H NMR (400 MHz, D_2_O) δ 7.19 (d, *J* = 8.3 Hz, 2H), 6.83 (d, *J* = 8.7 Hz, 2H), 3.64 (t, *J* = 12.5 Hz, 1H), 3.51 (dd, *J* = 13.1, 4.0 Hz, 1H), 2.97 (dd, *J* = 12.0, 4.0 Hz, 1H), 2.76 (s, 3H), 2.70 (s, 3H), 2.44 (s, 4H), 1.60 (d, *J* = 13.4 Hz, 1H), 1.48–1.38 (m, 3H), 1.35 (dt, *J* = 7.3, 4.2 Hz, 1H), 1.33–1.23 (m, 2H), 1.19 (dd, *J* = 10.3, 4.5 Hz, 2H), 1.06–0.98 (m, 1H). ^13^C NMR (101 MHz, Deuterium Oxide) δ 179.65, 155.41, 127.44, 115.69, 73.31, 58.36, 50.32, 45.04, 41.37, 35.00, 33.87, 31.31, 24.85, 21.23, 20.99.

### Synthesis and Structure Confirmation of *O*-Desvenlafaxine Impurity E

Add 1-[Cyano(4-benzyloxyphenyl)methyl]cyclohexanol 20.00 g, 15.00 g Raney nickel, and 18 g amine methanol solution to 1.0 L hydrogenation reactor, ventilate at 1.50 MPa, and react at 55°C and 2.0 Mpa pressure for 4 h. After the reaction, lower the temperature to below 30°C, remove the remaining gas, open the autoclave, and remove the reaction solution. Remove the solvent by rotary evaporation to a syrupy state, add ethyl acetate and stir uniformly. Slowly add concentrated hydrochloric acid under ice-water bath conditions, and a white solid precipitates. The white solid is filtered, the filter cake is washed twice with 100 ml ethyl acetate, and dried at 55°C for 2 h to obtain 16.77 g with a yield of 99.70%. C_21_H_27_NO_2_, ^1^H NMR (400 MHz, DMSO-*d6*) δ 7.84 (s, 3H), 7.50–7.44 (m, 2H), 7.44–7.38 (m, 2H), 7.38–7.28 (m, 1H), 7.22 (d, *J* = 8.5 Hz, 2H), 6.98 (d, *J* = 8.7 Hz, 2H), 5.08 (s, 2H), 3.15–3.05 (m, 1H), 2.99–2.82 (m, 1H), 1.57 (d, *J* = 10.5 Hz, 2H), 1.49–1.37 (m, 3H), 1.34–1.13 (m, 3H), 1.12–0.98 (m, 2H).^13^C NMR (101 MHz, DMSO-*d6*) δ 157.98, 137.68, 131.24, 130.79, 128.93, 128.33, 128.22, 128.10, 114.77, 72.15, 69.66, 53.04, 36.76, 34.07, 25.78, 21.89, 21.57.

## Conclusion

Through our continuous optimization of the existing process, the high yield and high purity synthetic preparation of Intermediate I and Intermediate II is obtained. The process of Intermediate II is more green, and there is no organic waste liquid to eliminate the pollution impact on the environment. At the same time, the residues of benzyl bromide and (n-Bu)_4_N^+^Br^−^ with genotoxic impurities are below the standard limit, which improves the quality of raw materials and makes the drug market safer. The preparation of Intermediate III adopts a low pressure, normal temperature, and dilute acid process conditions, which to a certain extent reduces the safety risk of high pressure and high temperatures in the reduction and hydrogenation of difunctional groups, simplifies post-processing and refining operations, and is more conducive to large-scale industrial production. The preparation process of *O*-desvenlafaxine was revealed that isopropanol, as a protic solvent, exhibits special advantages in the dimethylation step, and isopropanol can also be recycled and reused. The method can prepare desvenlafaxine succinate with high yield and high purity, and different new crystal forms can be obtained by using different refining conditions, which has important reference significance for formulation process development in formulation research and development.

## Data Availability

The original contributions presented in the study are included in the article/Supplementary Material, further inquiries can be directed to the corresponding authors.
