# Supplementary material for: Advances in Research at Synthesis Process Optimization and Quality Standard Improvement of O-desmethylvenlafaxine Succinate
Source: Front Chem. 2022 Aug 17;10:860292. doi: 10.3389/fchem.2022.860292 (PMC9428404; doi:10.3389/fchem.2022.860292)
Supplement: Supplementary file 1 [file DataSheet1.PDF]

Supplementary Material

Advances in Research at Synthesis Process Optimization and Quality Standard Improvement of Desvenlafaxine Succinate

Yang Shiwei[1,*], Chen Shiyun[3], Wang Cheng[1], Shibo Zhang[1], Shuaifei Li[1], Yuan Xinsong[1], Peng Fuyun[1], He Yong[2,*]

[1] Department of Chemistry and Chemical Engineering, Hefei Normal University, 230061, Hefei, Anhui, China

[2] Hefei University of Technology, 230009, Hefei, Anhui, China

[3] Hefei University, 230601, Hefei, Anhui, China

* Correspondence:

Corresponding Author

E-mail: yangsw@hfnu.edu.cn; h13956000400@163.com

SI-1. ^{1}H NMR Spectrum of 4-Benzyloxyphenylacetonitrile 5
SI-2. ^{13}C NMR Spectrum of 4-Benzyloxyphenylacetonitrile 5
SI-3. ^{1}H NMR Spectrum of 1-[Cyano(4-benzyloxyphenyl)methyl]cyclohexanol 6
SI-4. ^{13}C NMR Spectrum of 1-[Cyano(4-benzyloxyphenyl)methyl]cyclohexanol 6
SI-5. ^{1}H NMR Spectrum of 1-[2-amino-1-(4-hydroxyphenyl)ethyl]cyclohexanol hydrochloride 7
SI-6. ^{13}C NMR Spectrum of 1-[2-amino-1-(4-hydroxyphenyl)ethyl]cyclohexanol hydrochloride 7
SI-8. ^{13}C NMR Spectrum of *O*-Desvenfaxine 8
SI-9. MS Spectrum of *O*-Desvenfaxine 9
SI-10. ^{1}H NMR Spectrum of desvenlafaxine succinate monohydrate 9
SI-11. IR Spectrum of desvenlafaxine succinate monohydrate 10
SI-12. ^{13}C NMR Spectrum of desvenlafaxine succinate monohydrate 10
SI-13. ^{1}H NMR Spectrum of O-desvenlafaxine impurity E 11
SI-14. ^{13}C NMR Spectrum of O-desvenlafaxine impurity E 11
SI-15. MS Spectrum of O-desvenlafaxine impurity E 12
SI-16. XRD Spectrum of desvenlafaxine succinate monohydrate(Original research patent) 12
SI-17. XRD Spectrum of desvenlafaxine succinate monohydrate (DVS-12) 13
SI-18. XRD Spectrum of desvenlafaxine succinate monohydrate (DVS-16) 13
SI-19. XRD Spectrum of desvenlafaxine succinate monohydrate (DVS-17) 14
SI-20. XRD Spectrum of desvenlafaxine succinate monohydrate (DVS-18) 14
SI-21. HPLC spectrum of I-1 15
SI-22. HPLC spectrum of I-2 16
SI-23. HPLC spectrum of I-3 17
SI-24. HPLC spectrum of I-4 18
SI-25. HPLC spectrum of I-5 19
SI-26. HPLC spectrum of I-6 21
SI-27. HPLC spectrum of I-7 22
SI-28. HPLC spectrum of I-8 23

SI-29. HPLC spectrum of I-9 24
SI-30. HPLC spectrum of I-10 25
SI-31. HPLC spectrum of I-11 26
SI-32. HPLC spectrum of I-12 27
SI-33. HPLC spectrum of I-13 27
SI-34. HPLC spectrum of I-14 28
SI-35. HPLC spectrum of I-15 29
SI-36. HPLC spectrum of I-16 30
SI-37. HPLC spectrum of II-1 31
SI-38. HPLC spectrum of II-2 32
SI-39. HPLC spectrum of II-3 33
SI-40. HPLC spectrum of II-4 34
SI-41. HPLC spectrum of II-5 35
SI-42. HPLC spectrum of II-6 36
SI-43. HPLC spectrum of II-7 37
SI-44. HPLC spectrum of II-8 38
SI-45. HPLC spectrum of II-9 39
SI-46. HPLC spectrum of II-10 40
SI-47. HPLC spectrum of II-11 41
SI-48. HPLC spectrum of II-12 42
SI-49. HPLC spectrum of II-13 43
SI-50. HPLC spectrum of II-14 44
SI-51. HPLC spectrum of II-15 45
SI-52. HPLC spectrum of II-16 46
SI-53. HPLC spectrum of II-17 47
SI-54. HPLC spectrum of II-18 49
SI-55. HPLC spectrum of II-19 50
SI-56. HPLC spectrum of II-20 51
SI-57. HPLC spectrum of II-21 52
SI-58. HPLC spectrum of O-desvenlafaxine impurity E 53
SI-59. HPLC spectrum of ODV-1 54

SI-60. HPLC spectrum of ODV-2 56
SI-61. HPLC spectrum of ODV-3 57
SI-62. HPLC spectrum of ODV-4 59
SI-63. HPLC spectrum of ODV-5 60
SI-64. HPLC spectrum of ODV-6 61
SI-65. HPLC spectrum of ODV-7 62
SI-66. HPLC spectrum of ODV-8 63
SI-67. HPLC spectrum of ODV-9 64
SI-68. HPLC spectrum of ODV-10 65
SI-69. HPLC spectrum of ODV-11 66
SI-70. HPLC spectrum of ODV-12 67
SI-71 HPLC spectrum of ODV-13 68
SI-72. HPLC spectrum of ODV-14 68
SI-73. HPLC spectrum of ODV-15 70
SI-74. HPLC spectrum of ODV-16 71
SI-75. HPLC spectrum of ODV-17 73
SI-76. HPLC spectrum of ODV-18 74
SI-77. HPLC spectrum of DVS-1 75
SI-78. HPLC spectrum of DVS-2 76
SI-79. HPLC spectrum of DVS-3 77
SI-80. HPLC spectrum of DVS-4 78
SI-81. HPLC spectrum of DVS-5 79
SI-82. HPLC spectrum of DVS-6 80
SI-83. HPLC spectrum of DVS-7 81
SI-84. HPLC spctrum of DVS-8 82
SI-85. HPLC spectrum of DVS-9 83
SI-86. HPLC spectrum of DVS-10 84
SI-87. HPLC spectrum of DVS-11 85
SI-88. HPLC spectrum of DVS-12 86
SI-89. HPLC spectrum of DVS-13 87
SI-90. HPLC spectrum of DVS-14 88

SI-91. HPLC spectrum of DVS-15 89

SI-92 HPLC spectrum of DVS-16........ 90

SI-93. HPLC spectrum of DVS-17 91

SI-94. HPLC spectrum of DVS-18 92

SI-1. ^{1}H NMR Spectrum of 4-Benzyloxyphenylacetonitrile

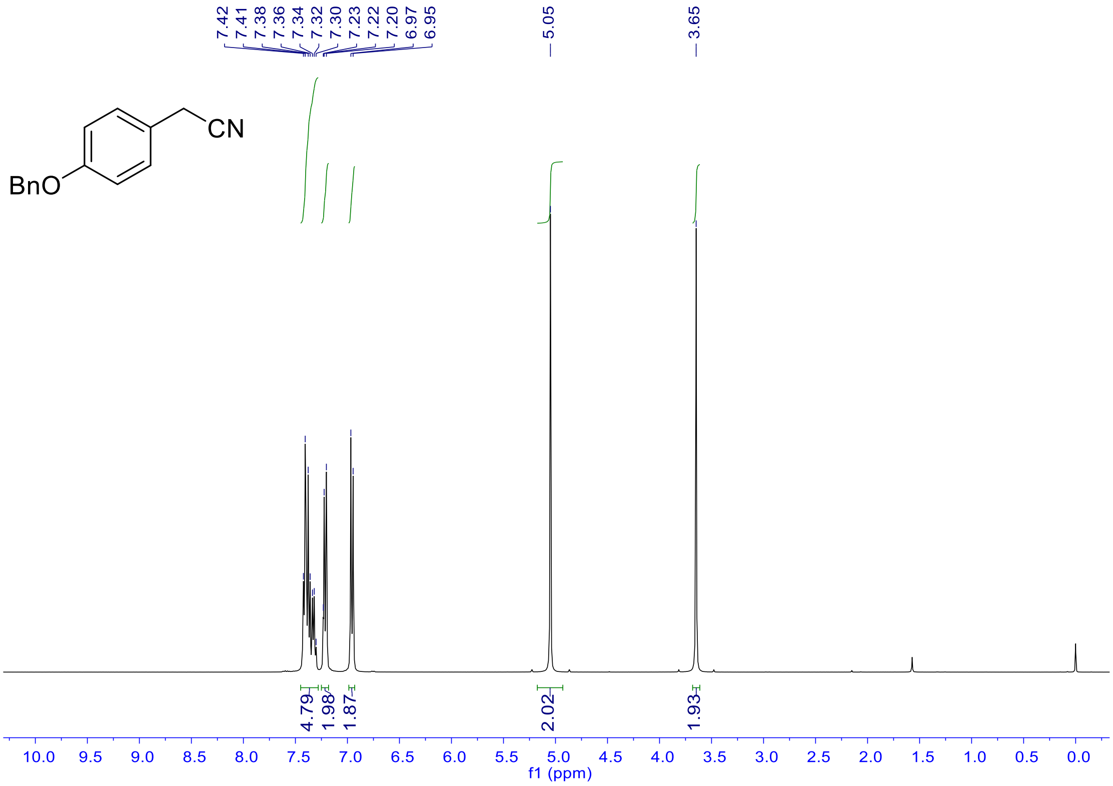

SI-2. ^{13}C NMR Spectrum of 4-Benzyloxyphenylacetonitrile

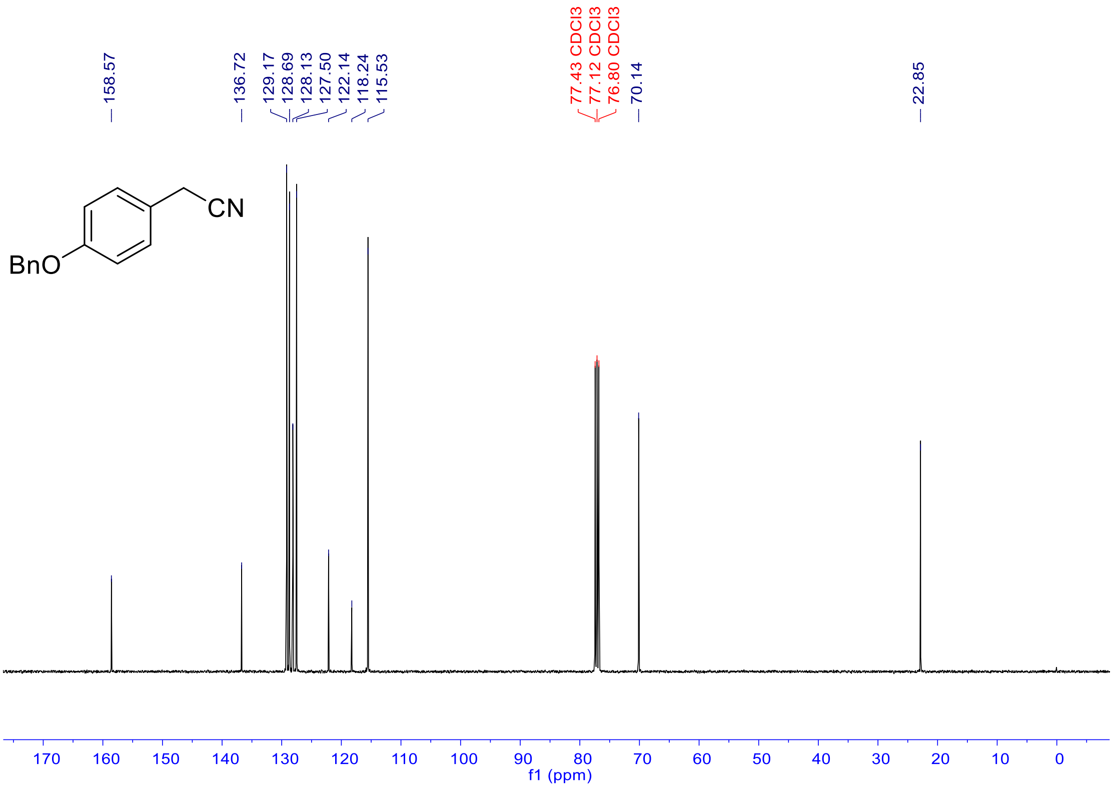

SI-3. ^{1}H NMR Spectrum of 1-[Cyano(4-benzyloxyphenyl)methyl]cyclohexanol

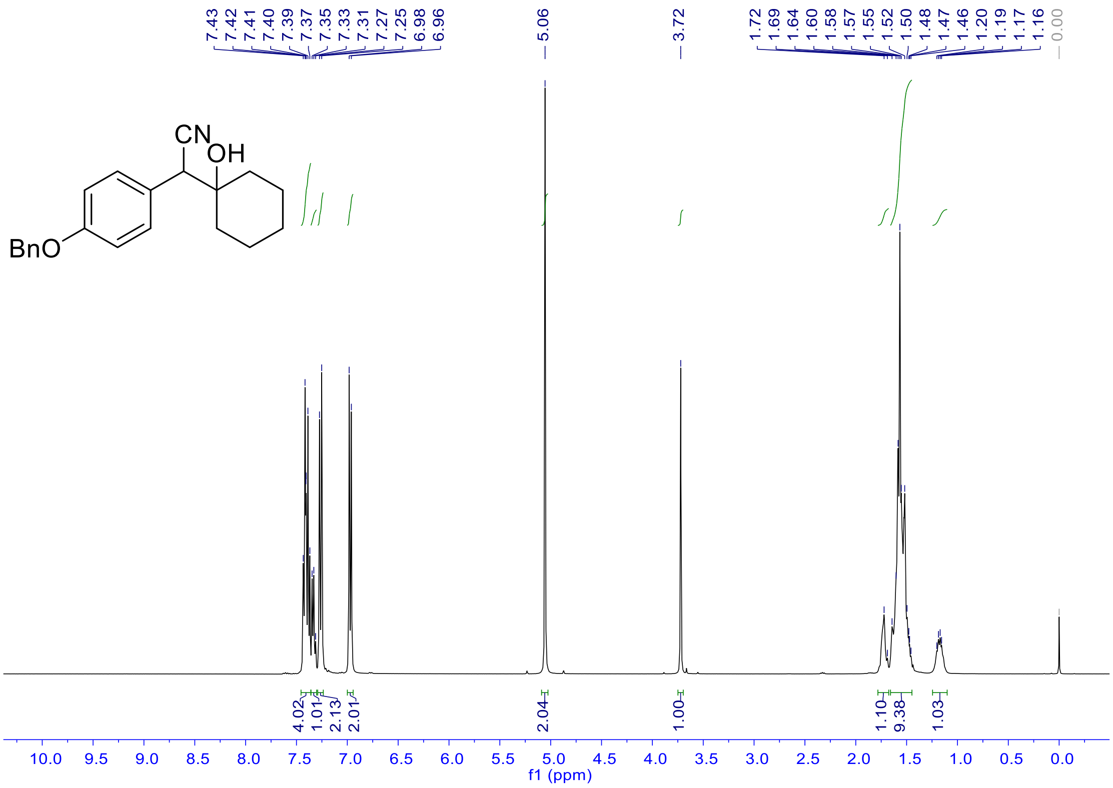

SI-4. ^{13}C NMR Spectrum of 1-[Cyano(4-benzyloxyphenyl)methyl]cyclohexanol

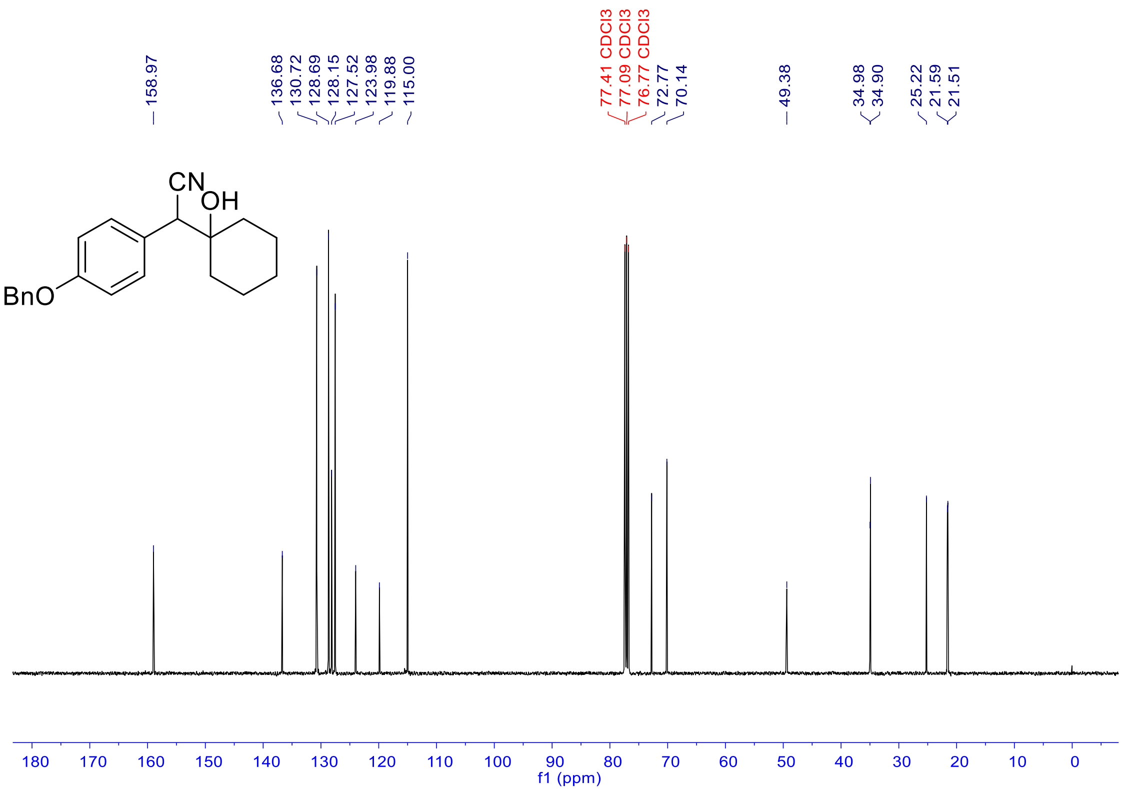

SI-5. ^{1}H NMR Spectrum of 1-[2-amino-1-(4-hydroxyphenyl)ethyl]cyclohexanol hydrochloride

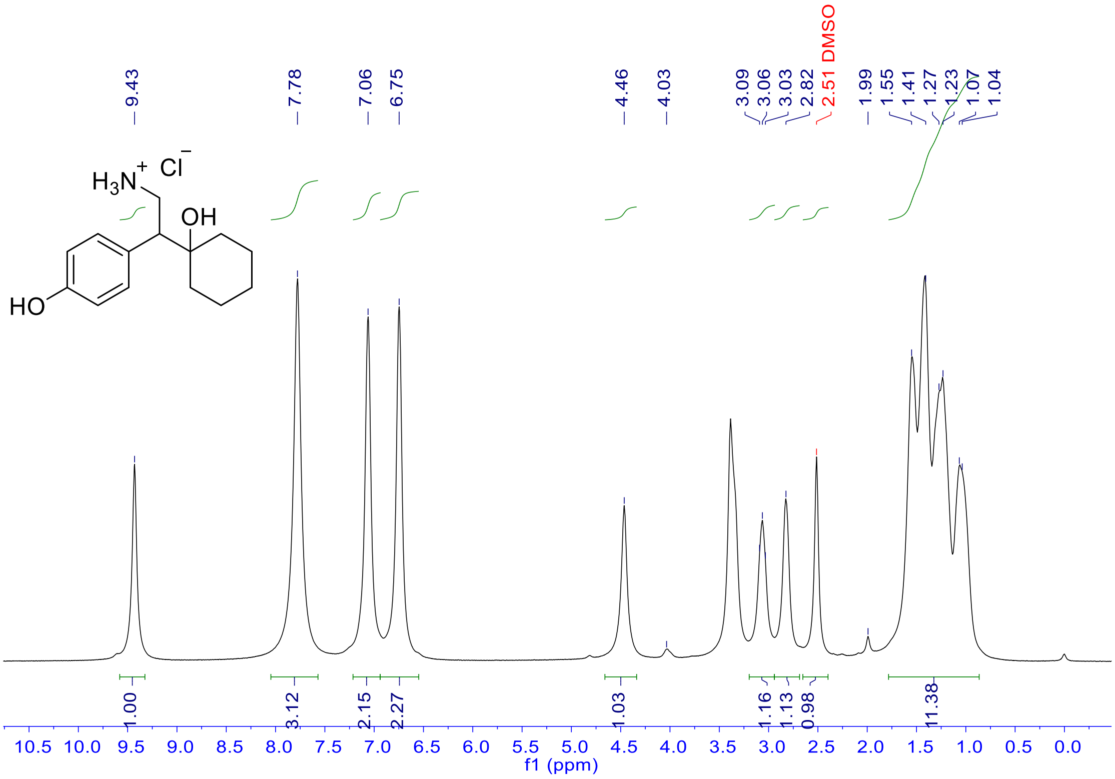

SI-6. ^{13}C NMR Spectrum of 1-[2-amino-1-(4-hydroxyphenyl)ethyl]cyclohexanol hydrochloride

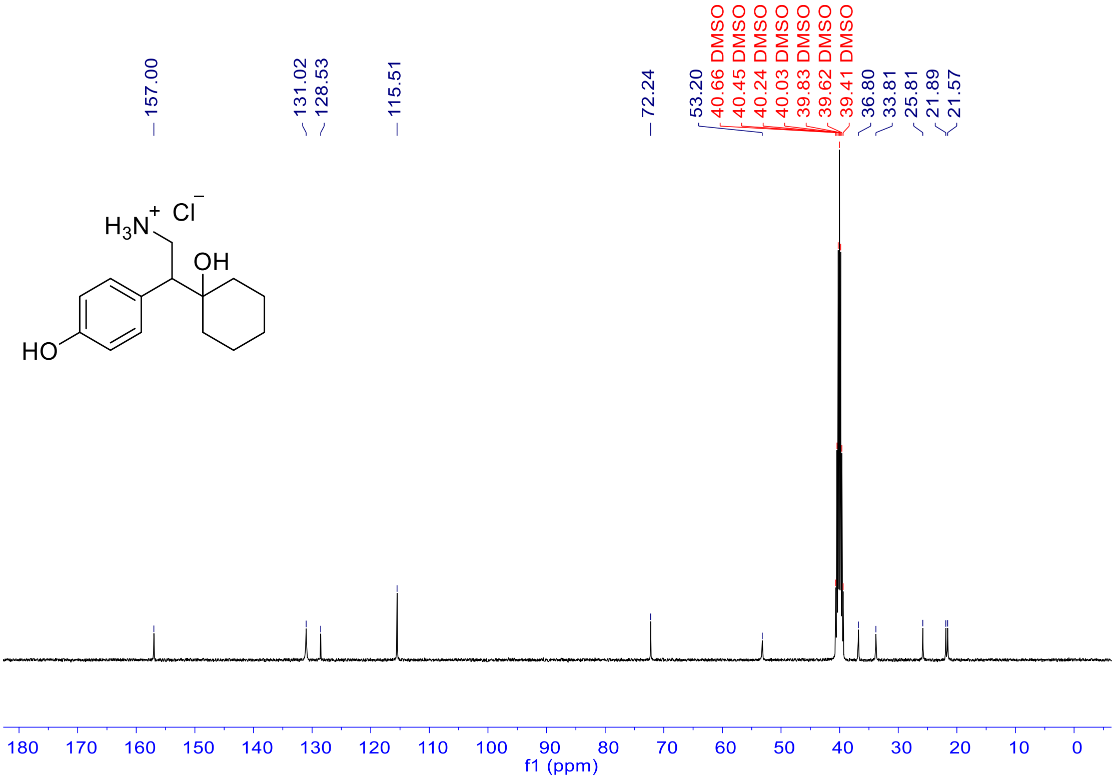

SI-7. ^{1}H NMR Spectrum of *O*-Desvenfaxine

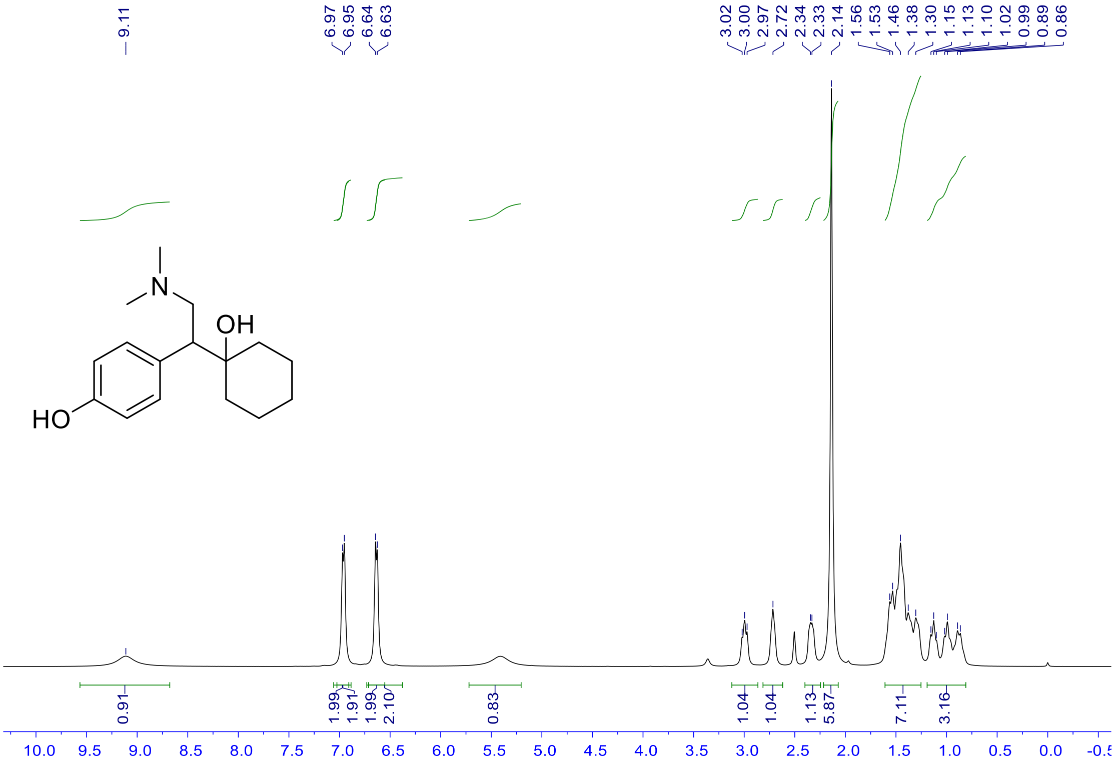

SI-8. ^{13}C NMR Spectrum of *O*-Desvenfaxine

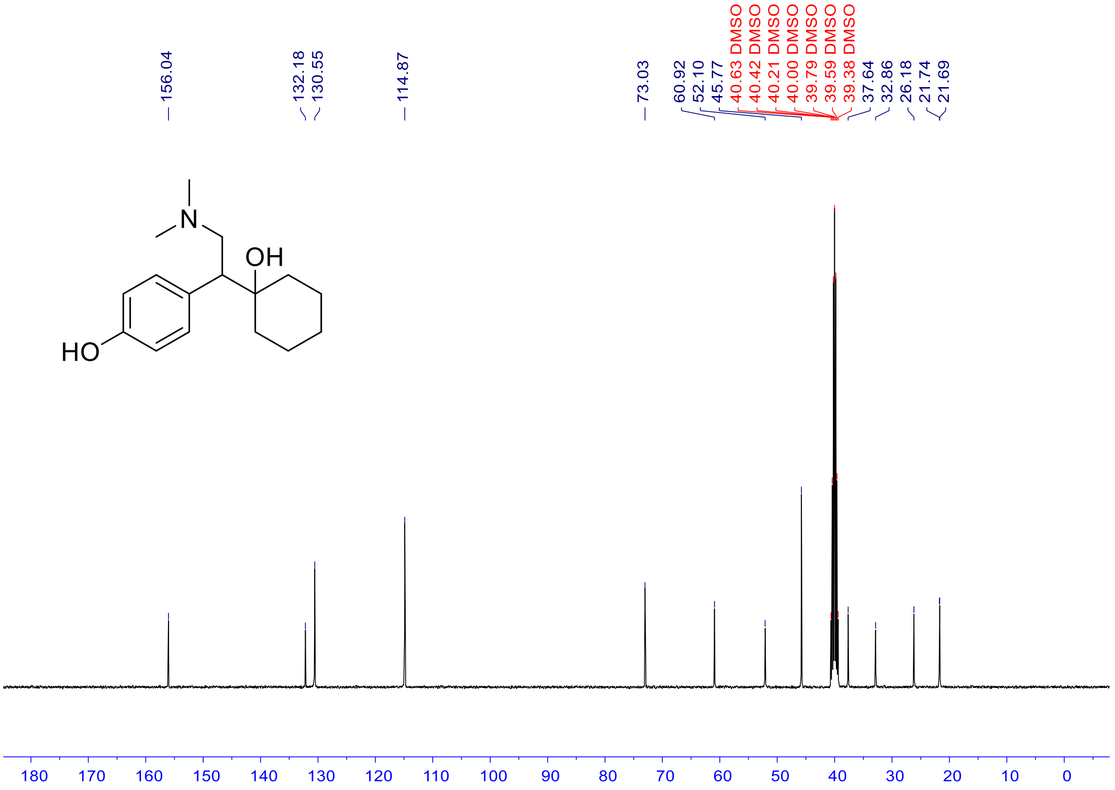

SI-9. MS Spectrum of *O*-Desvenfaxine

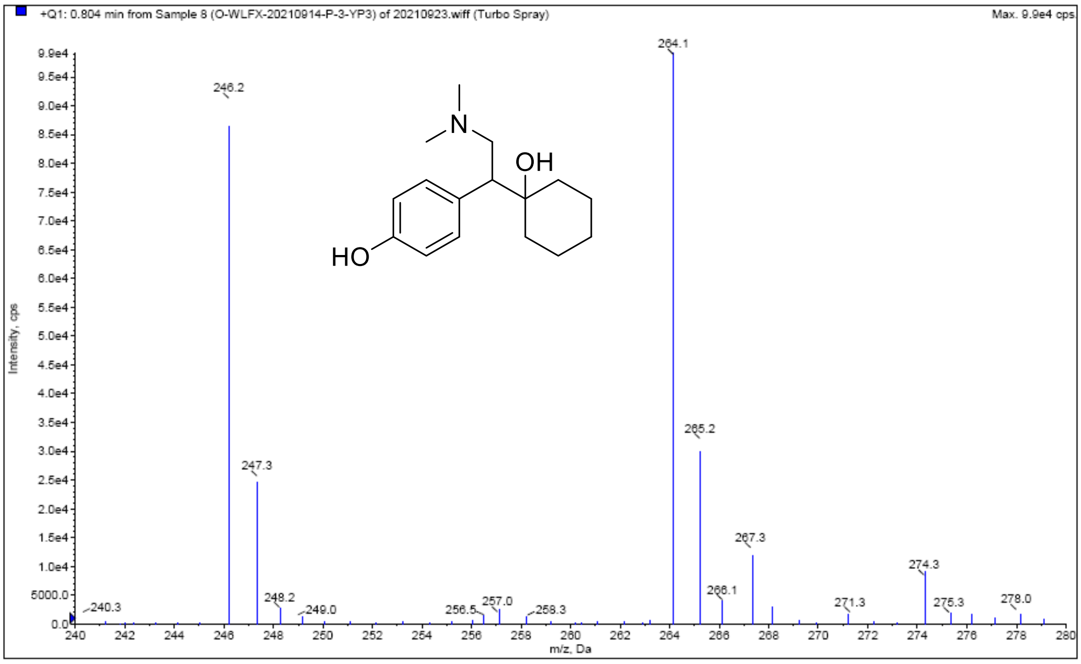

SI-10. ^{1}H NMR Spectrum of desvenlafaxine succinate monohydrate

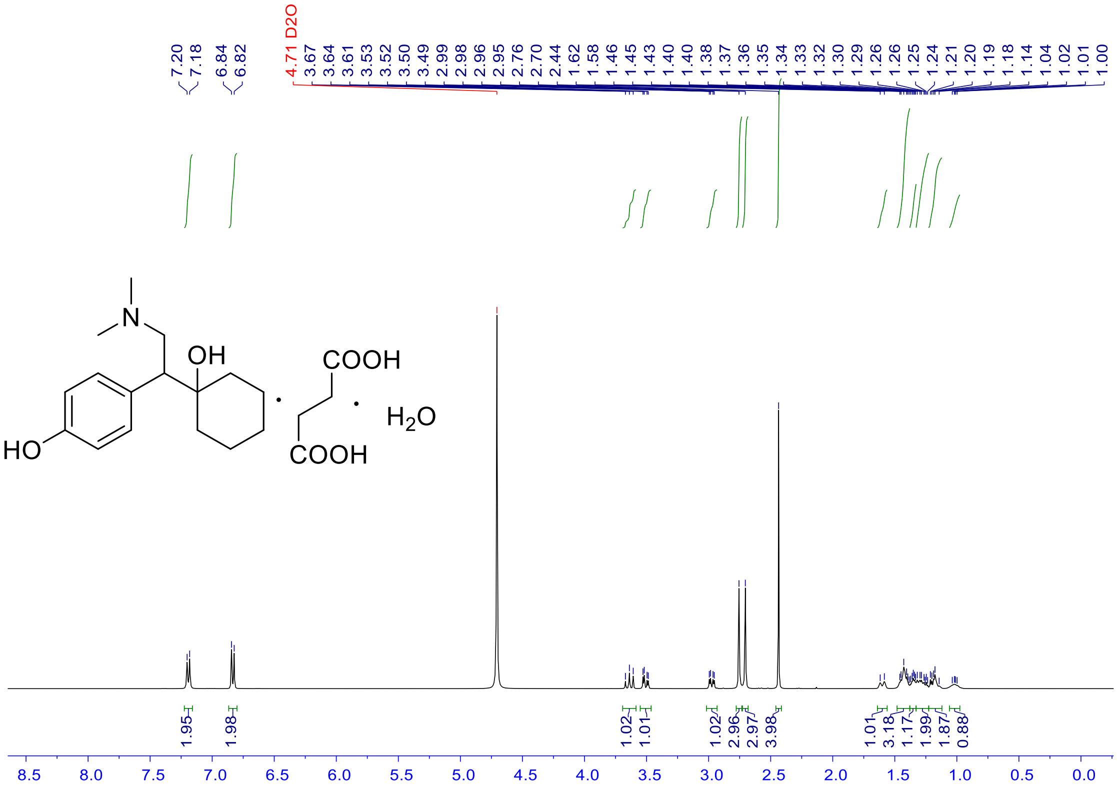

SI-11. IR Spectrum of desvenlafaxine succinate monohydrate

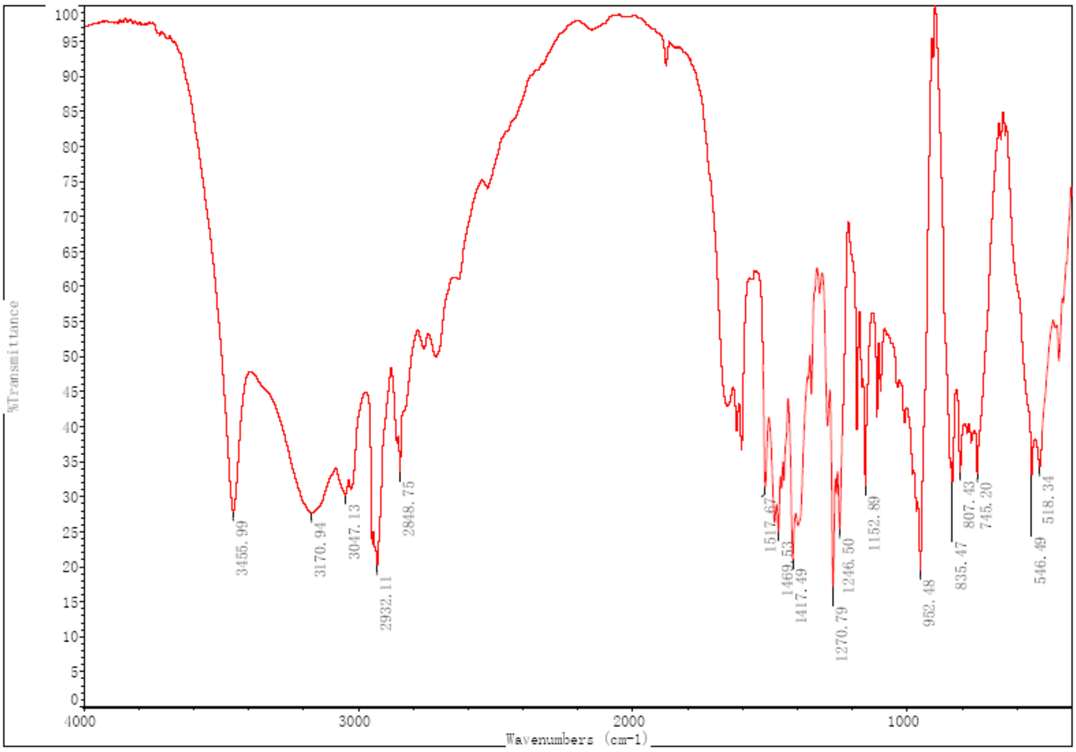

SI-12. ^{13}C NMR Spectrum of desvenlafaxine succinate monohydrate

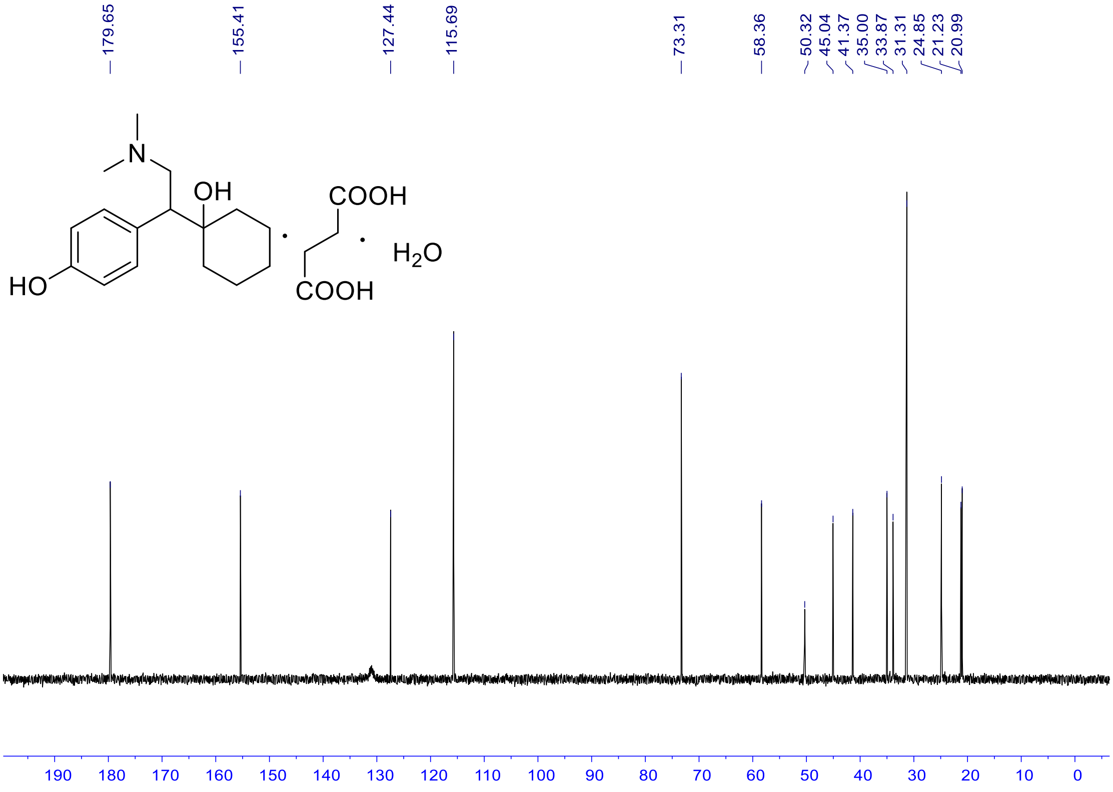

SI-13. ^{1}H NMR Spectrum of O-desvenlafaxine impurity E

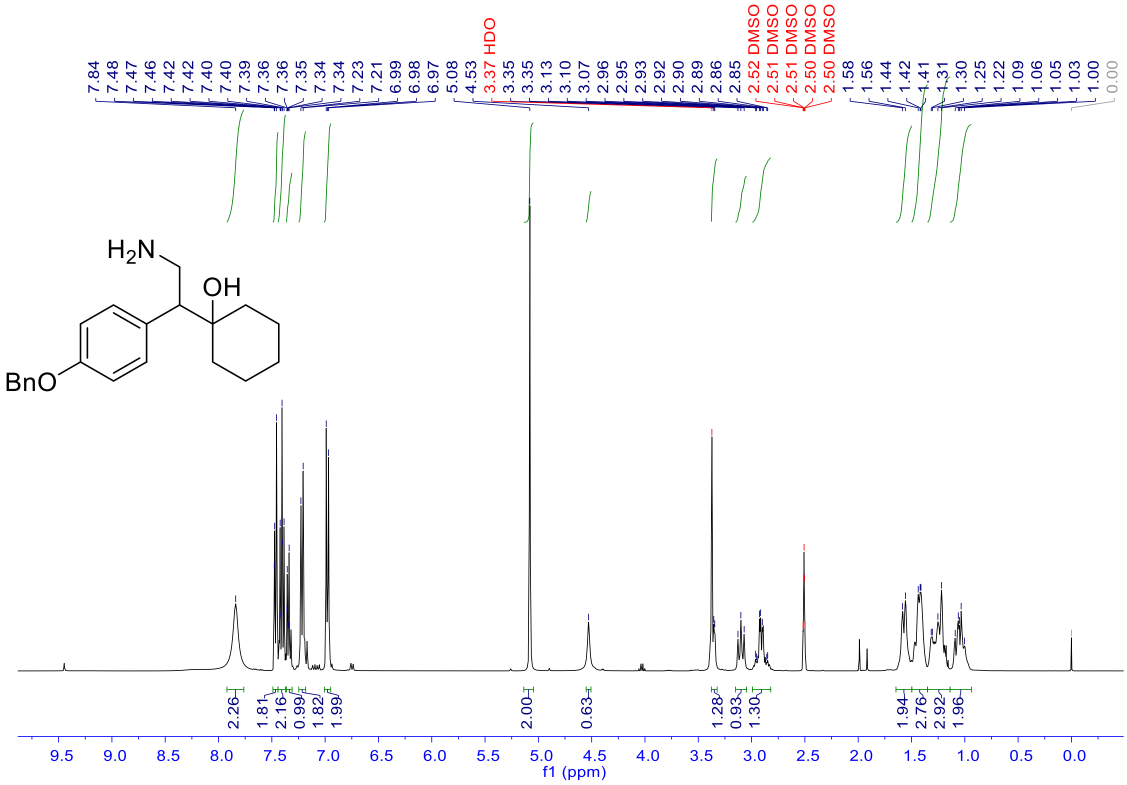

SI-14. ^{13}C NMR Spectrum of O-desvenlafaxine impurity E

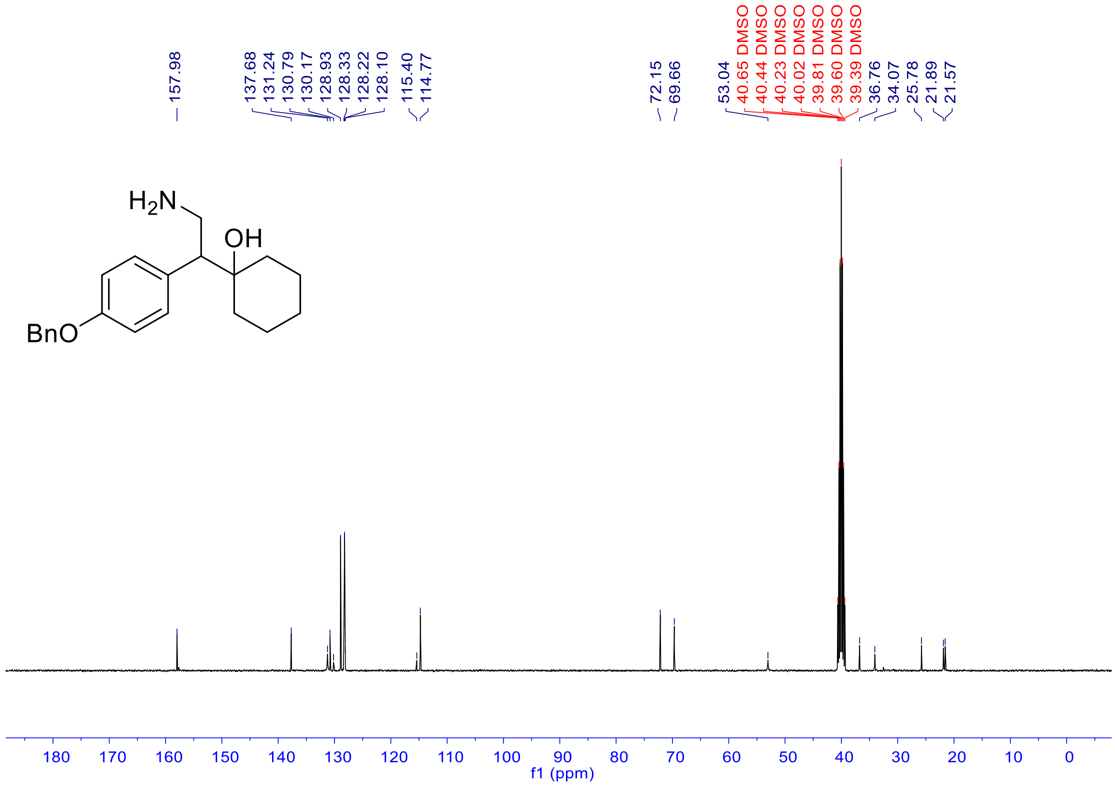

SI-15. MS Spectrum of O-desvenlafaxine impurity E

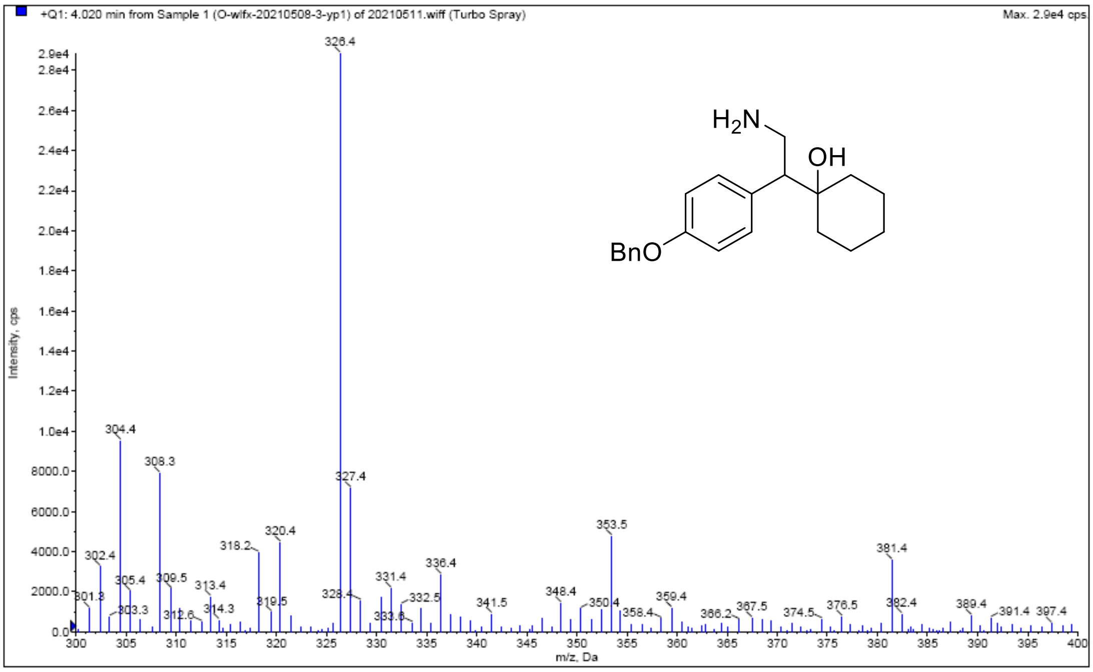

SI-16. XRD Spectrum of desvenlafaxine succinate monohydrate(Original research patent)

Reference: Hadfield A F, Shah S M, Winkley M W, et al. Novel Succinate Salt of O-Desmethyl-Venlafaxine: U.S. Patent Application 11/841,638[P]. 2009-1-15.

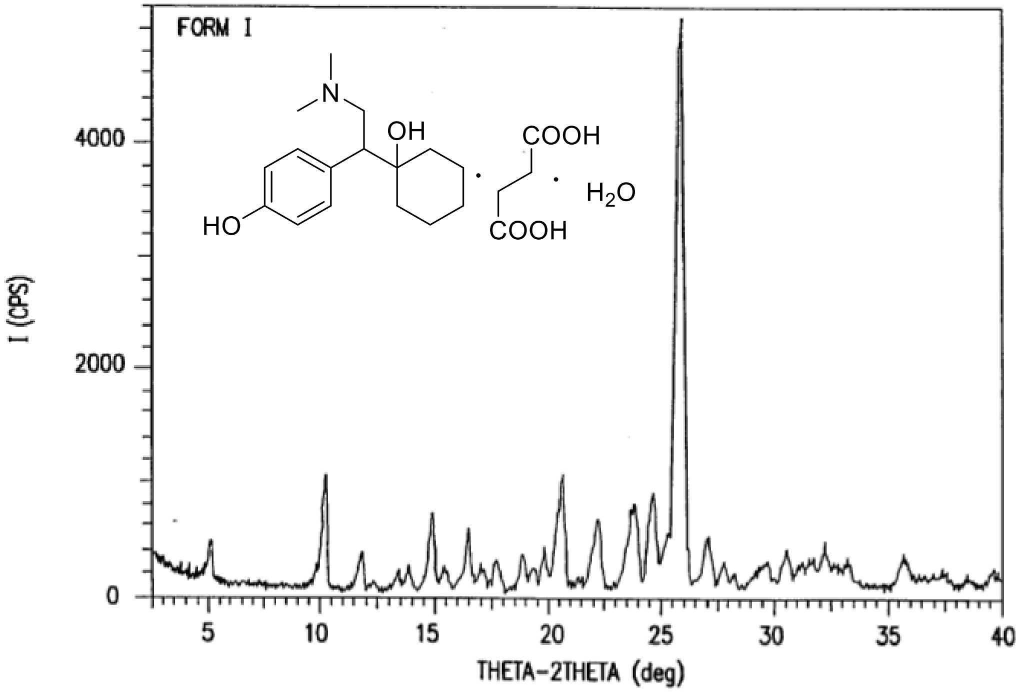

FIG.1

SI-17. XRD Spectrum of desvenlafaxine succinate monohydrate (DVS-12)

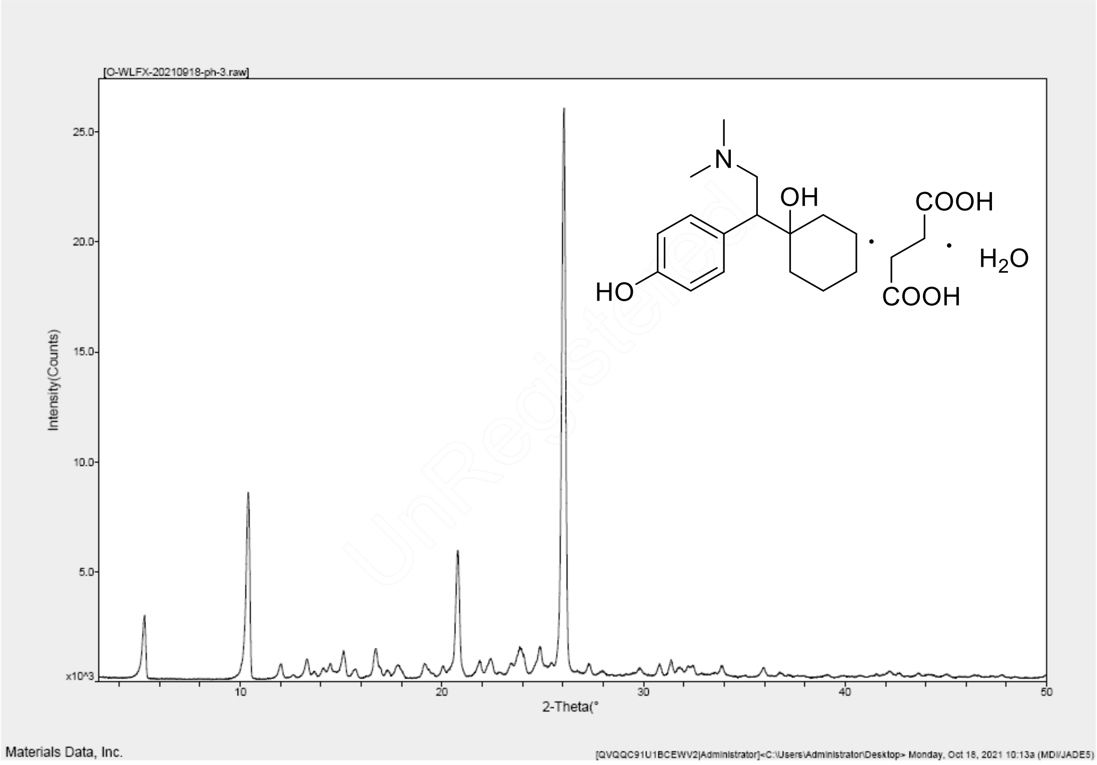

SI-18. XRD Spectrum of desvenlafaxine succinate monohydrate (DVS-16)

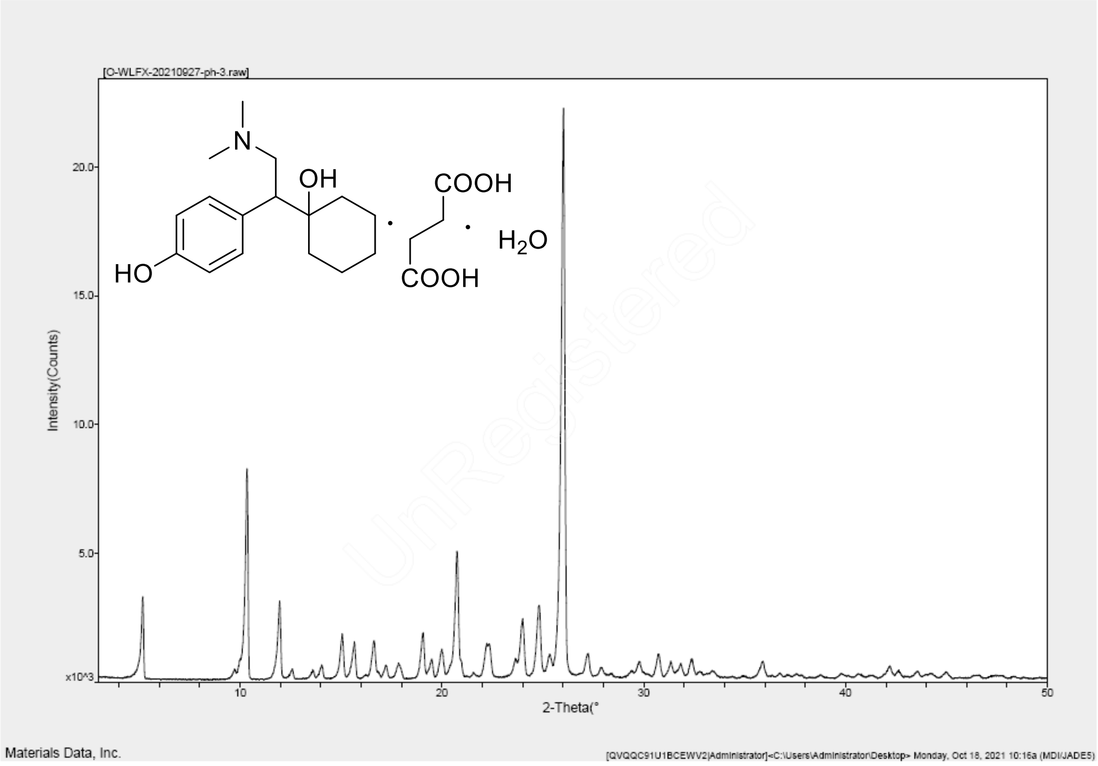

SI-19. XRD Spectrum of desvenlafaxine succinate monohydrate (DVS-17)

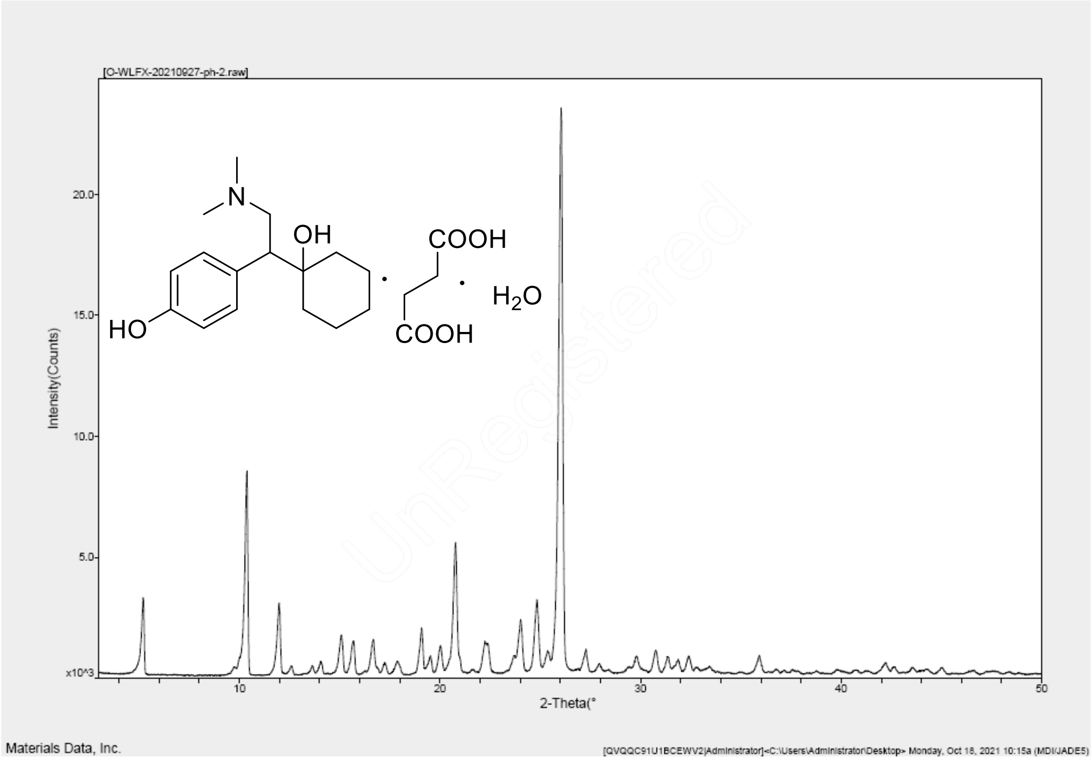

SI-20. XRD Spectrum of desvenlafaxine succinate monohydrate (DVS-18)

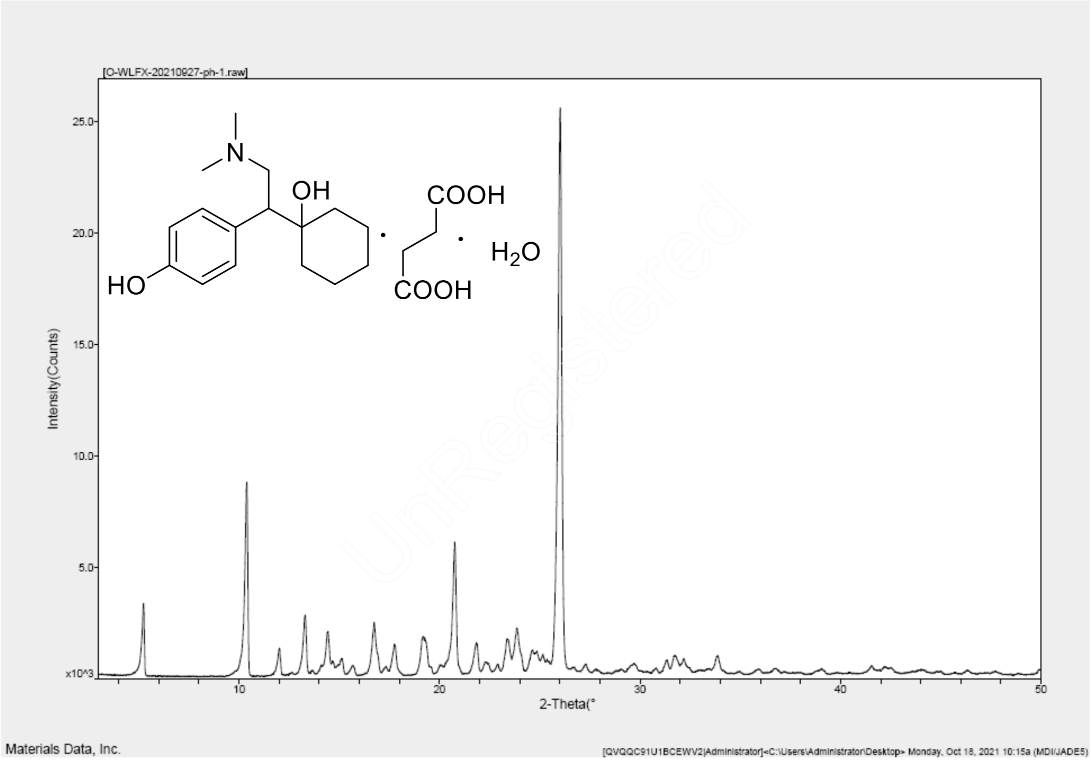

SI-21. HPLC spectrum of I-1

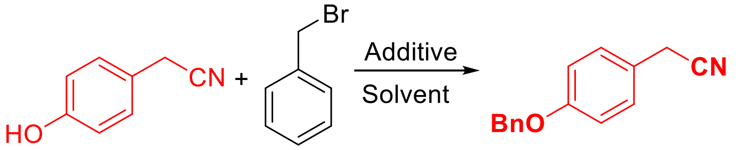

I

琥珀酸去甲文拉法辛分析报告

**Instrument:**	ShimadzuLC08	**Project Name:**	Technology Research
**Manual Modified:**	None	**Operator:**	wujialing
**Sample name:**	intermediate Ⅰ product (O-WLFX-20210427-1-1)	**Inj. volume:**	10
**Location:**	1:6	**Last Modified By:**	wujialing
**Acq. method:**	Related substance method-S08.amx	**Injection Date:**	2021-04-27 16:38:58+08:00
**Acq.Method Version:**	2021-0427-0157-15683	**Modified Date:**	2021-04-28 08:31:05+08:00
**Pro.Method:**	*Related Substance.pmx	**Printied Date:**	2021-04-28 08:35:55+08:00
**Pro.Method Version:**	2021-0428-0030-31077	**Result Set Version:**	2021-0428-0031-17236

Work station:: Agilent OpenLAB CDS Software Version:2.3(Build 2.3.0.468)

Result Path: /Desvenlafaxine Succinate/Technology Research/Results/Related Substance/Intermediate Ⅰ/20210427-intermediate Ⅰ purity detection (synthesis process optimization).rslt

Data file: intermediate Ⅰ product (O-WLFX-20210427-1-1).dx

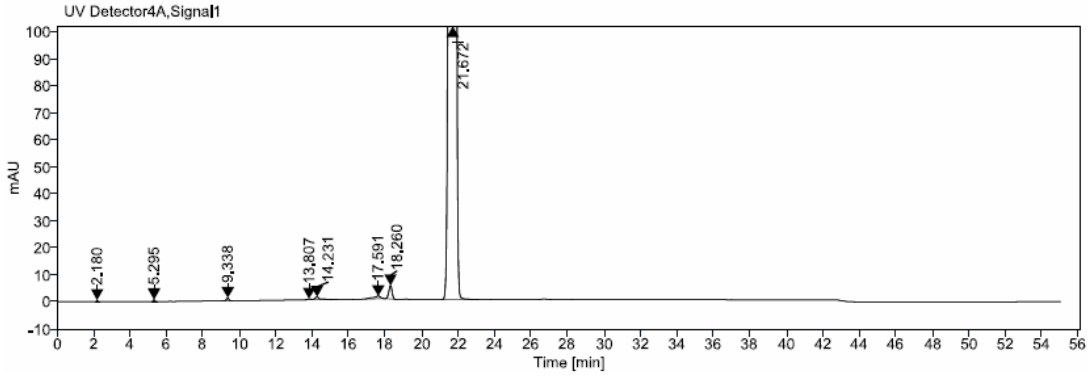

Signal: UV Detector4A,Signal1 Wavelength Ch1 225 nm

Peak Number	RT [min]	Area	Area%	Height	Theoretical Plates	Tail	Resolution
1	2.180	2.25	0.01	0.67	9691	1.20	
2	5.295	4.16	0.01	0.72	19001	1.09	25.72
3	9.338	9.02	0.03	1.07	28600	1.01	21.59
4	13.807	2.66	0.01	0.28	46965	1.16	18.79
5	14.231	13.62	0.05	0.90	32795	1.53	1.49
6	17.591	28.78	0.10	1.04	15782	0.78	7.68
7	18.260	61.11	0.20	5.03	49909	0.94	1.51
8	21.672	30086.70	99.60	1850.24	39864	0.93	8.96
	Sum	30208.31	100.00	1859.96			

SI-22. HPLC spectrum of I-2

琥珀酸去甲文拉法辛分析报告

**Instrument:**	ShimadzuLC08	**Project Name:**	Technology Research
**Manual Modified:**	None	**Operator:**	wujialing
**Sample name:**	intermediate Ⅰ product (O-WLFX-20210427-1-2)	**Inj. volume:**	10
**Location:**	1:7	**Last Modified By:**	wujialing
**Acq. method:**	Related substance method-S08.amx	**Injection Date:**	2021-04-27 17:34:34+08:00
**Acq.Method Version:**	2021-0427-0157-15683	**Modified Date:**	2021-04-28 08:31:05+08:00
**Pro.Method:**	*Related Substance.pmx	**Printied Date:**	2021-04-28 08:36:22+08:00
**Pro.Method Version:**	2021-0428-0030-31077	**Result Set Version:**	2021-0428-0031-17236

Work station:: Agilent OpenLAB CDS Software Version:2.3(Build 2.3.0.468)

Result Path: /Desvenlafaxine Succinate/Technology Research/Results/Related Substance/Intermediate Ⅰ/20210427-intermediate Ⅰ purity detection (synthesis process optimization).rslt

Data file: intermediate Ⅰ product (O-WLFX-20210427-1-2).dx

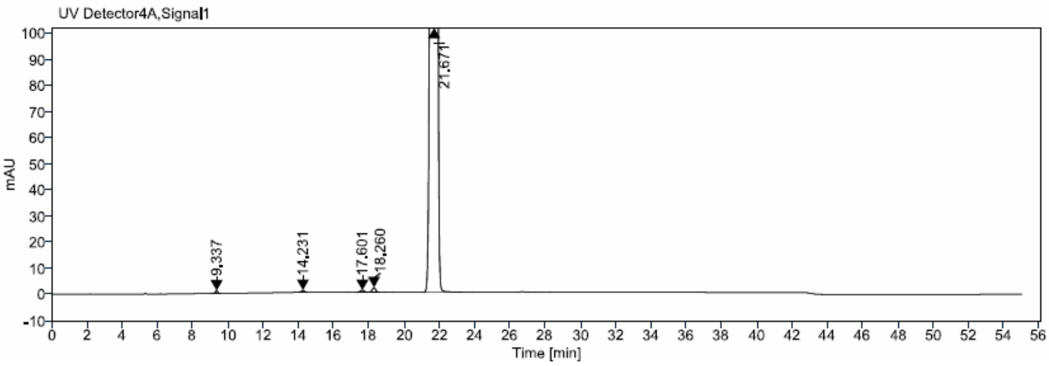

Signal: UV Detector4A,Signal1 Wavelength Ch1 225 nm

Peak Number	RT [min]	Area	Area%	Height	Theoretical Plates	Tail	Resolution
1	9.337	9.42	0.03	1.12	28546	1.03	
2	14.231	8.92	0.03	0.83	38840	1.06	19.20
3	17.601	9.70	0.03	0.76	43346	1.01	10.74
4	18.260	21.15	0.07	1.71	48569	0.96	1.97
5	21.671	29677.97	99.83	1825.89	39874	0.93	8.91
	Sum	29727.17	100.00	1830.31			

SI-23. HPLC spectrum of I-3

琥珀酸去甲文拉法辛分析报告

**Instrument:**	ShimadzuLC08	**Project Name:**	Technology Research
**Manual Modified:**	None	**Operator:**	wujialing
**Sample name:**	intermediate Ⅰ product (O-WLFX-20210427-1-3)	**Inj. volume:**	10
**Location:**	1:8	**Last Modified By:**	wujialing
**Acq. method:**	Related substance method-S08.amx	**Injection Date:**	2021-04-27 18:30:09+08:00
**Acq.Method Version:**	2021-0427-0157-15683	**Modified Date:**	2021-04-28 08:31:05+08:00
**Pro.Method:**	*Related Substance.pmx	**Printied Date:**	2021-04-28 08:36:39+08:00
**Pro.Method Version:**	2021-0428-0030-31077	**Result Set Version:**	2021-0428-0031-17236

Work station:: Agilent OpenLAB CDS Software Version:2.3(Build 2.3.0.468)

Result Path: /Desvenlafaxine Succinate/Technology Research/Results/Related Substance/Intermediate Ⅰ/20210427-intermediate Ⅰ purity detection (synthesis process optimization).rslt

Data file: intermediate Ⅰ product (O-WLFX-20210427-1-3).dx

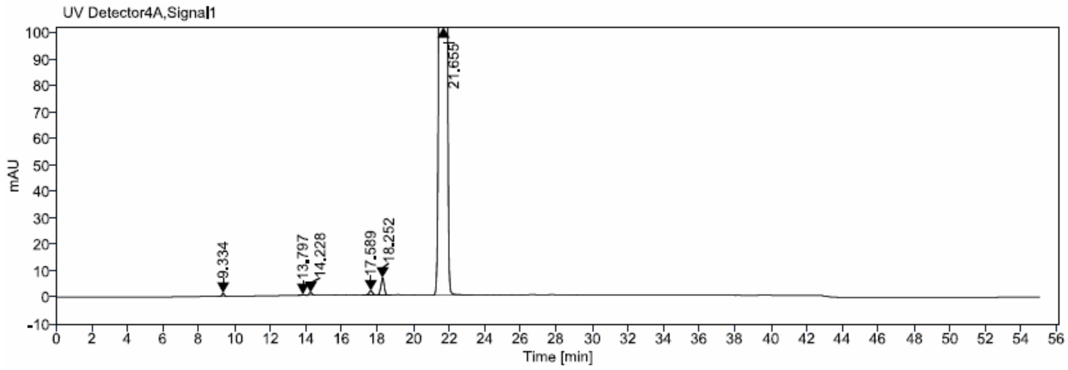

Signal: UV Detector4A,Signal1 Wavelength Ch1 225 nm

Peak Number	RT [min]	Area	Area%	Height	Theoretical Plates	Tail	Resolution
1	9.334	10.31	0.03	1.23	28695	1.02	
2	13.797	2.91	0.01	0.30	45641	1.17	18.65
3	14.228	10.54	0.03	0.99	39915	1.05	1.59
4	17.589	20.07	0.07	1.58	43716	1.00	10.82
5	18.252	79.64	0.26	6.44	48868	0.98	1.98
6	21.655	30605.64	99.60	1883.41	39880	0.93	8.91
	Sum	30729.11	100.00	1893.96			

SI-24. HPLC spectrum of I-4

琥珀酸去甲文拉法辛分析报告

**Instrument:**	ShimadzuLC08	**Project Name:**	Technology Research
**Manual Modified:**	None	**Operator:**	wujialing
**Sample name:**	intermediate Ⅰ (O-WLFX-20210428-1-02-1)	**Inj. volume:**	10
**Location:**	1:2	**Last Modified By:**	wujialing
**Acq. method:**	Related substance method-S08.amx	**Injection Date:**	2021-04-28 10:55:41+08:00
**Acq.Method Version:**	2021-0427-0157-15683	**Modified Date:**	2021-04-29 08:39:20+08:00
**Pro.Method:**	*Related Substance.pmx	**Printied Date:**	2021-04-29 08:41:02+08:00
**Pro.Method Version:**	2021-0429-0039-20616	**Result Set Version:**	2021-0429-0039-31362

Work station:: Agilent OpenLAB CDS Software Version:2.3(Build 2.3.0.468)

Result Path: /Desvenlafaxine Succinate/Technology Research/Results/Related Substance/Intermediate Ⅰ/20210428-intermediate Ⅰ purity detection (synthesis process optimization).rslt

Data file: intermediate Ⅰ (O-WLFX-20210428-1-02-1).dx

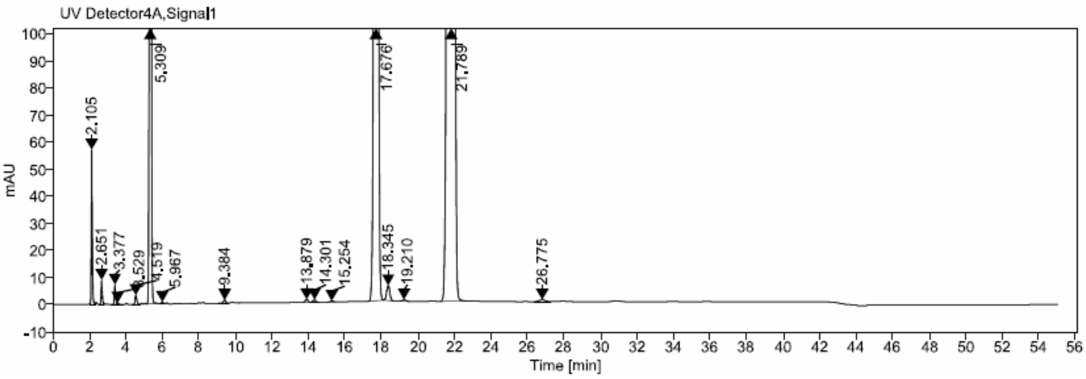

Signal: UV Detector4A,Signal1 Wavelength Ch1 225 nm

Peak Number	RT [min]	Area	Area%	Height	Theoretical Plates	Tail	Resolution
1	2.105	216.16	0.51	57.28	7706	1.47	
2	2.651	35.92	0.08	9.10	10891	1.27	5.53
3	3.377	31.98	0.08	7.43	14678	1.22	6.80
4	3.529	4.32	0.01	0.73	8789	2.76	1.16
5	4.519	20.16	0.05	3.38	14141	1.29	6.54
6	5.309	2996.51	7.03	521.63	19422	1.06	5.19
7	5.967	4.58	0.01	0.61	14303	0.92	3.74
8	9.384	10.12	0.02	1.19	28079	1.06	16.13
9	13.879	10.28	0.02	1.07	45372	1.02	18.56
10	14.301	9.11	0.02	0.86	40316	0.98	1.54

琥珀酸去甲文拉法辛分析报告

Peak Number	RT [min]	Area	Area%	Height	Theoretical Plates	Tail	Resolution
11	15.254	4.35	0.01	0.40	43956	1.22	3.31
12	17.676	7005.88	16.44	558.25	44393	0.98	7.73
13	18.345	67.49	0.16	5.57	50348	0.94	2.02
14	19.210	6.82	0.02	0.52	47156	0.87	2.54
15	21.789	32167.15	75.50	1951.22	39326	0.92	6.50
16	26.775	14.12	0.03	0.63	31772	1.01	9.59
	Sum	42604.94	100.00	3119.88			

SI-25. HPLC spectrum of I-5

琥珀酸去甲文拉法辛分析报告

**Instrument:**	ShimadzuLC08	**Project Name:**	Technology Research
**Manual Modified:**	None	**Operator:**	wujialing
**Sample name:**	intermediate Ⅰ(O-WLFX-20210428-1-02-2)	**Inj. volume:**	10
**Location:**	1:4	**Last Modified By:**	wujialing
**Acq. method:**	Related substance method-S08.amx	**Injection Date:**	2021-04-28 13:16:12+08:00
**Acq.Method Version:**	2021-0427-0157-15683	**Modified Date:**	2021-04-29 08:39:20+08:00
**Pro.Method:**	*Related Substance.pmx	**Printied Date:**	2021-04-29 08:42:04+08:00
**Pro.Method Version:**	2021-0429-0039-20616	**Result Set Version:**	2021-0429-0039-31362

Work station:: Agilent OpenLAB CDS Software Version:2.3(Build 2.3.0.468)

Result Path: /Desvenlafaxine Succinate/Technology Research/Results/Related Substance/Intermediate Ⅰ/20210428-intermediate Ⅰ purity detection (synthesis process optimization).rslt

Data file: intermediate Ⅰ(O-WLFX-20210428-1-02-2).dx

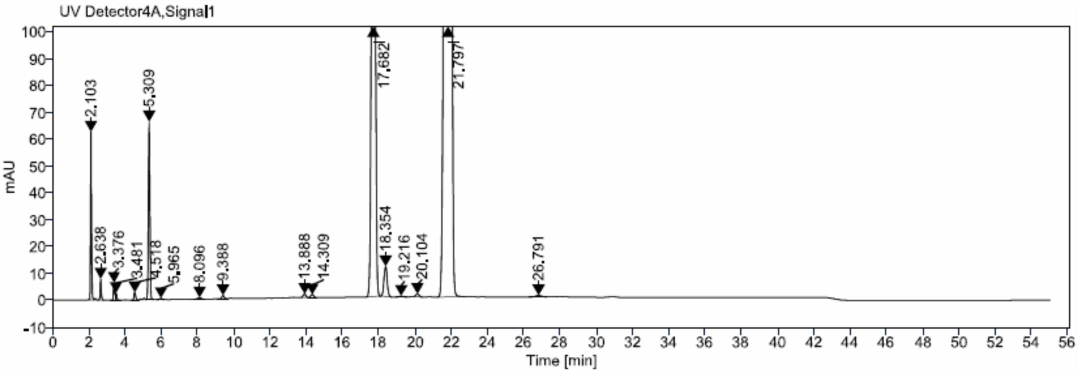

Signal: UV Detector4A,Signal1 Wavelength Ch1 225 nm

Peak Number	RT [min]	Area	Area%	Height	Theoretical Plates	Tail	Resolution
1	2.103	229.34	0.66	62.75	7795	1.35	
2	2.638	31.91	0.09	8.01	11660	1.16	5.54
3	3.376	28.36	0.08	6.51	13351	0.99	6.88
4	3.481	15.08	0.04	2.74	5129	2.35	0.67
5	4.518	16.14	0.05	2.83	14829	1.17	6.05
6	5.309	382.32	1.09	66.35	19268	1.05	5.25
7	5.965	3.82	0.01	0.50	14086	0.94	3.70
8	8.096	7.01	0.02	0.57	9846	1.04	8.08
9	9.388	10.74	0.03	1.26	27843	1.10	4.68
10	13.888	12.09	0.03	1.25	44803	0.97	18.46

琥珀酸去甲文拉法辛分析报告

Peak Number	RT [min]	Area	Area%	Height	Theoretical Plates	Tail	Resolution
11	14.309	11.96	0.03	1.13	40718	0.94	1.54
12	17.682	3609.59	10.31	287.89	44488	0.99	10.90
13	18.354	138.80	0.40	11.27	49246	0.98	2.02
14	19.216	5.65	0.02	0.43	47087	0.97	2.52
15	20.104	13.47	0.04	1.04	51775	0.89	2.51
16	21.797	30482.04	87.06	1859.21	39692	0.93	4.28
17	26.791	12.82	0.04	0.59	32993	1.04	9.72
	Sum	35011.14	100.00	2314.32			

SI-26. HPLC spectrum of I-6

琥珀酸去甲文拉法辛分析报告

Instrument: ShimadzuLC08

Project Name: Technology Research

Manual Modified: None

Operator: wujialing

Sample name: intermediate Ⅰ(O-WLFX-20210428-1-02-3)

Inj. volume: 10

Location: 1:6

Last Modified By: wujialing

Acq. method: Related substance method-S08.amx

Injection Date: 2021-04-28 15:07:21+08:00

Acq.Method Version: 2021-0427-0157-15683

Modified Date: 2021-04-29 08:39:20+08:00

Pro.Method: *Related Substance.pmx

Printied Date: 2021-04-29 08:42:49+08:00

Pro.Method Version: 2021-0429-0039-20616

Result Set Version: 2021-0429-0039-31362

Work station:: Agilent OpenLAB CDS Software Version:2.3(Build 2.3.0.468)

Result Path: /Desvenlafaxine Succinate/Technology Research/Results/Related Substance/Intermediate Ⅰ/20210428-intermediate Ⅰ purity detection (synthesis process optimization).rslt

Data file: intermediate Ⅰ(O-WLFX-20210428-1-02-3).dx

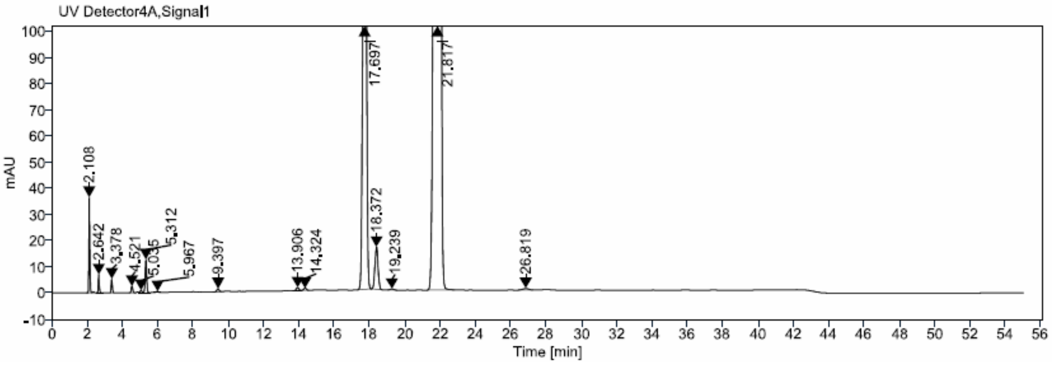

Signal: UV Detector4A,Signal1 Wavelength Ch1 225 nm

Peak Number	RT [min]	Area	Area%	Height	Theoretical Plates	Tail	Resolution
1	2.108	130.30	0.41	36.58	8181	1.31	
2	2.642	26.75	0.08	7.09	11937	1.29	5.62
3	3.378	22.56	0.07	5.42	14953	1.18	7.11
4	4.521	14.41	0.05	2.61	15384	1.12	8.91
5	5.035	5.08	0.02	0.95	19995	1.11	3.57
6	5.312	73.19	0.23	12.74	19350	1.06	1.87
7	5.967	3.43	0.01	0.46	14213	0.94	3.71
8	9.397	10.67	0.03	1.23	27627	1.08	16.09
9	13.906	11.74	0.04	1.21	44940	1.08	18.46
10	14.324	8.63	0.03	0.84	42254	1.20	1.55

琥珀酸去甲文拉法辛分析报告

Peak Number	RT [min]	Area	Area%	Height	Theoretical Plates	Tail	Resolution
11	17.697	2905.55	9.16	231.63	44522	1.00	10.98
12	18.372	200.75	0.63	16.22	49015	0.98	2.02
13	19.239	5.03	0.02	0.39	49045	0.92	2.56
14	21.817	28295.16	89.19	1733.63	40034	0.93	6.58
15	26.819	11.98	0.04	0.55	33033	1.01	9.75
	Sum	31725.21	100.00	2051.55			

SI-27. HPLC spectrum of I-7

琥珀酸去甲文拉法辛分析报告

**Instrument:**	ShimadzuLC08	**Project Name:**	Technology Research
**Manual Modified:**	None	**Operator:**	wujialing
**Sample name:**	intermediate Ⅰ (O-WLFX-20210429-1-1)	**Inj. volume:**	10
**Location:**	1:2	**Last Modified By:**	wujialing
**Acq. method:**	Related substance method-S08.amx	**Injection Date:**	2021-04-29 17:02:56+08:00
**Acq.Method Version:**	2021-0427-0157-15683	**Modified Date:**	2021-04-30 08:07:42+08:00
**Pro.Method:**	*Related Substance.pmx	**Printied Date:**	2021-04-30 08:11:01+08:00
**Pro.Method Version:**	2021-0430-0003-37414	**Result Set Version:**	2021-0430-0009-17274

Work station:: Agilent OpenLAB CDS Software Version:2.3(Build 2.3.0.468)

Result Path: /Desvenlafaxine Succinate/Technology Research/Results/Related Substance/Intermediate Ⅰ/20210429-intermediate Ⅰ purity detection (synthesis process optimization).rslt

Data file: intermediate Ⅰ (O-WLFX-20210429-1-1).dx

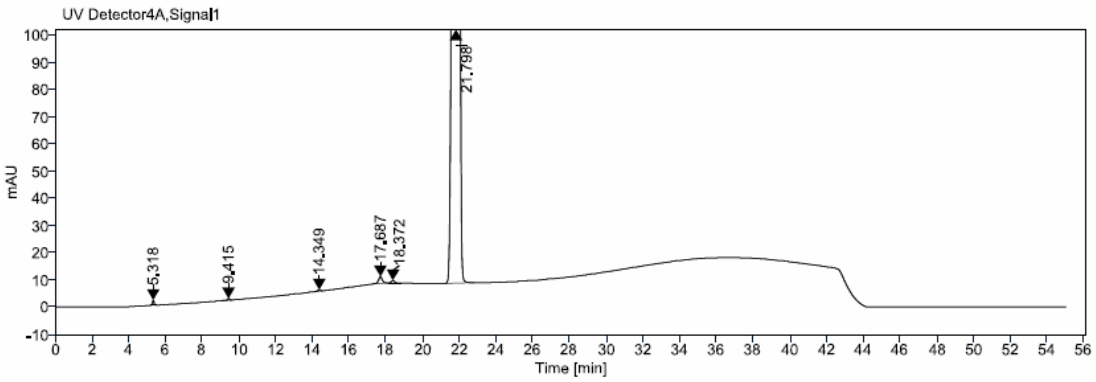

Signal: UV Detector4A,Signal1 Wavelength Ch1 225 nm

Peak Number	RT [min]	Area	Area%	Height	Theoretical Plates	Tail	Resolution
1	5.318	10.81	0.03	1.90	19726	1.13	
2	9.415	5.14	0.02	0.63	30168	1.22	22.25
3	14.349	5.20	0.02	0.51	43313	1.17	20.04
4	17.687	34.16	0.11	2.49	40780	1.42	10.66
5	18.372	10.60	0.03	0.86	48951	0.84	2.01
6	21.798	31472.94	99.79	1924.79	39944	0.93	8.92
	Sum	31538.86	100.00	1931.18			

SI-28. HPLC spectrum of I-8

琥珀酸去甲文拉法辛分析报告

**Instrument:**	ShimadzuLC08	**Project Name:**	Technology Research
**Manual Modified:**	None	**Operator:**	wujialing
**Sample name:**	intermediate Ⅰ (O-WLFX-20210429-1-2)	**Inj. volume:**	10
**Location:**	1:3	**Last Modified By:**	wujialing
**Acq. method:**	Related substance method-S08.amx	**Injection Date:**	2021-04-29 17:58:31+08:00
**Acq.Method Version:**	2021-0427-0157-15683	**Modified Date:**	2021-04-30 08:07:42+08:00
**Pro.Method:**	*Related Substance.pmx	**Printied Date:**	2021-04-30 08:12:07+08:00
**Pro.Method Version:**	2021-0430-0003-37414	**Result Set Version:**	2021-0430-0009-17274

Work station:: Agilent OpenLAB CDS Software Version:2.3(Build 2.3.0.468)

Result Path: /Desvenlafaxine Succinate/Technology Research/Results/Related Substance/Intermediate Ⅰ/20210429-intermediate Ⅰ purity detection (synthesis process optimization).rslt

Data file: intermediate Ⅰ (O-WLFX-20210429-1-2).dx

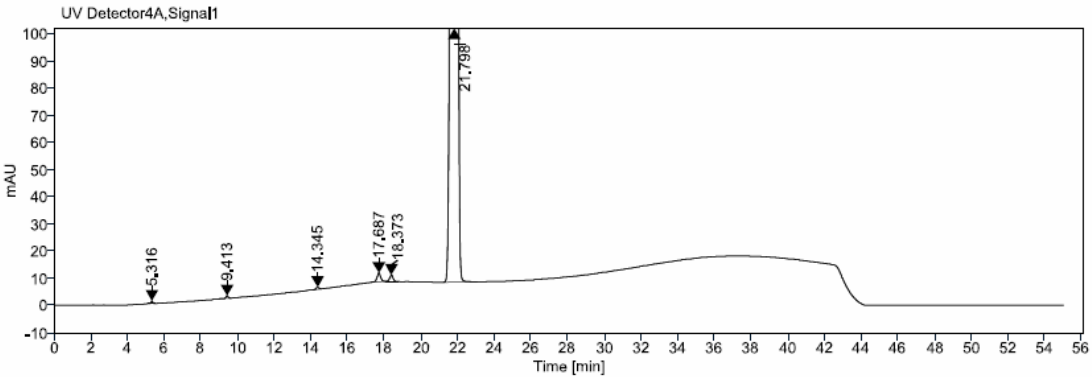

Signal: UV Detector4A,Signal1 Wavelength Ch1 225 nm

Peak Number	RT [min]	Area	Area%	Height	Theoretical Plates	Tail	Resolution
1	5.316	5.83	0.02	1.03	19849	1.34	
2	9.413	8.73	0.03	1.04	28855	1.20	21.99
3	14.345	7.85	0.03	0.75	42083	1.16	19.67
4	17.687	43.44	0.14	3.23	41560	1.45	10.66
5	18.373	29.11	0.10	2.35	49260	0.92	2.03
6	21.798	30060.85	99.69	1846.13	40205	0.93	8.94
	Sum	30155.80	100.00	1854.52			

SI-29. HPLC spectrum of I-9

琥珀酸去甲文拉法辛分析报告

**Instrument:**	ShimadzuLC08	**Project Name:**	Technology Research
**Manual Modified:**	None	**Operator:**	wujialing
**Sample name:**	intermediate Ⅰ (O-WLFX-20210429-1-3)	**Inj. volume:**	10
**Location:**	1:4	**Last Modified By:**	wujialing
**Acq. method:**	Related substance method-S08.amx	**Injection Date:**	2021-04-29 18:54:06+08:00
**Acq.Method Version:**	2021-0427-0157-15683	**Modified Date:**	2021-04-30 08:07:42+08:00
**Pro.Method:**	*Related Substance.pmx	**Printied Date:**	2021-04-30 08:12:24+08:00
**Pro.Method Version:**	2021-0430-0003-37414	**Result Set Version:**	2021-0430-0009-17274

Work station:: Agilent OpenLAB CDS Software Version:2.3(Build 2.3.0.468)

Result Path: /Desvenlafaxine Succinate/Technology Research/Results/Related Substance/Intermediate Ⅰ/20210429-intermediate Ⅰ purity detection (synthesis process optimization).rslt

Data file: intermediate Ⅰ (O-WLFX-20210429-1-3).dx

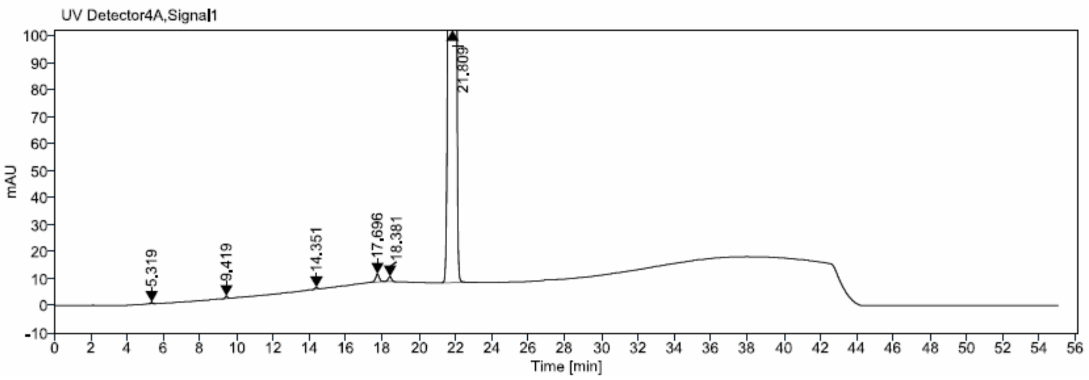

Signal: UV Detector4A,Signal1 Wavelength Ch1 225 nm

Peak Number	RT [min]	Area	Area%	Height	Theoretical Plates	Tail	Resolution
1	5.319	3.95	0.01	0.70	20128	1.39	
2	9.419	7.76	0.03	0.94	29148	1.22	22.13
3	14.351	7.05	0.02	0.68	42386	1.16	19.75
4	17.696	40.63	0.14	2.99	41110	1.46	10.65
5	18.381	23.80	0.08	1.92	49273	0.88	2.02
6	21.809	28398.54	99.71	1748.81	40401	0.93	8.96
	Sum	28481.74	100.00	1756.04			

SI-30. HPLC spectrum of I-10

琥珀酸去甲文拉法辛分析报告

**Instrument:**	ShimadzuLC08	**Project Name:**	Technology Research
**Manual Modified:**	None	**Operator:**	wujialing
**Sample name:**	intermediate Ⅰ (O-WLFX-20210429-1-4)	**Inj. volume:**	10
**Location:**	1:5	**Last Modified By:**	wujialing
**Acq. method:**	Related substance method-S08.amx	**Injection Date:**	2021-04-29 19:49:41+08:00
**Acq.Method Version:**	2021-0427-0157-15683	**Modified Date:**	2021-04-30 08:07:42+08:00
**Pro.Method:**	*Related Substance.pmx	**Printied Date:**	2021-04-30 08:12:41+08:00
**Pro.Method Version:**	2021-0430-0003-37414	**Result Set Version:**	2021-0430-0009-17274

Work station:: Agilent OpenLAB CDS Software Version:2.3(Build 2.3.0.468)

Result Path: /Desvenlafaxine Succinate/Technology Research/Results/Related Substance/Intermediate Ⅰ/20210429-intermediate Ⅰ purity detection (synthesis process optimization).rslt

Data file: intermediate Ⅰ (O-WLFX-20210429-1-4).dx

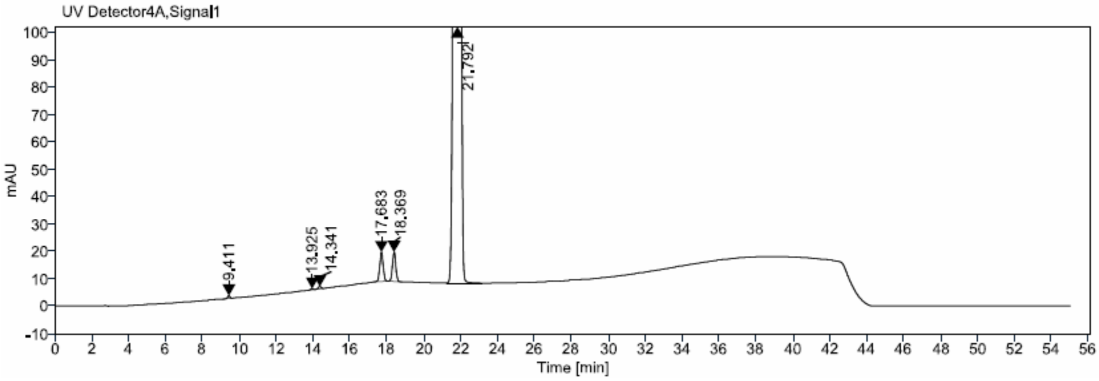

Signal: UV Detector4A,Signal1 Wavelength Ch1 225 nm

Peak Number	RT [min]	Area	Area%	Height	Theoretical Plates	Tail	Resolution
1	9.411	8.80	0.03	1.06	28903	1.23	
2	13.925	3.85	0.01	0.41	47314	1.17	18.91
3	14.341	8.60	0.03	0.83	42258	1.18	1.56
4	17.683	137.22	0.50	10.77	43947	1.03	10.84
5	18.369	134.18	0.49	10.79	48865	0.97	2.05
6	21.792	27092.37	98.93	1672.43	40489	0.94	8.94
	Sum	27385.02	100.00	1696.29			

SI-31. HPLC spectrum of I-11

琥珀酸去甲文拉法辛分析报告

**Instrument:**	ShimadzuLC08	**Project Name:**	Technology Research
**Manual Modified:**	None	**Operator:**	wujialing
**Sample name:**	intermediate Ⅰ (O-WLFX-20210430-1-1)	**Inj. volume:**	10
**Location:**	1:2	**Last Modified By:**	wujialing
**Acq. method:**	Related substance method-S08.amx	**Injection Date:**	2021-04-30 14:09:50+08:00
**Acq.Method Version:**	2021-0427-0157-15683	**Modified Date:**	2021-04-30 17:02:30+08:00
**Pro.Method:**	*Related Substance.pmx	**Printied Date:**	2021-04-30 17:04:33+08:00
**Pro.Method Version:**	2021-0430-0902-30622	**Result Set Version:**	2021-0430-0902-36795

Work station:: Agilent OpenLAB CDS Software Version:2.3(Build 2.3.0.468)

Result Path: /Desvenlafaxine Succinate/Technology Research/Results/Related Substance/Intermediate Ⅰ/20210430-intermediate Ⅰ purity detection (synthesis process optimization).rslt

Data file: intermediate Ⅰ (O-WLFX-20210430-1-1).dx

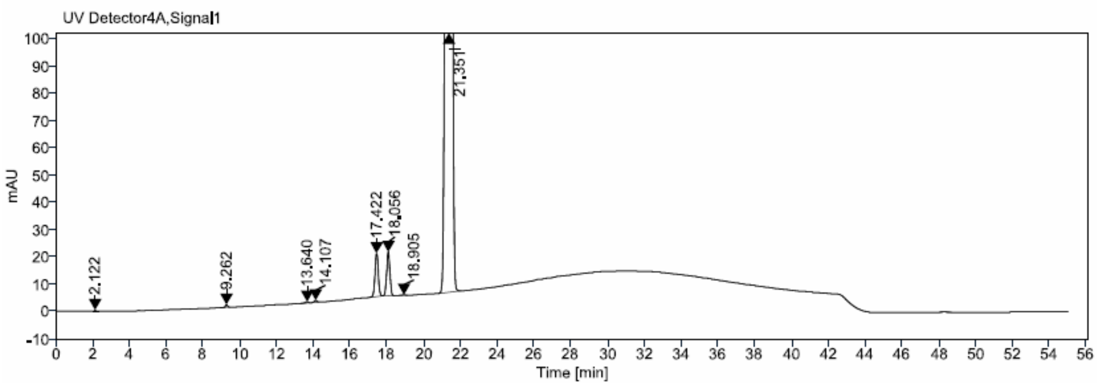

Signal: UV Detector4A,Signal1 Wavelength Ch1 225 nm

Peak Number	RT [min]	Area	Area%	Height	Theoretical Plates	Tail	Resolution
1	2.122	2.01	0.01	0.53	8920	1.65	
2	9.262	8.11	0.04	0.98	28609	1.20	46.23
3	13.640	4.65	0.02	0.48	44431	1.21	18.32
4	14.107	7.61	0.03	0.74	41235	1.18	1.75
5	17.422	194.13	0.84	15.78	44729	1.04	10.91
6	18.056	193.81	0.84	16.07	50037	1.00	1.95
7	18.905	1.93	0.01	0.16	53994	1.15	2.62
8	21.351	22720.89	98.22	1459.38	42263	0.95	6.60
	Sum	23133.14	100.00	1494.13			

SI-32. HPLC spectrum of I-12

琥珀酸去甲文拉法辛分析报告

**Instrument:**	ShimadzuLC08	**Project Name:**	Technology Research
**Manual Modified:**	None	**Operator:**	wujialing
**Sample name:**	intermediate Ⅰ (O-WLFX-20210430-1-2)	**Inj. volume:**	10
**Location:**	1:3	**Last Modified By:**	wujialing
**Acq. method:**	Related substance method-S08.amx	**Injection Date:**	2021-04-30 15:05:25+08:00
**Acq.Method Version:**	2021-0427-0157-15683	**Modified Date:**	2021-04-30 17:02:30+08:00
**Pro.Method:**	*Related Substance.pmx	**Printied Date:**	2021-04-30 17:05:43+08:00
**Pro.Method Version:**	2021-0430-0902-30622	**Result Set Version:**	2021-0430-0902-36795
**Work station::**	Agilent OpenLAB CDS Software Version:2.3(Build 2.3.0.468)		
**Result Path:**	/Desvenlafaxine Succinate/Technology Research/Results/Related Substance/Intermediate Ⅰ/20210430-intermediate Ⅰ purity detection (synthesis process optimization).rslt		
**Data file:**	intermediate Ⅰ (O-WLFX-20210430-1-2).dx		

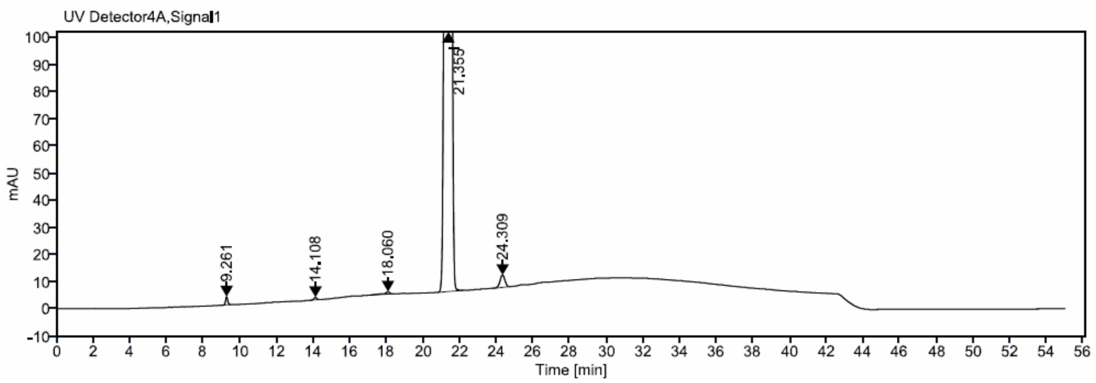

Signal: UV Detector4A,Signal1 Wavelength Ch1 225 nm

Peak Number	RT [min]	Area	Area%	Height	Theoretical Plates	Tail	Resolution
1	9.261	25.33	0.09	3.07	28395	1.06	
2	14.108	10.32	0.04	0.92	35988	1.17	18.74
3	18.060	18.16	0.06	0.84	40108	0.90	12.01
4	21.355	28818.32	99.53	1830.61	41591	0.93	8.45
5	24.309	83.13	0.29	4.53	39186	1.23	6.49
	Sum	28955.25	100.00	1839.97			

SI-33. HPLC spectrum of I-13

No solids precipitated

SI-34. HPLC spectrum of I-14

琥珀酸去甲文拉法辛分析报告

**Instrument:**	ShimadzuLC08	**Project Name:**	Technology Research
**Manual Modified:**	None	**Operator:**	wujialing
**Sample name:**	intermediate Ⅰ (O-WLFX-20210430-1-4)	**Inj. volume:**	10
**Location:**	1:4	**Last Modified By:**	wujialing
**Acq. method:**	Related substance method-S08.amx	**Injection Date:**	2021-04-30 17:42:51+08:00
**Acq.Method Version:**	2021-0427-0157-15683	**Modified Date:**	2021-05-06 08:35:01+08:00
**Pro.Method:**	*Related Substance.pmx	**Printied Date:**	2021-05-06 08:37:31+08:00
**Pro.Method Version:**	2021-0506-0033-06533	**Result Set Version:**	2021-0506-0036-13803

Work station:: Agilent OpenLAB CDS Software Version:2.3(Build 2.3.0.468)

Result Path: /Desvenlafaxine Succinate/Technology Research/Results/Related Substance/Intermediate Ⅰ/20210430-intermediate Ⅰ purity detection (synthesis process optimization)-1.rslt

Data file: intermediate Ⅰ (O-WLFX-20210430-1-4).dx

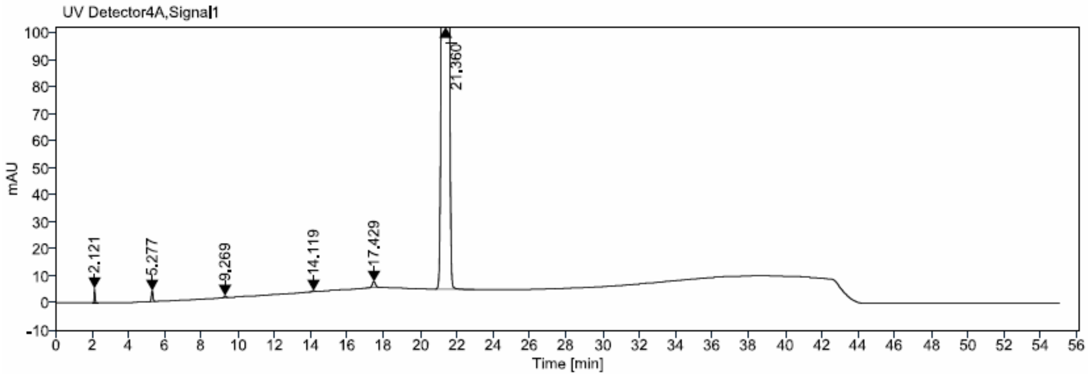

Signal: UV Detector4A,Signal1 Wavelength Ch1 225 nm

Peak Number	RT [min]	Area	Area%	Height	Theoretical Plates	Tail	Resolution
1	2.121	18.80	0.08	5.42	8807	1.32	
2	5.277	23.21	0.10	4.05	19317	1.10	26.04
3	9.269	5.89	0.03	0.72	28888	1.26	21.58
4	14.119	4.48	0.02	0.42	38901	1.16	19.23
5	17.429	30.03	0.13	2.28	42383	1.64	10.59
6	21.360	22565.63	99.64	1449.59	42342	0.95	10.43
	Sum	22648.05	100.00	1462.48			

SI-35. HPLC spectrum of I-15

琥珀酸去甲文拉法辛分析报告

**Instrument:**	ShimadzuLC08	**Project Name:**	Technology Research
**Manual Modified:**	None	**Operator:**	wujialing
**Sample name:**	intermediate Ⅰ (O-WLFX-20210507-1-1)	**Inj. volume:**	10
**Location:**	1:2	**Last Modified By:**	wujialing
**Acq. method:**	Related substance method-S08.amx	**Injection Date:**	2021-05-07 19:20:58+08:00
**Acq.Method Version:**	2021-0427-0157-15683	**Modified Date:**	2021-05-08 08:06:33+08:00
**Pro.Method:**	*Related Substance.pmx	**Printied Date:**	2021-05-08 08:30:33+08:00
**Pro.Method Version:**	2021-0508-0006-20663	**Result Set Version:**	2021-0508-0007-12516

Work station:: Agilent OpenLAB CDS Software Version:2.3(Build 2.3.0.468)

Result Path: /Desvenlafaxine Succinate/Technology Research/Results/Related Substance/Intermediate Ⅰ/20210507-intermediate Ⅰ purity detection (synthesis process optimization).rslt

Data file: intermediate Ⅰ (O-WLFX-20210507-1-1).dx

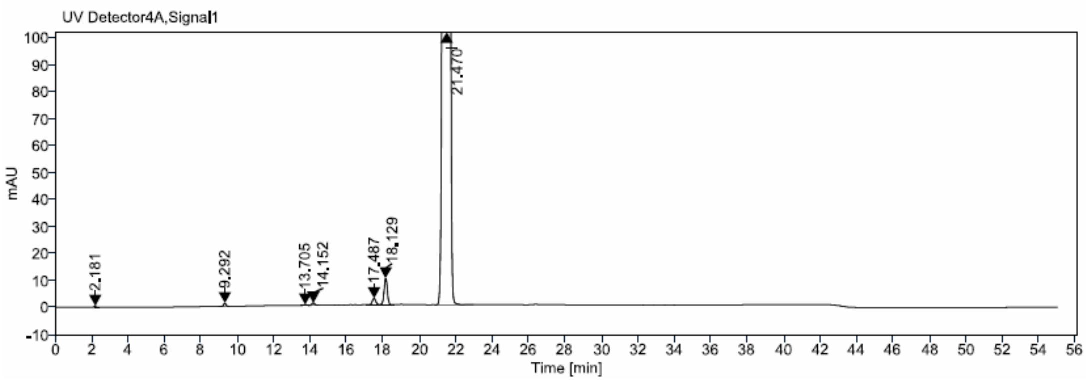

Signal: UV Detector4A,Signal1 Wavelength Ch1 225 nm

Peak Number	RT [min]	Area	Area%	Height	Theoretical Plates	Tail	Resolution
1	2.181	2.42	0.01	0.68	8267	1.37	
2	9.292	10.37	0.03	1.23	27993	1.04	44.70
3	13.705	3.71	0.01	0.39	44265	1.08	18.29
4	14.152	9.94	0.03	0.94	40233	1.03	1.65
5	17.487	29.52	0.10	2.39	44670	1.03	10.88
6	18.129	118.64	0.40	9.74	49588	0.99	1.95
7	21.470	29825.21	99.42	1866.31	40913	0.93	8.91
	Sum	29999.80	100.00	1881.68			

SI-36. HPLC spectrum of I-16

琥珀酸去甲文拉法辛分析报告

**Instrument:**	ShimadzuLC08	**Project Name:**	Technology Research
**Manual Modified:**	None	**Operator:**	wujialing
**Sample name:**	intermediate Ⅰ (O-WLFX-20210507-1-2)	**Inj. volume:**	10
**Location:**	1:3	**Last Modified By:**	wujialing
**Acq. method:**	Related substance method-S08.amx	**Injection Date:**	2021-05-07 20:16:31+08:00
**Acq.Method Version:**	2021-0427-0157-15683	**Modified Date:**	2021-05-08 08:06:33+08:00
**Pro.Method:**	*Related Substance.pmx	**Printied Date:**	2021-05-08 08:31:55+08:00
**Pro.Method Version:**	2021-0508-0006-20663	**Result Set Version:**	2021-0508-0007-12516

Work station:: Agilent OpenLAB CDS Software Version:2.3(Build 2.3.0.468)

Result Path: /Desvenlafaxine Succinate/Technology Research/Results/Related Substance/Intermediate Ⅰ/20210507-intermediate Ⅰ purity detection (synthesis process optimization).rslt

Data file: intermediate Ⅰ (O-WLFX-20210507-1-2).dx

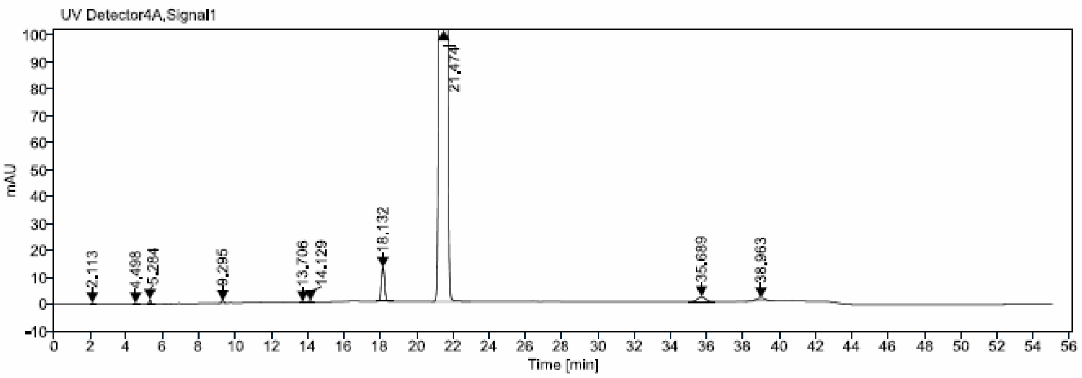

Signal: UV Detector4A,Signal1 Wavelength Ch1 225 nm

Peak Number	RT [min]	Area	Area%	Height	Theoretical Plates	Tail	Resolution
1	2.113	1.94	0.01	0.52	9230	1.69	
2	4.498	2.09	0.01	0.30	10740	1.76	18.23
3	5.284	8.96	0.03	1.56	19259	1.08	4.83
4	9.295	7.03	0.02	0.84	28159	1.06	21.45
5	13.706	1.57	0.01	0.17	47151	0.93	18.61
6	14.129	3.28	0.01	0.26	28459	0.96	1.44
7	18.132	153.33	0.54	12.55	49461	0.99	12.11
8	21.474	28341.99	99.04	1779.13	41110	0.93	8.92
9	35.689	54.04	0.19	1.78	31197	0.98	23.08
10	38.963	41.32	0.14	1.19	28511	1.21	3.78
	Sum	28615.56	100.00	1798.32			

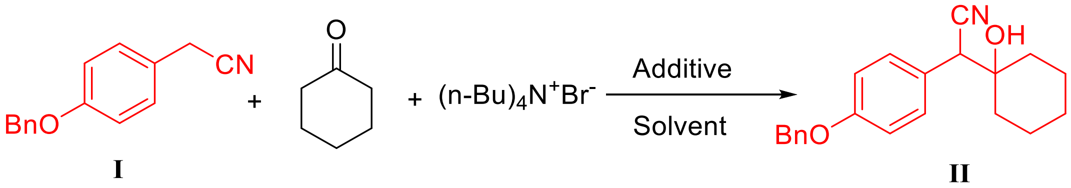

SI-37. HPLC spectrum of II-1

琥珀酸去甲文拉法辛分析报告

**Instrument:**	ShimadzuLC08	**Project Name:**	Technology Research
**Manual Modified:**	None	**Operator:**	wujialing
**Sample name:**	intermediate Ⅱ(O-WLFX-20210517-2-1)	**Inj. volume:**	10
**Location:**	1:3	**Last Modified By:**	wujialing
**Acq. method:**	Related substance method-S08.amx	**Injection Date:**	2021-05-18 12:13:39+08:00
**Acq.Method Version:**	2021-0518-0105-13016	**Modified Date:**	2021-05-18 15:44:26+08:00
**Pro.Method:**	*Related Substance.pmx	**Printied Date:**	2021-05-19 16:10:03+08:00
**Pro.Method Version:**	2021-0518-0740-06018	**Result Set Version:**	2021-0518-0744-39566

Work station:: Agilent OpenLAB CDS Software Version:2.3(Build 2.3.0.468)

Result Path: /Desvenlafaxine Succinate/Technology Research/Results/Related Substance/Intermediate Ⅱ/20210518-intermediate Ⅱ purity detection.rslt

Data file: intermediate Ⅱ(O-WLFX-20210517-2-1).dx

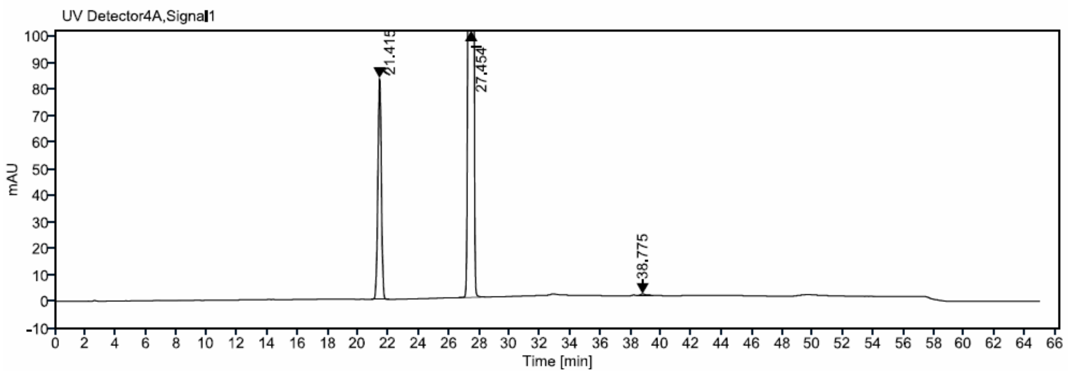

Signal: UV Detector4A,Signal1 Wavelength Ch1 225 nm

Peak Number	RT [min]	Area	Area%	Height	Theoretical Plates	Tail	Resolution
1	21.415	1317.63	5.51	83.41	41246	0.99	
2	27.454	22578.62	94.42	1643.69	90210	0.94	15.34
3	38.775	16.70	0.07	0.70	75408	1.70	24.34
	Sum	23912.96	100.00	1727.80			

SI-38. HPLC spectrum of II-2

琥珀酸去甲文拉法辛分析报告

**Instrument:**	ShimadzuLC08	**Project Name:**	Technology Research
**Manual Modified:**	None	**Operator:**	wujialing
**Sample name:**	intermediate Ⅱ(O-WLFX-20210517-2-2)	**Inj. volume:**	10
**Location:**	1:4	**Last Modified By:**	wujialing
**Acq. method:**	Related substance method-S08.amx	**Injection Date:**	2021-05-18 13:19:12+08:00
**Acq.Method Version:**	2021-0518-0105-13016	**Modified Date:**	2021-05-18 15:44:26+08:00
**Pro.Method:**	*Related Substance.pmx	**Printied Date:**	2021-05-19 16:10:34+08:00
**Pro.Method Version:**	2021-0518-0740-06018	**Result Set Version:**	2021-0518-0744-39566

Work station:: Agilent OpenLAB CDS Software Version:2.3(Build 2.3.0.468)

Result Path: /Desvenlafaxine Succinate/Technology Research/Results/Related Substance/Intermediate Ⅱ/20210518-intermediate Ⅱ purity detection.rslt

Data file: intermediate Ⅱ(O-WLFX-20210517-2-2).dx

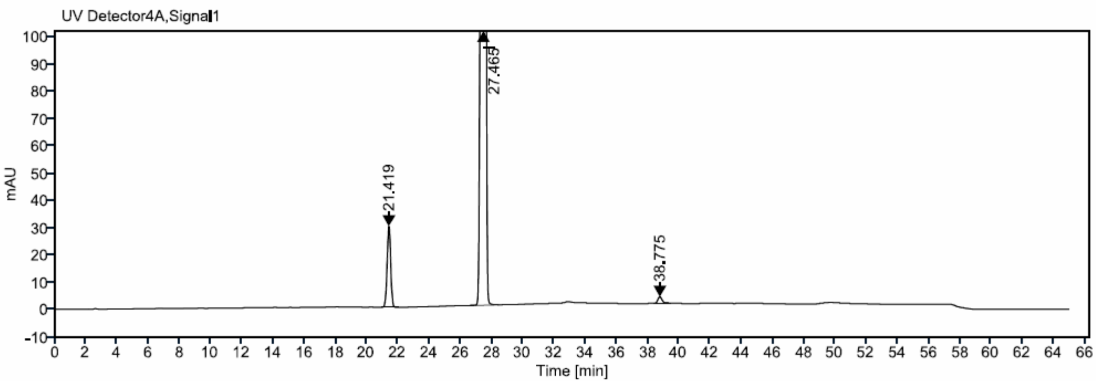

Signal: UV Detector4A,Signal1 Wavelength Ch1 225 nm

Peak Number	RT [min]	Area	Area%	Height	Theoretical Plates	Tail	Resolution
1	21.419	469.47	1.97	29.71	41225	0.99	
2	27.465	23268.62	97.83	1690.59	89971	0.94	15.34
3	38.775	46.90	0.20	2.44	98622	1.46	26.30
	Sum	23784.99	100.00	1722.74			

SI-39. HPLC spectrum of II-3

琥珀酸去甲文拉法辛分析报告

**Instrument:**	ShimadzuLC08	**Project Name:**	Technology Research
**Manual Modified:**	None	**Operator:**	wujialing
**Sample name:**	intermediate Ⅱ(O-WLFX-20210517-2-3)	**Inj. volume:**	10
**Location:**	1:5	**Last Modified By:**	wujialing
**Acq. method:**	Related substance method-S08.amx	**Injection Date:**	2021-05-18 14:24:46+08:00
**Acq.Method Version:**	2021-0518-0105-13016	**Modified Date:**	2021-05-18 15:44:26+08:00
**Pro.Method:**	*Related Substance.pmx	**Printied Date:**	2021-05-19 16:10:54+08:00
**Pro.Method Version:**	2021-0518-0740-06018	**Result Set Version:**	2021-0518-0744-39566

Work station:: Agilent OpenLAB CDS Software Version:2.3(Build 2.3.0.468)

Result Path: /Desvenlafaxine Succinate/Technology Research/Results/Related Substance/Intermediate Ⅱ/20210518-intermediate Ⅱ purity detection.rslt

Data file: intermediate Ⅱ(O-WLFX-20210517-2-3).dx

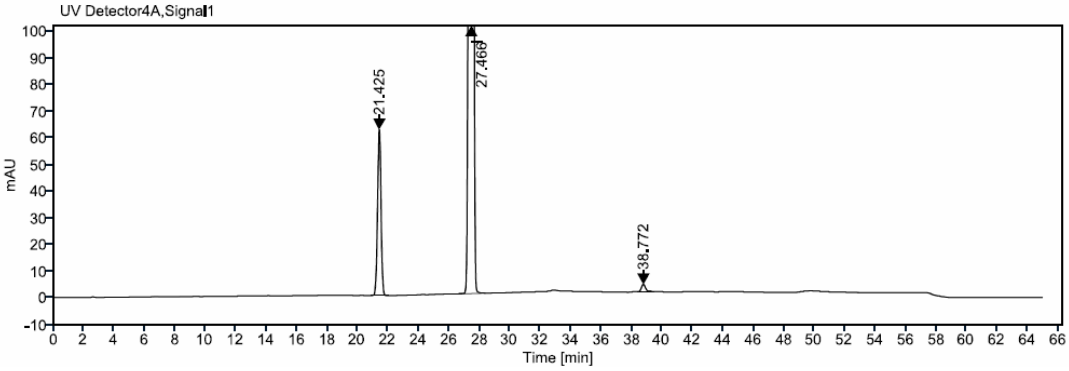

Signal: UV Detector4A,Signal1 Wavelength Ch1 225 nm

Peak Number	RT [min]	Area	Area%	Height	Theoretical Plates	Tail	Resolution
1	21.425	984.74	4.15	62.26	41172	0.99	
2	27.466	22667.20	95.62	1650.94	90361	0.94	15.33
3	38.772	53.99	0.23	2.83	99331	1.40	26.37
	Sum	23705.93	100.00	1716.03			

SI-40. HPLC spectrum of II-4

琥珀酸去甲文拉法辛分析报告

**Instrument:**	ShimadzuLC08	**Project Name:**	Technology Research
**Manual Modified:**	None	**Operator:**	wujialing
**Sample name:**	intermediate Ⅱ(O-WLFX-20210531-2-1)	**Inj. volume:**	10
**Location:**	1:2	**Last Modified By:**	wujialing
**Acq. method:**	Related substance method-S08.amx	**Injection Date:**	2021-05-31 18:33:52+08:00
**Acq.Method Version:**	2021-0518-0105-13016	**Modified Date:**	2021-06-01 08:12:12+08:00
**Pro.Method:**	*Related Substance.pmx	**Printied Date:**	2021-06-01 08:15:29+08:00
**Pro.Method Version:**	2021-0601-0011-32057	**Result Set Version:**	2021-0601-0012-31286

Work station:: Agilent OpenLAB CDS Software Version:2.3(Build 2.3.0.468)

Result Path: /Desvenlafaxine Succinate/Technology Research/Results/Related Substance/Intermediate Ⅱ/20210531-intermediate Ⅱ purity detection (synthesis process optimization).rslt

Data file: intermediate Ⅱ(O-WLFX-20210531-2-1).dx

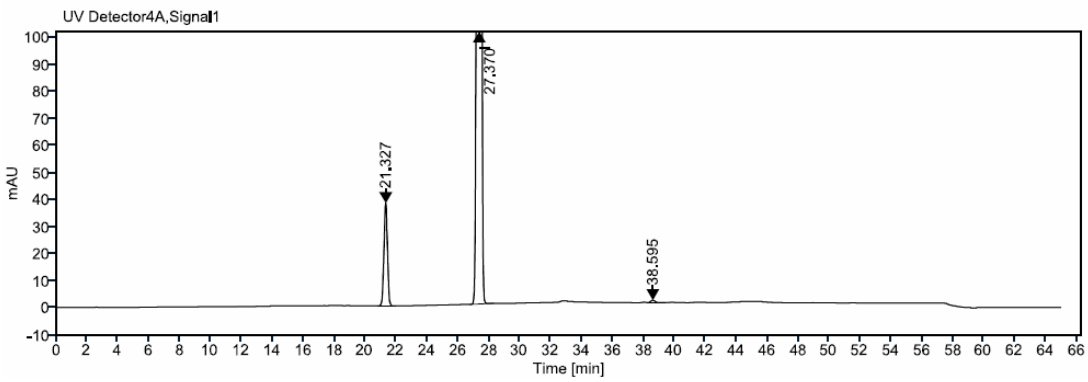

Signal: UV Detector4A,Signal1 Wavelength Ch1 225 nm

Peak Number	RT [min]	Area	Area%	Height	Theoretical Plates	Tail	Resolution
1	21.327	595.36	3.40	37.96	41496	0.99	
2	27.370	16899.30	96.51	1238.89	90708	0.95	15.45
3	38.595	16.10	0.09	0.90	107161	1.06	26.88
	Sum	17510.76	100.00	1277.75			

SI-41. HPLC spectrum of II-5

琥珀酸去甲文拉法辛分析报告

**Instrument:**	ShimadzuLC08	**Project Name:**	Technology Research
**Manual Modified:**	None	**Operator:**	wujialing
**Sample name:**	intermediate Ⅱ(O-WLFX-20210531-2-2)	**Inj. volume:**	10
**Location:**	1:3	**Last Modified By:**	wujialing
**Acq. method:**	Related substance method-S08.amx	**Injection Date:**	2021-05-31 19:39:26+08:00
**Acq.Method Version:**	2021-0518-0105-13016	**Modified Date:**	2021-06-01 08:12:12+08:00
**Pro.Method:**	*Related Substance.pmx	**Printied Date:**	2021-06-01 08:16:21+08:00
**Pro.Method Version:**	2021-0601-0011-32057	**Result Set Version:**	2021-0601-0012-31286

Work station:: Agilent OpenLAB CDS Software Version:2.3(Build 2.3.0.468)

Result Path: /Desvenlafaxine Succinate/Technology Research/Results/Related Substance/Intermediate Ⅱ/20210531-intermediate Ⅱ purity detection (synthesis process optimization).rslt

Data file: intermediate Ⅱ(O-WLFX-20210531-2-2).dx

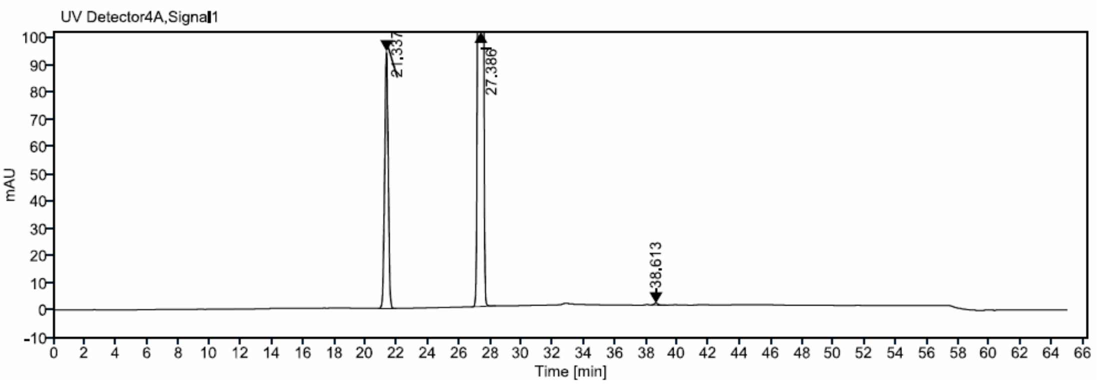

Signal: UV Detector4A,Signal1 Wavelength Ch1 225 nm

Peak Number	RT [min]	Area	Area%	Height	Theoretical Plates	Tail	Resolution
1	21.337	1483.01	6.60	94.32	41332	0.99	
2	27.386	20967.86	93.35	1527.88	89837	0.94	15.40
3	38.613	11.51	0.05	0.63	103122	1.09	26.53
	Sum	22462.38	100.00	1622.83			

SI-42. HPLC spectrum of II-6

琥珀酸去甲文拉法辛分析报告

**Instrument:**	ShimadzuLC08	**Project Name:**	Technology Research
**Manual Modified:**	None	**Operator:**	wujialing
**Sample name:**	intermediate Ⅱ(O-WLFX-20210531-2-3)	**Inj. volume:**	10
**Location:**	1:4	**Last Modified By:**	wujialing
**Acq. method:**	Related substance method-S08.amx	**Injection Date:**	2021-05-31 20:45:00+08:00
**Acq.Method Version:**	2021-0518-0105-13016	**Modified Date:**	2021-06-01 08:12:12+08:00
**Pro.Method:**	*Related Substance.pmx	**Printied Date:**	2021-06-01 08:16:36+08:00
**Pro.Method Version:**	2021-0601-0011-32057	**Result Set Version:**	2021-0601-0012-31286

Work station:: Agilent OpenLAB CDS Software Version:2.3(Build 2.3.0.468)

Result Path: /Desvenlafaxine Succinate/Technology Research/Results/Related Substance/Intermediate Ⅱ/20210531-intermediate Ⅱ purity detection (synthesis process optimization).rslt

Data file: intermediate Ⅱ(O-WLFX-20210531-2-3).dx

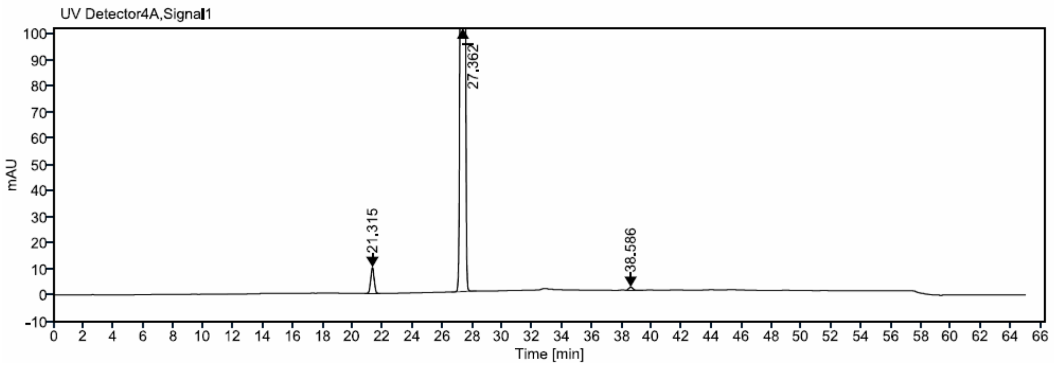

Signal: UV Detector4A,Signal1 Wavelength Ch1 225 nm

Peak Number	RT [min]	Area	Area%	Height	Theoretical Plates	Tail	Resolution
1	21.315	154.95	1.01	9.88	41458	0.99	
2	27.362	15233.16	98.85	1117.72	90788	0.95	15.47
3	38.586	21.92	0.14	1.24	108687	1.05	27.00
	Sum	15410.03	100.00	1128.84			

SI-43. HPLC spectrum of II-7

琥珀酸去甲文拉法辛分析报告

**Instrument:**	ShimadzuLC08	**Project Name:**	Technology Research
**Manual Modified:**	None	**Operator:**	wujialing
**Sample name:**	intermediate Ⅱ(O-WLFX-20210601-2-1)	**Inj. volume:**	10
**Location:**	1:2	**Last Modified By:**	wujialing
**Acq. method:**	Related substance method-S08.amx	**Injection Date:**	2021-06-01 16:44:51+08:00
**Acq.Method Version:**	2021-0518-0105-13016	**Modified Date:**	2021-06-02 08:05:36+08:00
**Pro.Method:**	*Related Substance.pmx	**Printied Date:**	2021-06-02 08:08:00+08:00
**Pro.Method Version:**	2021-0602-0005-12357	**Result Set Version:**	2021-0602-0005-49344

Work station:: Agilent OpenLAB CDS Software Version:2.3(Build 2.3.0.468)

Result Path: /Desvenlafaxine Succinate/Technology Research/Results/Related Substance/Intermediate Ⅱ/20210601-intermediate Ⅱ purity detection (synthesis process optimization).rslt

Data file: intermediate Ⅱ(O-WLFX-20210601-2-1).dx

n

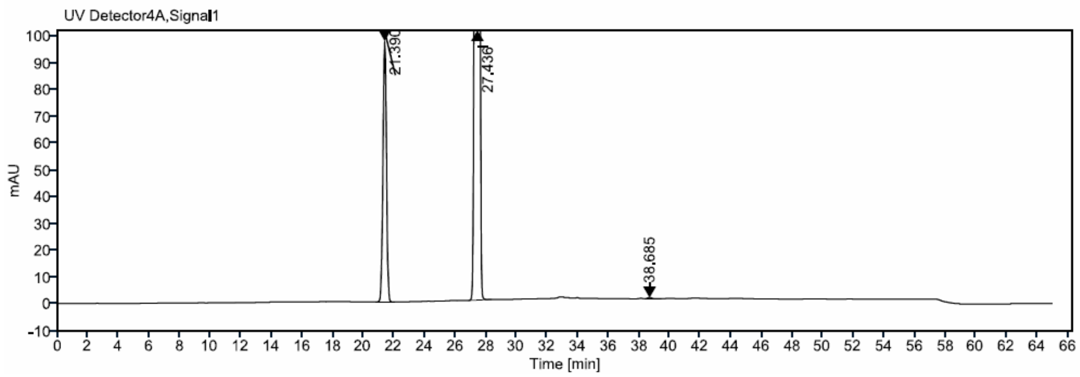

Signal: UV Detector4A,Signal1 Wavelength Ch1 225 nm

Peak Number	RT [min]	Area	Area%	Height	Theoretical Plates	Tail	Resolution
1	21.390	1535.64	6.46	97.33	41266	0.99	
2	27.436	22219.95	93.50	1618.01	90114	0.94	15.37
3	38.685	8.37	0.04	0.44	104132	1.20	26.62
	Sum	23763.95	100.00	1715.78			

SI-44. HPLC spectrum of II-8

琥珀酸去甲文拉法辛分析报告

**Instrument:**	ShimadzuLC08	**Project Name:**	Technology Research
**Manual Modified:**	None	**Operator:**	wujialing
**Sample name:**	intermediate Ⅱ(O-WLFX-20210601-2-3)	**Inj. volume:**	10
**Location:**	1:4	**Last Modified By:**	wujialing
**Acq. method:**	Related substance method-S08.amx	**Injection Date:**	2021-06-01 18:56:00+08:00
**Acq.Method Version:**	2021-0518-0105-13016	**Modified Date:**	2021-06-02 08:05:36+08:00
**Pro.Method:**	*Related Substance.pmx	**Printied Date:**	2021-06-02 08:09:03+08:00
**Pro.Method Version:**	2021-0602-0005-12357	**Result Set Version:**	2021-0602-0005-49344

Work station:: Agilent OpenLAB CDS Software Version:2.3(Build 2.3.0.468)

Result Path: /Desvenlafaxine Succinate/Technology Research/Results/Related Substance/Intermediate Ⅱ/20210601-intermediate Ⅱ purity detection (synthesis process optimization).rslt

Data file: intermediate Ⅱ(O-WLFX-20210601-2-3).dx

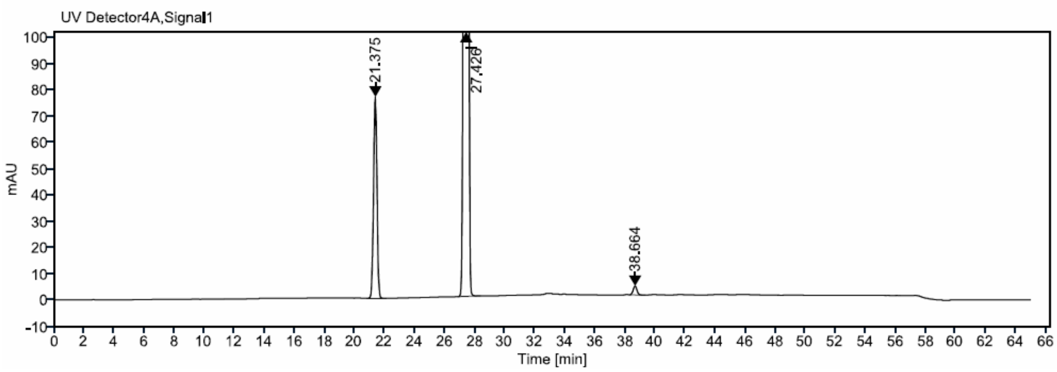

Signal: UV Detector4A,Signal1 Wavelength Ch1 225 nm

Peak Number	RT [min]	Area	Area%	Height	Theoretical Plates	Tail	Resolution
1	21.375	1205.95	5.03	76.60	41390	0.99	
2	27.426	22698.74	94.72	1652.88	90081	0.93	15.40
3	38.664	59.49	0.25	3.40	108455	1.01	26.91
	Sum	23964.18	100.00	1732.89			

SI-45. HPLC spectrum of II-9

琥珀酸去甲文拉法辛分析报告

**Instrument:**	ShimadzuLC08	**Project Name:**	Technology Research
**Manual Modified:**	None	**Operator:**	wujialing
**Sample name:**	intermediate Ⅱ(O-WLFX-20210601-2-3)	**Inj. volume:**	10
**Location:**	1:4	**Last Modified By:**	wujialing
**Acq. method:**	Related substance method-S08.amx	**Injection Date:**	2021-06-01 18:56:00+08:00
**Acq.Method Version:**	2021-0518-0105-13016	**Modified Date:**	2021-06-02 08:05:36+08:00
**Pro.Method:**	*Related Substance.pmx	**Printied Date:**	2021-06-02 08:09:03+08:00
**Pro.Method Version:**	2021-0602-0005-12357	**Result Set Version:**	2021-0602-0005-49344

Work station:: Agilent OpenLAB CDS Software Version:2.3(Build 2.3.0.468)

Result Path: /Desvenlafaxine Succinate/Technology Research/Results/Related Substance/Intermediate Ⅱ/20210601-intermediate Ⅱ purity detection (synthesis process optimization).rslt

Data file: intermediate Ⅱ(O-WLFX-20210601-2-3).dx

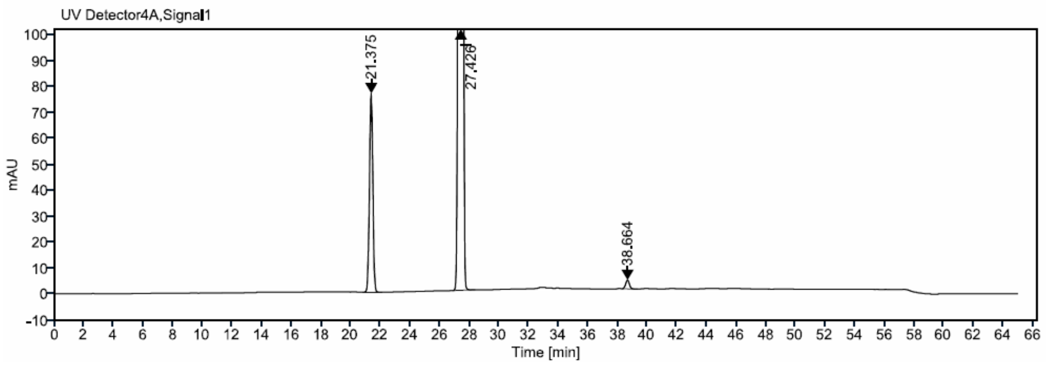

Signal: UV Detector4A,Signal1 Wavelength Ch1 225 nm

Peak Number	RT [min]	Area	Area%	Height	Theoretical Plates	Tail	Resolution
1	21.375	1205.95	5.03	76.60	41390	0.99	
2	27.426	22698.74	94.72	1652.88	90081	0.93	15.40
3	38.664	59.49	0.25	3.40	108455	1.01	26.91
	Sum	23964.18	100.00	1732.89			

SI-46. HPLC spectrum of II-10

琥珀酸去甲文拉法辛分析报告

**Instrument:**	ShimadzuLC08	**Project Name:**	Technology Research
**Manual Modified:**	None	**Operator:**	wujialing
**Sample name:**	intermediate Ⅱ(O-WLFX-20210602-2-1)	**Inj. volume:**	10
**Location:**	1:2	**Last Modified By:**	wujialing
**Acq. method:**	Related substance method-S08.amx	**Injection Date:**	2021-06-02 12:04:27+08:00
**Acq.Method Version:**	2021-0518-0105-13016	**Modified Date:**	2021-06-02 15:24:02+08:00
**Pro.Method:**	*Related Substance.pmx	**Printied Date:**	2021-06-02 15:26:18+08:00
**Pro.Method Version:**	2021-0602-0722-42318	**Result Set Version:**	2021-0602-0725-07520

Work station:: Agilent OpenLAB CDS Software Version:2.3(Build 2.3.0.468)

Result Path: /Desvenlafaxine Succinate/Technology Research/Results/Related Substance/Intermediate Ⅱ/20210602-intermediate Ⅱ purity detection (synthesis process optimization).rslt

Data file: intermediate Ⅱ(O-WLFX-20210602-2-1).dx

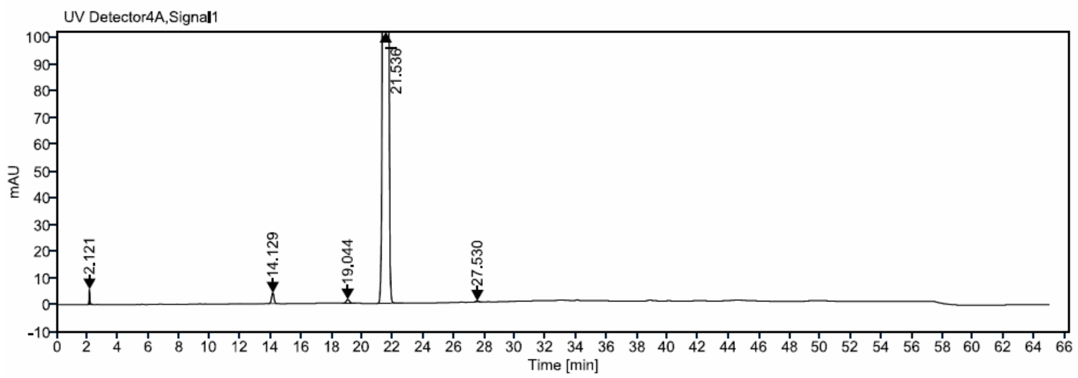

Signal: UV Detector4A,Signal1 Wavelength Ch1 225 nm

Peak Number	RT [min]	Area	Area%	Height	Theoretical Plates	Tail	Resolution
1	2.121	21.35	0.14	5.89	8472	1.44	
2	14.129	43.89	0.28	4.05	37987	0.98	62.84
3	19.044	20.54	0.13	1.44	41983	1.09	14.85
4	21.536	15474.35	99.41	977.16	41542	0.96	6.27
5	27.530	6.09	0.04	0.45	93162	1.21	15.30
	Sum	15566.23	100.00	989.01			

SI-47. HPLC spectrum of II-11

琥珀酸去甲文拉法辛分析报告

**Instrument:**	ShimadzuLC08	**Project Name:**	Technology Research
**Manual Modified:**	None	**Operator:**	wujialing
**Sample name:**	intermediate Ⅱ(O-WLFX-20210602-2-2)	**Inj. volume:**	10
**Location:**	1:3	**Last Modified By:**	wujialing
**Acq. method:**	Related substance method-S08.amx	**Injection Date:**	2021-06-02 13:10:00+08:00
**Acq.Method Version:**	2021-0518-0105-13016	**Modified Date:**	2021-06-02 15:24:02+08:00
**Pro.Method:**	*Related Substance.pmx	**Printied Date:**	2021-06-02 15:27:00+08:00
**Pro.Method Version:**	2021-0602-0722-42318	**Result Set Version:**	2021-0602-0725-07520

Work station:: Agilent OpenLAB CDS Software Version:2.3(Build 2.3.0.468)

Result Path: /Desvenlafaxine Succinate/Technology Research/Results/Related Substance/Intermediate Ⅱ/20210602-intermediate Ⅱ purity detection (synthesis process optimization).rslt

Data file: intermediate Ⅱ(O-WLFX-20210602-2-2).dx

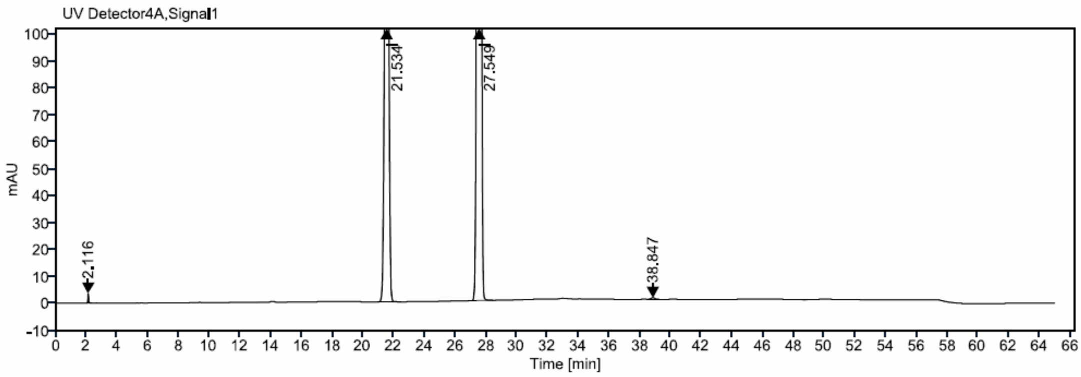

Signal: UV Detector4A,Signal1 Wavelength Ch1 225 nm

Peak Number	RT [min]	Area	Area%	Height	Theoretical Plates	Tail	Resolution
1	2.116	13.45	0.08	3.73	8846	1.52	
2	21.534	4721.38	26.94	301.96	42601	0.98	76.54
3	27.549	12779.57	72.92	954.95	95472	0.96	15.54
4	38.847	12.09	0.07	0.67	105722	1.07	27.08
	Sum	17526.50	100.00	1261.31			

SI-48. HPLC spectrum of II-12

琥珀酸去甲文拉法辛分析报告

**Instrument:**	ShimadzuLC08	**Project Name:**	Technology Research
**Manual Modified:**	None	**Operator:**	wujialing
**Sample name:**	intermediate Ⅱ(O-WLFX-20210602-2-3)	**Inj. volume:**	10
**Location:**	1:4	**Last Modified By:**	wujialing
**Acq. method:**	Related substance method-S08.amx	**Injection Date:**	2021-06-02 14:15:35+08:00
**Acq.Method Version:**	2021-0518-0105-13016	**Modified Date:**	2021-06-02 15:24:02+08:00
**Pro.Method:**	*Related Substance.pmx	**Printied Date:**	2021-06-02 15:27:17+08:00
**Pro.Method Version:**	2021-0602-0722-42318	**Result Set Version:**	2021-0602-0725-07520

Work station:: Agilent OpenLAB CDS Software Version:2.3(Build 2.3.0.468)

Result Path: /Desvenlafaxine Succinate/Technology Research/Results/Related Substance/Intermediate Ⅱ/20210602-intermediate Ⅱ purity detection (synthesis process optimization).rslt

Data file: intermediate Ⅱ(O-WLFX-20210602-2-3).dx

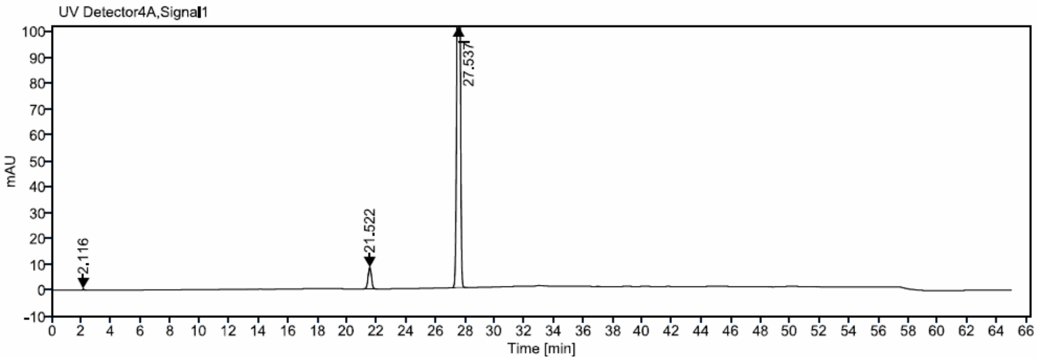

Signal: UV Detector4A,Signal1 Wavelength Ch1 225 nm

Peak Number	RT [min]	Area	Area%	Height	Theoretical Plates	Tail	Resolution
1	2.116	2.97	0.10	0.77	8916	1.76	
2	21.522	130.89	4.25	8.42	42922	0.99	76.82
3	27.537	2948.26	95.66	221.60	96500	0.97	15.62
	Sum	3082.12	100.00	230.79			

SI-49. HPLC spectrum of II-13

琥珀酸去甲文拉法辛分析报告

**Instrument:**	ShimadzuLC08	**Project Name:**	Technology Research
**Manual Modified:**	None	**Operator:**	wujialing
**Sample name:**	intermediate Ⅱ(O-WLFX-20210603-2-1)	**Inj. volume:**	10
**Location:**	1:2	**Last Modified By:**	wujialing
**Acq. method:**	Related substance method-S08.amx	**Injection Date:**	2021-06-03 15:55:33+08:00
**Acq.Method Version:**	2021-0518-0105-13016	**Modified Date:**	2021-06-04 08:10:56+08:00
**Pro.Method:**	*Related Substance.pmx	**Printied Date:**	2021-06-04 08:13:19+08:00
**Pro.Method Version:**	2021-0604-0010-14558	**Result Set Version:**	2021-0604-0011-28616

Work station:: Agilent OpenLAB CDS Software Version:2.3(Build 2.3.0.468)

Result Path: /Desvenlafaxine Succinate/Technology Research/Results/Related Substance/Intermediate Ⅱ/20210603-intermediate Ⅱ purity detection (synthesis process optimization).rslt

Data file: intermediate Ⅱ(O-WLFX-20210603-2-1).dx

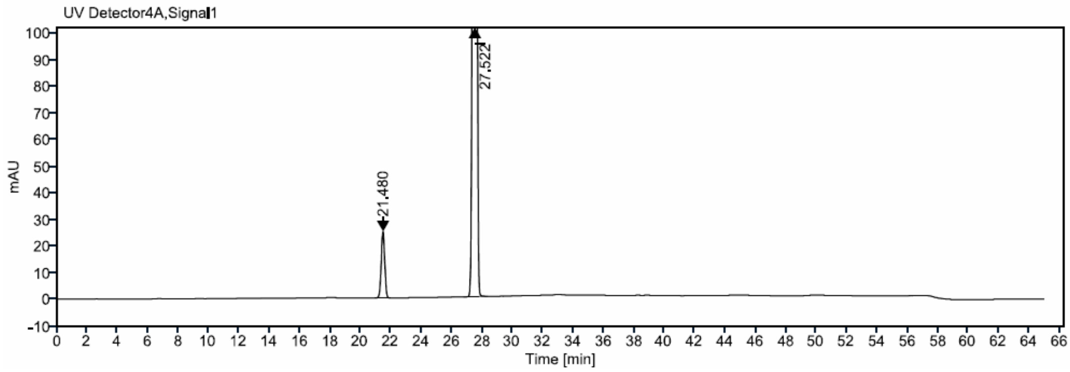

Signal: UV Detector4A,Signal1 Wavelength Ch1 225 nm

Peak Number	RT [min]	Area	Area%	Height	Theoretical Plates	Tail	Resolution
1	21.480	395.93	3.03	24.98	41224	0.99	
2	27.522	12671.11	96.97	936.09	93101	0.96	15.41
	Sum	13067.04	100.00	961.07			

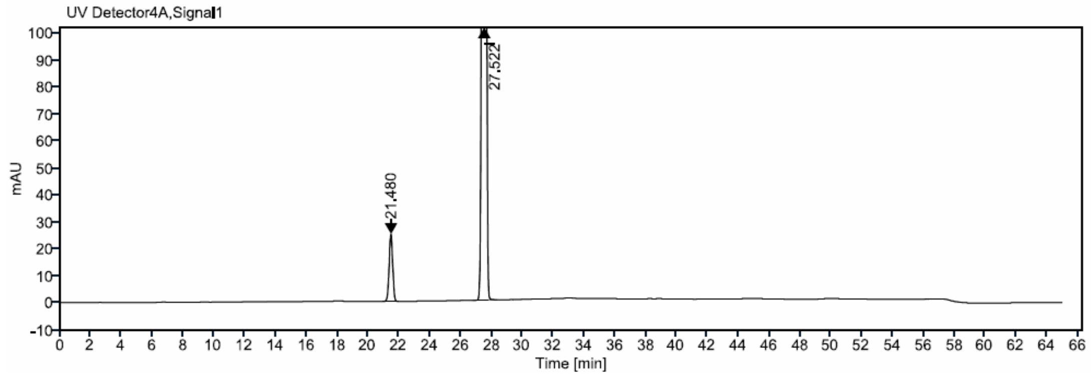

Signal: UV Detector4A,Signal1 Wavelength Ch1 225 nm

Peak Number	RT [min]	Area	Area%	Height	Theoretical Plates	Tail	Resolution
1	21.480	395.93	3.03	24.98	41224	0.99	
2	27.522	12671.11	96.97	936.09	93101	0.96	15.41
	Sum	13067.04	100.00	961.07			

SI-50. HPLC spectrum of II-14

琥珀酸去甲文拉法辛分析报告

**Instrument:**	ShimadzuLC08	**Project Name:**	Technology Research
**Manual Modified:**	None	**Operator:**	wujialing
**Sample name:**	intermediate Ⅱ(O-WLFX-20210603-2-2)	**Inj. volume:**	10
**Location:**	1:3	**Last Modified By:**	wujialing
**Acq. method:**	Related substance method-S08.amx	**Injection Date:**	2021-06-03 17:01:07+08:00
**Acq.Method Version:**	2021-0518-0105-13016	**Modified Date:**	2021-06-04 08:10:56+08:00
**Pro.Method:**	*Related Substance.pmx	**Printied Date:**	2021-06-04 08:14:02+08:00
**Pro.Method Version:**	2021-0604-0010-14558	**Result Set Version:**	2021-0604-0011-28616

Work station:: Agilent OpenLAB CDS Software Version:2.3(Build 2.3.0.468)

Result Path: /Desvenlafaxine Succinate/Technology Research/Results/Related Substance/Intermediate Ⅱ/20210603-intermediate Ⅱ purity detection (synthesis process optimization).rslt

Data file: intermediate Ⅱ(O-WLFX-20210603-2-2).dx

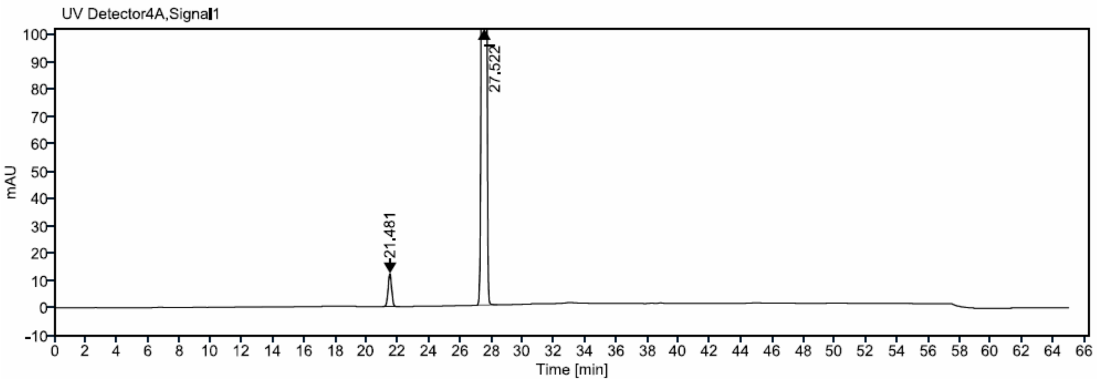

Signal: UV Detector4A,Signal1 Wavelength Ch1 225 nm

Peak Number	RT [min]	Area	Area%	Height	Theoretical Plates	Tail	Resolution
1	21.481	191.70	1.31	12.10	41235	0.99	
2	27.522	14493.00	98.69	1069.49	92892	0.95	15.40
	Sum	14684.70	100.00	1081.58			

SI-51. HPLC spectrum of II-15

琥珀酸去甲文拉法辛分析报告

**Instrument:**	ShimadzuLC08	**Project Name:**	Technology Research
**Manual Modified:**	None	**Operator:**	wujialing
**Sample name:**	intermediate Ⅱ(O-WLFX-20210603-2-3)	**Inj. volume:**	10
**Location:**	1:4	**Last Modified By:**	wujialing
**Acq. method:**	Related substance method-S08.amx	**Injection Date:**	2021-06-03 18:06:41+08:00
**Acq.Method Version:**	2021-0518-0105-13016	**Modified Date:**	2021-06-04 08:10:56+08:00
**Pro.Method:**	*Related Substance.pmx	**Printied Date:**	2021-06-04 08:14:22+08:00
**Pro.Method Version:**	2021-0604-0010-14558	**Result Set Version:**	2021-0604-0011-28616

Work station:: Agilent OpenLAB CDS Software Version:2.3(Build 2.3.0.468)

Result Path: /Desvenlafaxine Succinate/Technology Research/Results/Related Substance/Intermediate Ⅱ/20210603-intermediate Ⅱ purity detection (synthesis process optimization).rslt

Data file: intermediate Ⅱ(O-WLFX-20210603-2-3).dx

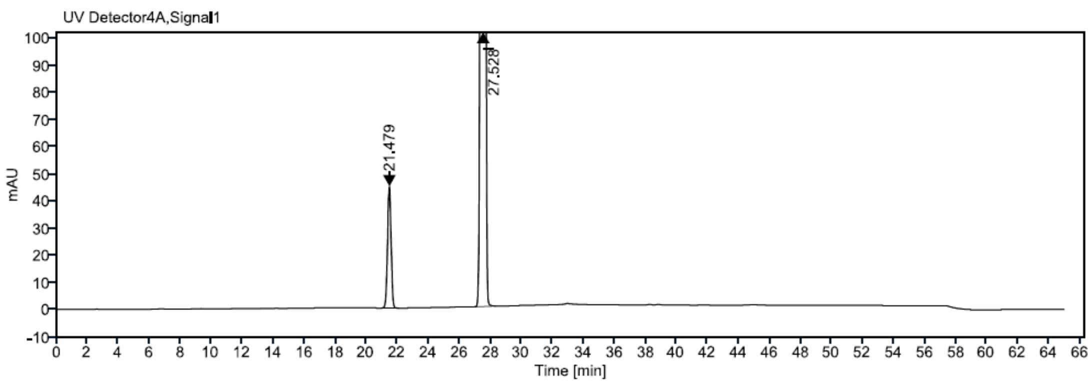

Signal: UV Detector4A,Signal1 Wavelength Ch1 225 nm

Peak Number	RT [min]	Area	Area%	Height	Theoretical Plates	Tail	Resolution
1	21.479	712.46	3.03	44.93	41188	0.99	
2	27.528	22775.99	96.97	1662.71	91190	0.94	15.36
	Sum	23488.45	100.00	1707.64			

SI-52. HPLC spectrum of II-16

琥珀酸去甲文拉法辛分析报告

**Instrument:**	ShimadzuLC08	**Project Name:**	Technology Research
**Manual Modified:**	None	**Operator:**	wujialing
**Sample name:**	intermediate Ⅱ(O-WLFX-20210604-2-1)	**Inj. volume:**	10
**Location:**	1:2	**Last Modified By:**	wujialing
**Acq. method:**	Related substance method-S08.amx	**Injection Date:**	2021-06-04 17:02:47+08:00
**Acq.Method Version:**	2021-0518-0105-13016	**Modified Date:**	2021-06-07 08:30:36+08:00
**Pro.Method:**	*Related Substance.pmx	**Printied Date:**	2021-06-07 08:33:38+08:00
**Pro.Method Version:**	2021-0607-0029-29054	**Result Set Version:**	2021-0607-0030-57168

Work station:: Agilent OpenLAB CDS Software Version:2.3(Build 2.3.0.468)

Result Path: /Desvenlafaxine Succinate/Technology Research/Results/Related Substance/Intermediate Ⅱ/20210604-intermediate Ⅱ purity detection (synthesis process optimization).rslt

Data file: intermediate Ⅱ(O-WLFX-20210604-2-1).dx

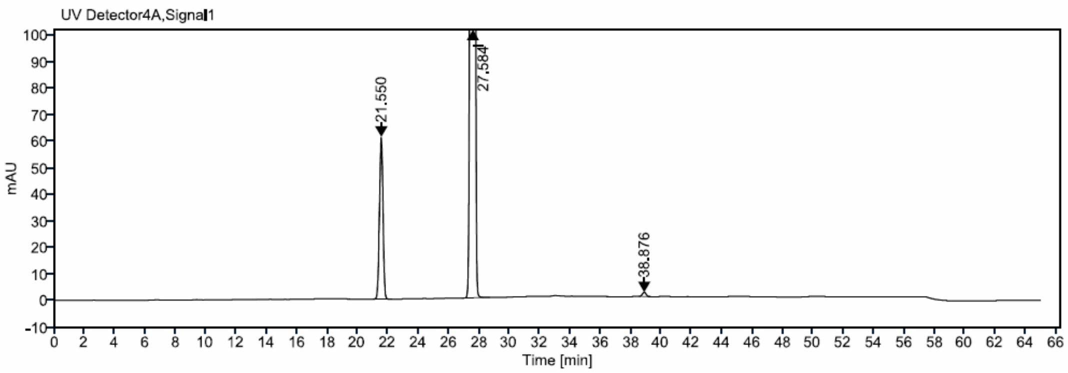

Signal: UV Detector4A,Signal1 Wavelength Ch1 225 nm

Peak Number	RT [min]	Area	Area%	Height	Theoretical Plates	Tail	Resolution
1	21.550	974.33	4.36	61.14	41069	0.99	
2	27.584	21343.59	95.51	1564.66	92269	0.94	15.30
3	38.876	28.77	0.13	1.64	107640	1.01	26.98
	Sum	22346.69	100.00	1627.44			

SI-53. HPLC spectrum of II-17

琥珀酸去甲文拉法辛分析报告

**Instrument:**	ShimadzuLC08	**Project Name:**	Technology Research
**Manual Modified:**	None	**Operator:**	wujialing
**Sample name:**	intermediate Ⅱ(O-WLFX-20210604-2-2)	**Inj. volume:**	10
**Location:**	1:3	**Last Modified By:**	wujialing
**Acq. method:**	Related substance method-S08.amx	**Injection Date:**	2021-06-04 18:08:21+08:00
**Acq.Method Version:**	2021-0518-0105-13016	**Modified Date:**	2021-06-07 08:30:36+08:00
**Pro.Method:**	*Related Substance.pmx	**Printied Date:**	2021-06-07 08:34:30+08:00
**Pro.Method Version:**	2021-0607-0029-29054	**Result Set Version:**	2021-0607-0030-57168

Work station:: Agilent OpenLAB CDS Software Version:2.3(Build 2.3.0.468)

Result Path: /Desvenlafaxine Succinate/Technology Research/Results/Related Substance/Intermediate Ⅱ/20210604-intermediate Ⅱ purity detection (synthesis process optimization).rslt

Data file: intermediate Ⅱ(O-WLFX-20210604-2-2).dx

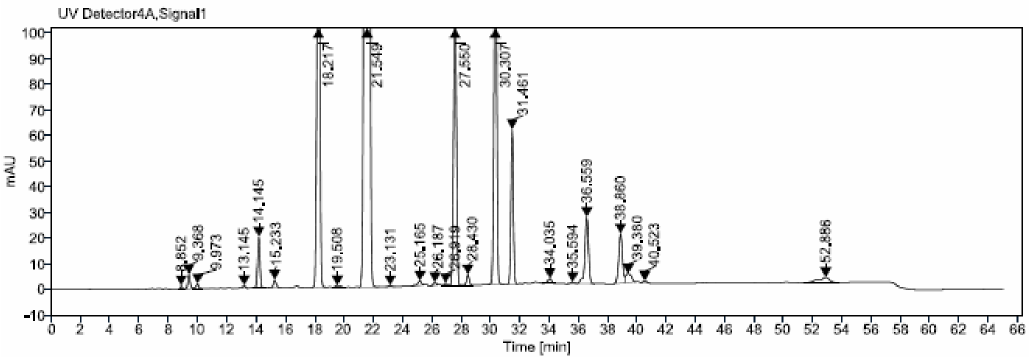

Signal: UV Detector4A,Signal1 Wavelength Ch1 225 nm

Peak Number	RT [min]	Area	Area%	Height	Theoretical Plates	Tail	Resolution
1	8.852	9.20	0.03	1.08	24068	1.06	
2	9.368	54.87	0.19	5.38	19793	1.18	2.09
3	9.973	20.47	0.07	1.74	17185	0.80	2.12
4	13.145	8.84	0.03	0.88	37656	0.86	11.03
5	14.145	222.68	0.79	19.82	35888	0.97	3.51
6	15.233	41.18	0.15	2.95	28098	1.18	3.29
7	18.217	3120.15	11.06	245.87	46266	0.99	8.50
8	19.508	13.33	0.05	0.58	18399	1.39	2.83
9	21.549	19167.12	67.92	1193.30	40438	0.95	4.06
10	23.131	7.35	0.03	0.45	43024	1.16	3.62

琥珀酸去甲文拉法辛分析报告

Peak Number	RT [min]	Area	Area%	Height	Theoretical Plates	Tail	Resolution
11	25.165	31.74	0.11	1.72	52913	0.86	4.61
12	26.187	13.53	0.05	1.02	85357	1.07	2.57
13	26.919	9.58	0.03	0.79	104464	1.01	2.12
14	27.550	1610.46	5.71	120.06	94844	0.98	1.83
15	28.430	53.55	0.19	4.11	107256	1.03	2.50
16	30.307	2081.00	7.37	170.19	138347	0.99	5.58
17	31.461	721.98	2.56	60.44	155756	0.99	3.58
18	34.035	34.61	0.12	1.92	79943	0.80	6.43
19	35.594	8.92	0.03	0.53	104548	0.96	3.38
20	36.559	435.81	1.54	26.04	122339	0.78	2.25
21	38.860	350.17	1.24	19.30	104718	1.02	5.12
22	39.380	91.79	0.33	3.40	33841	1.61	0.78
23	40.523	13.55	0.05	0.83	132322	0.92	1.76
24	52.886	96.65	0.34	1.98	47988	0.75	17.52
	Sum	28218.51	100.00	1884.39			

SI-54. HPLC spectrum of II-18

琥珀酸去甲文拉法辛分析报告

**Instrument:**	ShimadzuLC08	**Project Name:**	Technology Research
**Manual Modified:**	None	**Operator:**	wujialing
**Sample name:**	intermediate Ⅱ(O-WLFX-20210604-2-3)	**Inj. volume:**	10
**Location:**	1:4	**Last Modified By:**	wujialing
**Acq. method:**	Related substance method-S08.amx	**Injection Date:**	2021-06-04 19:13:55+08:00
**Acq.Method Version:**	2021-0518-0105-13016	**Modified Date:**	2021-06-07 08:30:36+08:00
**Pro.Method:**	*Related Substance.pmx	**Printied Date:**	2021-06-07 08:35:08+08:00
**Pro.Method Version:**	2021-0607-0029-29054	**Result Set Version:**	2021-0607-0030-57168

Work station:: Agilent OpenLAB CDS Software Version:2.3(Build 2.3.0.468)

Result Path: /Desvenlafaxine Succinate/Technology Research/Results/Related Substance/Intermediate Ⅱ/20210604-intermediate Ⅱ purity detection (synthesis process optimization).rslt

Data file: intermediate Ⅱ(O-WLFX-20210604-2-3).dx

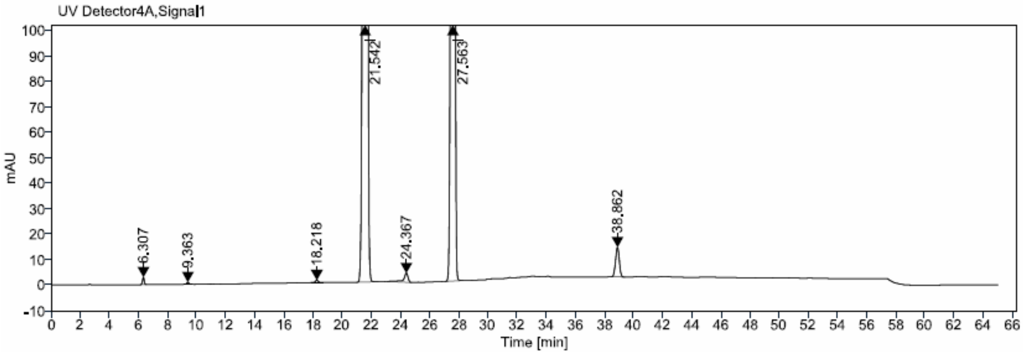

Signal: UV Detector4A,Signal1 Wavelength Ch1 225 nm

Peak Number	RT [min]	Area	Area%	Height	Theoretical Plates	Tail	Resolution
1	6.307	24.23	0.09	2.90	12703	0.99	
2	9.363	7.78	0.03	0.84	22458	0.99	12.90
3	18.218	11.77	0.04	0.92	46051	1.03	30.04
4	21.542	13589.33	51.28	850.12	40784	0.96	8.67
5	24.367	80.39	0.30	3.63	47949	0.59	6.48
6	27.563	12576.68	47.46	931.88	93913	0.96	7.94
7	38.862	207.86	0.78	11.67	106234	0.98	27.01
	Sum	26498.04	100.00	1801.95			

SI-55. HPLC spectrum of II-19

琥珀酸去甲文拉法辛分析报告

**Instrument:**	ShimadzuLC08	**Project Name:**	Technology Research
**Manual Modified:**	None	**Operator:**	wujialing
**Sample name:**	intermediate Ⅱ(O-WLFX-20210608-2-1)	**Inj. volume:**	10
**Location:**	1:2	**Last Modified By:**	wujialing
**Acq. method:**	Related substance method-S08.amx	**Injection Date:**	2021-06-08 18:05:21+08:00
**Acq.Method Version:**	2021-0518-0105-13016	**Modified Date:**	2021-06-09 08:06:40+08:00
**Pro.Method:**	*Related Substance.pmx	**Printied Date:**	2021-06-09 08:08:40+08:00
**Pro.Method Version:**	2021-0609-0006-24517	**Result Set Version:**	2021-0609-0006-53137

Work station:: Agilent OpenLAB CDS Software Version:2.3(Build 2.3.0.468)

Result Path: /Desvenlafaxine Succinate/Technology Research/Results/Related Substance/Intermediate Ⅱ/20210608-intermediate Ⅱ purity detection (synthesis process optimization).rslt

Data file: intermediate Ⅱ(O-WLFX-20210608-2-1).dx

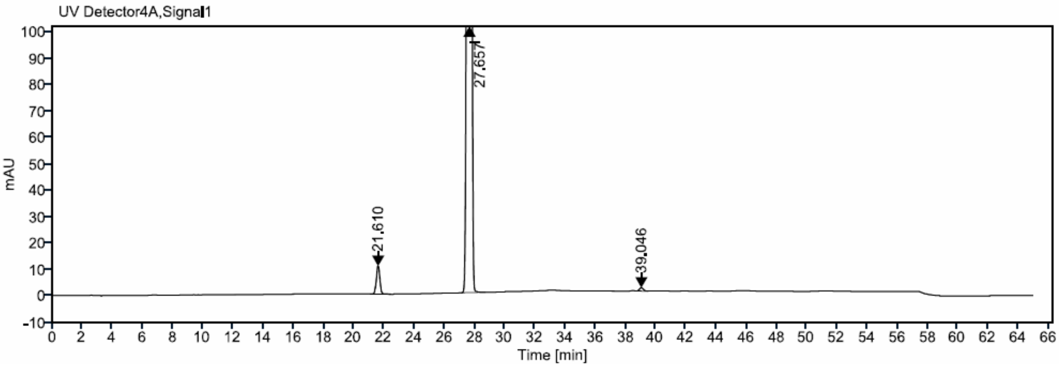

Signal: UV Detector4A,Signal1 Wavelength Ch1 225 nm

Peak Number	RT [min]	Area	Area%	Height	Theoretical Plates	Tail	Resolution
1	21.610	169.87	0.77	10.62	40948	0.99	
2	27.657	21927.73	99.13	1606.53	92610	0.94	15.29
3	39.046	22.07	0.10	1.26	108994	1.01	27.23
	Sum	22119.68	100.00	1618.41			

SI-56. HPLC spectrum of II-20

琥珀酸去甲文拉法辛分析报告

**Instrument:**	ShimadzuLC08	**Project Name:**	Technology Research
**Manual Modified:**	None	**Operator:**	wujialing
**Sample name:**	intermediate Ⅱ(O-WLFX-20210608-2-2)	**Inj. volume:**	10
**Location:**	1:3	**Last Modified By:**	wujialing
**Acq. method:**	Related substance method-S08.amx	**Injection Date:**	2021-06-08 19:10:55+08:00
**Acq.Method Version:**	2021-0518-0105-13016	**Modified Date:**	2021-06-09 08:06:40+08:00
**Pro.Method:**	*Related Substance.pmx	**Printied Date:**	2021-06-09 08:09:25+08:00
**Pro.Method Version:**	2021-0609-0006-24517	**Result Set Version:**	2021-0609-0006-53137
**Work station::**	Agilent OpenLAB CDS Software Version:2.3(Build 2.3.0.468)		
**Result Path:**	/Desvenlafaxine Succinate/Technology Research/Results/Related Substance/Intermediate Ⅱ/20210608-intermediate Ⅱ purity detection (synthesis process optimization).rslt		
**Data file:**	intermediate Ⅱ(O-WLFX-20210608-2-2).dx		

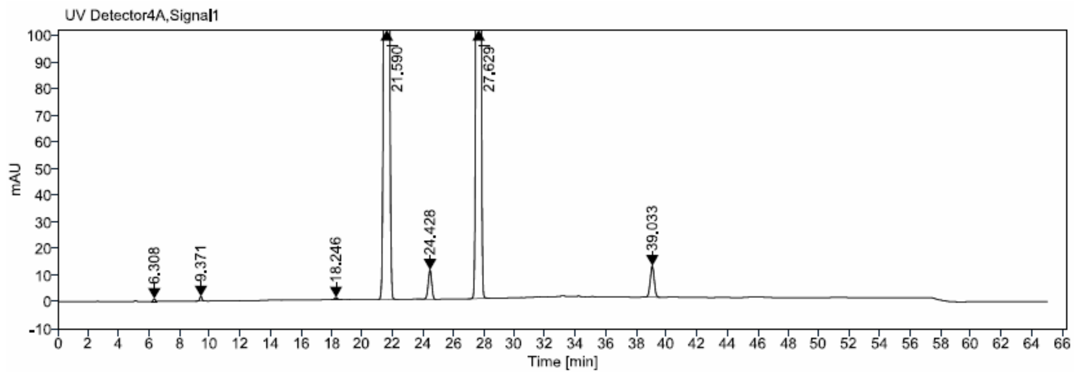

Signal: UV Detector4A,Signal1 Wavelength Ch1 225 nm

Peak Number	RT [min]	Area	Area%	Height	Theoretical Plates	Tail	Resolution
1	6.308	8.15	0.03	0.99	13019	1.01	
2	9.371	16.87	0.06	1.79	22597	0.99	13.02
3	18.246	10.99	0.04	0.86	45763	1.00	30.06
4	21.590	14014.53	49.51	871.46	40464	0.96	8.68
5	24.428	177.24	0.63	11.11	54407	0.95	6.69
6	27.629	13868.93	48.99	1024.63	93787	0.96	8.21
7	39.033	211.58	0.75	11.76	104775	0.99	27.05
	Sum	28308.28	100.00	1922.59			

SI-57. HPLC spectrum of II-21

琥珀酸去甲文拉法辛分析报告

**Instrument:**	ShimadzuLC08	**Project Name:**	Technology Research
**Manual Modified:**	None	**Operator:**	wujialing
**Sample name:**	intermediate Ⅱ(O-WLFX-20210608-2-3)	**Inj. volume:**	10
**Location:**	1:4	**Last Modified By:**	wujialing
**Acq. method:**	Related substance method-S08.amx	**Injection Date:**	2021-06-08 20:16:28+08:00
**Acq.Method Version:**	2021-0518-0105-13016	**Modified Date:**	2021-06-09 08:06:40+08:00
**Pro.Method:**	*Related Substance.pmx	**Printied Date:**	2021-06-09 08:09:45+08:00
**Pro.Method Version:**	2021-0609-0006-24517	**Result Set Version:**	2021-0609-0006-53137

Work station:: Agilent OpenLAB CDS Software Version:2.3(Build 2.3.0.468)

Result Path: /Desvenlafaxine Succinate/Technology Research/Results/Related Substance/Intermediate Ⅱ/20210608-intermediate Ⅱ purity detection (synthesis process optimization).rslt

Data file: intermediate Ⅱ(O-WLFX-20210608-2-3).dx

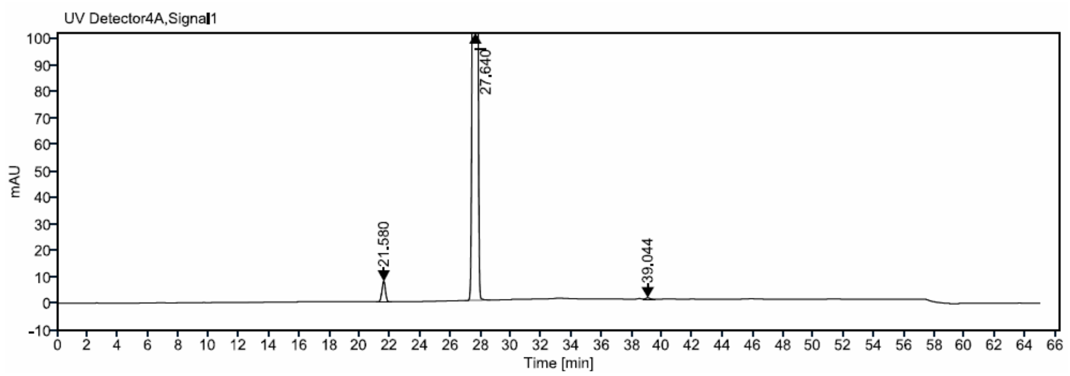

Signal: UV Detector4A,Signal1 Wavelength Ch1 225 nm

Peak Number	RT [min]	Area	Area%	Height	Theoretical Plates	Tail	Resolution
1	21.580	123.40	0.55	7.70	40810	0.99	
2	27.640	22504.61	99.42	1644.32	92055	0.94	15.31
3	39.044	8.54	0.04	0.49	108464	1.04	27.20
	Sum	22636.55	100.00	1652.51			

SI-58. HPLC spectrum of O-desvenlafaxine impurity E

琥珀酸去甲文拉法辛分析报告

**Instrument:**	ShimadzuLC08	**Project Name:**	Technology Research
**Manual Modified:**	None	**Operator:**	wujialing
**Sample name:**	intermediate III (O-WLFX-20210508-3)	**Inj. volume:**	10
**Location:**	1:2	**Last Modified By:**	wujialing
**Acq. method:**	Related substance method-S08.amx	**Injection Date:**	2021-05-10 13:31:39+08:00
**Acq.Method Version:**	2021-0420-0710-54193	**Modified Date:**	2021-05-10 14:52:57+08:00
**Pro.Method:**	*Related Substance.pmx	**Printied Date:**	2021-05-10 14:56:00+08:00
**Pro.Method Version:**	2021-0510-0651-42618	**Result Set Version:**	2021-0510-0653-15289

Work station:: Agilent OpenLAB CDS Software Version:2.3(Build 2.3.0.468)

Result Path: /Desvenlafaxine Succinate/Technology Research/Results/Related Substance/Intermediate 3/20210510-intermediate III purity detection (synthesis process optimization).rslt

Data file: intermediate III (O-WLFX-20210508-3).dx

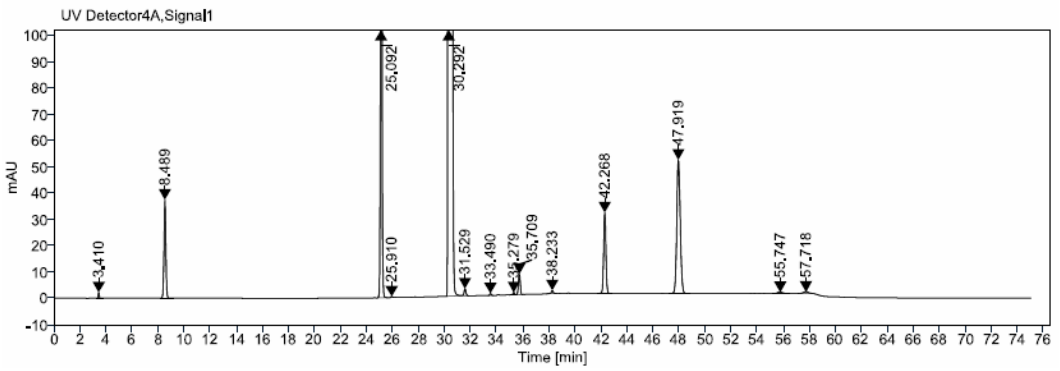

Signal: UV Detector4A,Signal1 Wavelength Ch1 225 nm

Peak Number	RT [min]	Area	Area%	Height	Theoretical Plates	Tail	Resolution
1	3.410	10.97	0.04	2.32	12205	1.15	
2	8.489	376.30	1.47	37.45	16191	1.03	26.02
3	25.092	1626.55	6.37	202.57	220533	1.14	69.10
4	25.910	2.66	0.01	0.38	296117	1.11	4.05
5	30.292	22063.84	86.38	1686.57	102582	2.40	15.41
6	31.529	25.49	0.10	2.66	239589	1.11	3.89
7	33.490	5.59	0.02	0.74	452033	1.11	8.58
8	35.279	7.16	0.03	0.87	407955	1.07	8.51
9	35.709	76.59	0.30	7.82	295229	0.97	1.78
10	38.233	14.24	0.06	1.26	388709	0.94	9.93

琥珀酸去甲文拉法辛分析报告

Peak Number	RT [min]	Area	Area%	Height	Theoretical Plates	Tail	Resolution
11	42.268	369.38	1.45	30.82	285443	1.03	14.37
12	47.919	935.72	3.66	50.88	153136	1.12	14.02
13	55.747	12.97	0.05	0.47	95009	0.98	12.90
14	57.718	15.46	0.06	0.53	88038	1.02	2.63
	Sum	25542.92	100.00	2025.34			

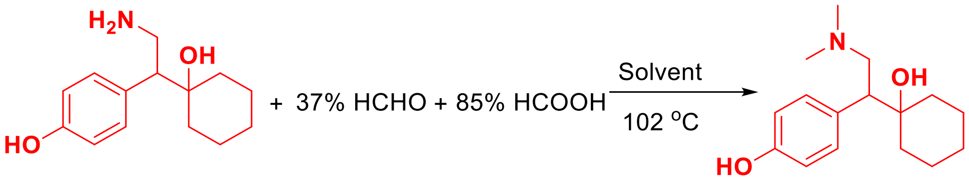

III

ODV

SI-59. HPLC spectrum of ODV-1

琥珀酸去甲文拉法辛分析报告

Instrument: ShimadzuLC08

Project Name: Technology Research

Operator: wujialing

Manual Modified: None

Sample name: Desvenlafaxine (O-WLFX-20210721-P-1-1)

Inj. volume: 10

Location: 1:2

Last Modified By: wujialing

Acq. method: Related substance method-11-S08.amx

Injection Date: 2021-07-21 17:07:22+08:00

Acq.Method Version: 2021-0720-0726-37551

Modified Date: 2021-07-22 08:10:27+08:00

Pro.Method: *Related Substance.pmx

Printied Date: 2021-07-22 08:12:16+08:00

Pro.Method Version: 2021-0722-0007-19625

Result Set Version: 2021-0722-0010-48810

Work station:: Agilent OpenLAB CDS Software Version:2.3(Build 2.3.0.468)

Result Path: /Desvenlafaxine Succinate/Technology Research/Results/Related Substance/Desvenlafaxine Succinate/20210721-Desvenlafaxine purity detection (synthesis process optimization)-1.rslt

Data file: Desvenlafaxine (O-WLFX-20210721-P-1-1).dx

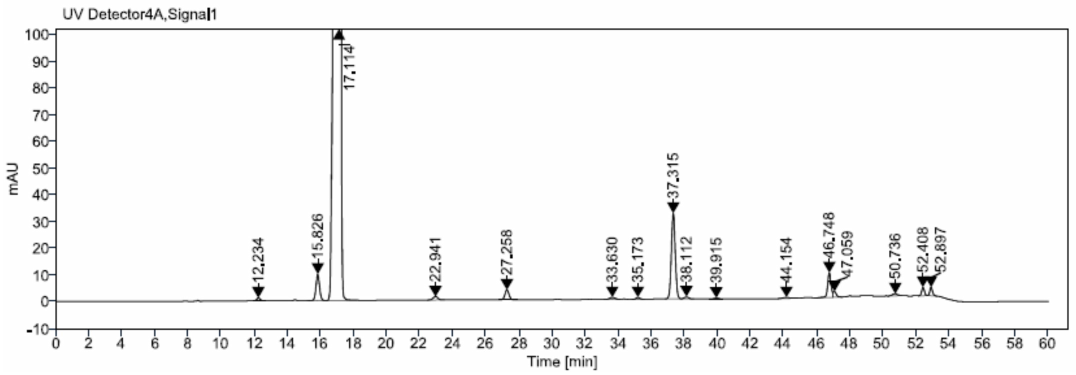

Signal: UV Detector4A,Signal1 Wavelength Ch1 225 nm

Peak Number	RT [min]	Area	Area%	Height	Theoretical Plates	Tail	Resolution
1	12.234	15.31	0.07	1.11	18345	1.15	
2	15.826	148.77	0.70	9.96	25997	1.06	9.52
3	17.114	20107.03	94.87	1111.61	18782	0.68	2.89
4	22.941	27.30	0.13	1.30	27204	1.03	11.04
5	27.258	63.35	0.30	3.74	58332	1.06	8.57
6	33.630	17.55	0.08	0.74	43773	1.17	11.64
7	35.173	10.49	0.05	0.65	106356	1.16	2.87
8	37.315	524.16	2.47	32.02	116462	1.04	4.93
9	38.112	11.97	0.06	0.67	98249	0.93	1.72
10	39.915	5.23	0.02	0.29	103347	1.14	3.67

琥珀酸去甲文拉法辛分析报告

Peak Number	RT [min]	Area	Area%	Height	Theoretical Plates	Tail	Resolution
11	44.154	9.84	0.05	0.36	61049	1.49	7.00
12	46.748	133.77	0.63	9.23	258767	1.00	4.79
13	47.059	27.45	0.13	2.15	94195	3.06	0.63
14	50.736	17.29	0.08	0.82	141488	1.09	6.38
15	52.408	36.13	0.17	3.17	465055	1.13	3.95
16	52.897	37.59	0.18	3.23	463288	1.06	1.58
	Sum	21193.22	100.00	1181.05			

SI-60. HPLC spectrum of ODV-2

琥珀酸去甲文拉法辛分析报告

**Instrument:**	ShimadzuLC08	**Project Name:**	Technology Research
**Manual Modified:**	None	**Operator:**	wujialing
**Sample name:**	Desvenlafaxine (O-WLFX-20210721-P-2-1)	**Inj. volume:**	10
**Location:**	1:3	**Last Modified By:**	wujialing
**Acq. method:**	Related substance method-11-S08.amx	**Injection Date:**	2021-07-21 18:07:58+08:00
**Acq.Method Version:**	2021-0720-0726-37551	**Modified Date:**	2021-07-22 08:10:27+08:00
**Pro.Method:**	*Related Substance.pmx	**Printied Date:**	2021-07-22 08:12:59+08:00
**Pro.Method Version:**	2021-0722-0007-19625	**Result Set Version:**	2021-0722-0010-48810

Work station:: Agilent OpenLAB CDS Software Version:2.3(Build 2.3.0.468)

Result Path: /Desvenlafaxine Succinate/Technology Research/Results/Related Substance/Desvenlafaxine Succinate/20210721-Desvenlafaxine purity detection (synthesis process optimization)-1.rslt

Data file: Desvenlafaxine (O-WLFX-20210721-P-2-1).dx

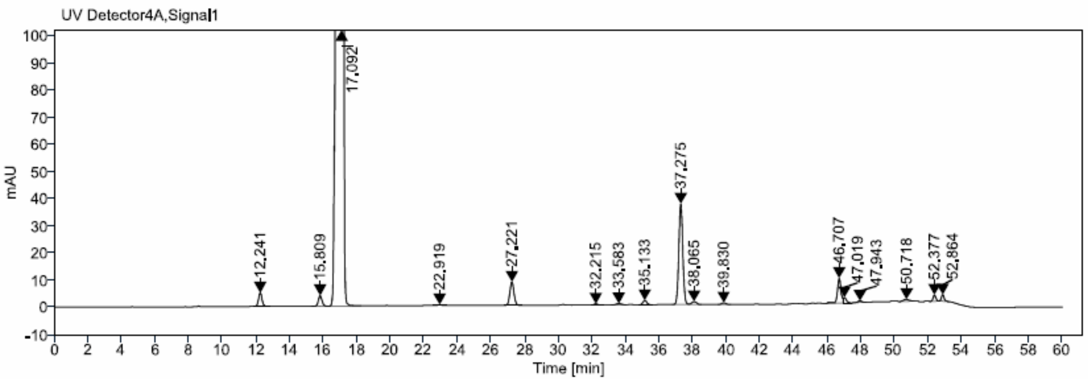

Signal: UV Detector4A,Signal1 Wavelength Ch1 225 nm

Peak Number	RT [min]	Area	Area%	Height	Theoretical Plates	Tail	Resolution
1	12.241	71.83	0.35	5.01	16918	1.10	
2	15.809	58.68	0.28	3.94	26063	1.11	9.29
3	17.092	19529.68	94.17	1090.77	19119	0.69	2.89
4	22.919	4.96	0.02	0.24	28866	1.10	11.27
5	27.221	145.90	0.70	8.63	58349	1.06	8.69
6	32.215	6.83	0.03	0.46	104084	1.05	11.75
7	33.583	11.90	0.06	0.51	44867	1.17	2.64
8	35.133	25.42	0.12	1.56	101778	1.10	2.89
9	37.275	606.84	2.93	37.02	115699	1.04	4.87
10	38.065	18.22	0.09	1.01	98337	0.96	1.71

琥珀酸去甲文拉法辛分析报告

Peak Number	RT [min]	Area	Area%	Height	Theoretical Plates	Tail	Resolution
11	39.830	9.00	0.04	0.56	135802	1.09	3.85
12	46.707	140.08	0.68	9.12	256972	0.97	17.17
13	47.019	25.97	0.13	2.02	91703	2.52	0.63
14	47.943	10.34	0.05	0.60	191893	1.41	1.75
15	50.718	17.08	0.08	0.77	127265	1.27	5.51
16	52.377	27.30	0.13	2.40	463811	1.12	3.79
17	52.864	29.50	0.14	2.50	455572	1.05	1.57
	Sum	20739.54	100.00	1167.12			

SI-61. HPLC spectrum of ODV-3

琥珀酸去甲文拉法辛分析报告

**Instrument:**	ShimadzuLC08	**Project Name:**	Technology Research
**Manual Modified:**	None	**Operator:**	wujialing
**Sample name:**	Desvenlafaxine (O-WLFX-20210721-P-3-1)	**Inj. volume:**	10
**Location:**	1:4	**Last Modified By:**	wujialing
**Acq. method:**	Related substance method-11-S08.amx	**Injection Date:**	2021-07-21 19:08:32+08:00
**Acq.Method Version:**	2021-0720-0726-37551	**Modified Date:**	2021-07-22 08:10:27+08:00
**Pro.Method:**	*Related Substance.pmx	**Printied Date:**	2021-07-22 08:13:23+08:00
**Pro.Method Version:**	2021-0722-0007-19625	**Result Set Version:**	2021-0722-0010-48810

Work station:: Agilent OpenLAB CDS Software Version:2.3(Build 2.3.0.468)

Result Path: /Desvenlafaxine Succinate/Technology Research/Results/Related Substance/Desvenlafaxine Succinate/20210721-Desvenlafaxine purity detection (synthesis process optimization)-1.rslt

Data file: Desvenlafaxine (O-WLFX-20210721-P-3-1).dx

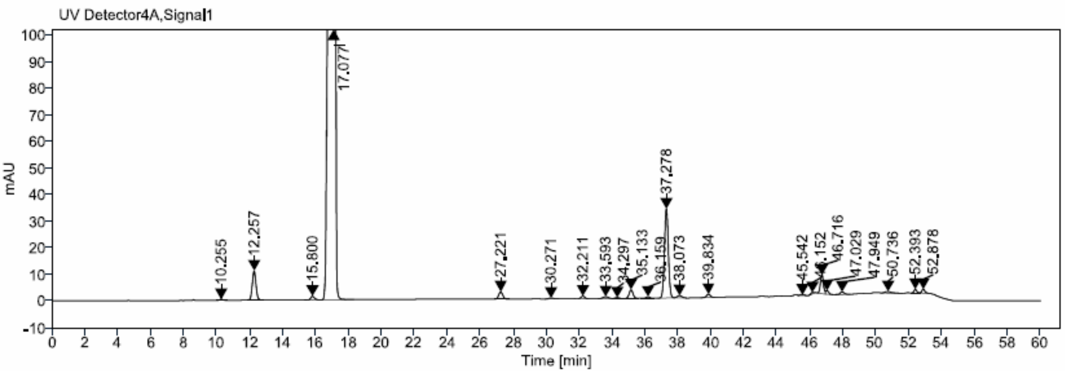

Signal: UV Detector4A,Signal1 Wavelength Ch1 225 nm

Peak Number	RT [min]	Area	Area%	Height	Theoretical Plates	Tail	Resolution
1	10.255	4.87	0.02	0.38	15067	1.14	
2	12.257	168.07	0.85	11.05	14814	1.04	5.43
3	15.800	21.55	0.11	1.39	24735	1.24	8.81
4	17.077	18740.06	94.38	1061.43	19601	0.69	2.87
5	27.221	46.29	0.23	2.75	58585	1.06	21.63
6	30.271	7.17	0.04	0.37	54054	1.29	6.29
7	32.211	13.60	0.07	0.94	107890	0.95	4.25
8	33.593	16.31	0.08	0.71	45528	1.13	2.70
9	34.297	6.26	0.03	0.38	90452	0.96	1.30
10	35.133	53.42	0.27	3.25	102335	1.06	1.87

琥珀酸去甲文拉法辛分析报告

Peak Number	RT [min]	Area	Area%	Height	Theoretical Plates	Tail	Resolution
11	36.159	8.33	0.04	0.54	126633	1.28	2.42
12	37.278	549.03	2.77	33.53	116015	1.04	2.65
13	38.073	15.95	0.08	0.88	98561	0.98	1.72
14	39.834	18.87	0.10	1.18	139630	1.18	3.86
15	45.542	4.00	0.02	0.22	151079	0.92	12.76
16	46.152	7.58	0.04	0.62	317106	1.24	1.53
17	46.716	90.41	0.46	6.96	290086	1.07	1.67
18	47.029	15.54	0.08	1.33	165940	1.51	0.77
19	47.949	16.79	0.08	0.99	202163	1.60	2.07
20	50.736	17.38	0.09	0.66	108070	1.15	5.34
21	52.393	15.68	0.08	1.39	472811	1.11	3.59
22	52.878	17.93	0.09	1.49	447213	1.02	1.56
	Sum	19855.07	100.00	1132.44			

SI-62. HPLC spectrum of ODV-4

琥珀酸去甲文拉法辛分析报告

**Instrument:**	ShimadzuLC08	**Project Name:**	Technology Research
**Manual Modified:**	None	**Operator:**	wujialing
**Sample name:**	Desvenlafaxine (O-WLFX-20210723-P-1-1)	**Inj. volume:**	10
**Location:**	1:5	**Last Modified By:**	wujialing
**Acq. method:**	Related substance method-11-S08.amx	**Injection Date:**	2021-07-23 22:16:51+08:00
**Acq.Method Version:**	2021-0720-0726-37551	**Modified Date:**	2021-07-26 08:41:07+08:00
**Pro.Method:**	*Related Substance.pmx	**Printied Date:**	2021-07-26 08:48:14+08:00
**Pro.Method Version:**	2021-0726-0040-26813	**Result Set Version:**	2021-0726-0041-15761

Work station:: Agilent OpenLAB CDS Software Version:2.3(Build 2.3.0.468)

Result Path: /Desvenlafaxine Succinate/Technology Research/Results/Related Substance/Desvenlafaxine Succinate/20210723-Desvenlafaxine purity detection (synthesis process optimization).rslt

Data file: Desvenlafaxine (O-WLFX-20210723-P-1-1).dx

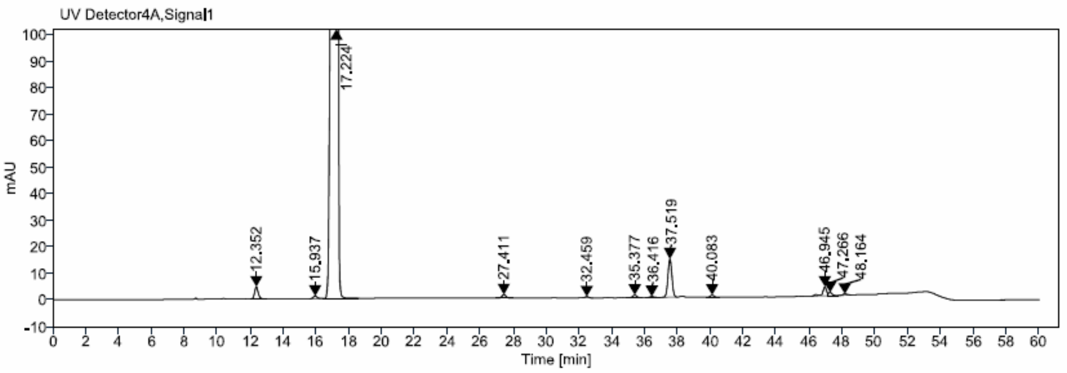

Signal: UV Detector4A,Signal1 Wavelength Ch1 225 nm

Peak Number	RT [min]	Area	Area%	Height	Theoretical Plates	Tail	Resolution
1	12.352	67.17	0.33	4.59	16380	1.14	
2	15.937	14.93	0.07	0.98	25427	1.21	9.12
3	17.224	19669.33	97.64	1092.49	19165	0.69	2.87
4	27.411	23.23	0.12	1.36	58252	1.06	21.41
5	32.459	7.59	0.04	0.52	107263	0.97	11.86
6	35.377	17.12	0.08	0.96	98106	0.93	6.88
7	36.416	6.37	0.03	0.39	120769	1.49	2.38
8	37.519	238.50	1.18	14.38	115152	1.02	2.56
9	40.083	11.69	0.06	0.74	145223	1.07	5.94
10	46.945	61.22	0.30	3.37	247043	0.65	17.19

琥珀酸去甲文拉法辛分析报告

Peak Number	RT [min]	Area	Area%	Height	Theoretical Plates	Tail	Resolution
11	47.266	18.17	0.09	1.27	156218	2.30	0.75
12	48.164	9.26	0.05	0.50	171087	1.44	1.90
	Sum	20144.59	100.00	1121.56			

SI-63. HPLC spectrum of ODV-5

琥珀酸去甲文拉法辛分析报告

**Instrument:**	ShimadzuLC08	**Project Name:**	Technology Research
**Manual Modified:**	None	**Operator:**	wujialing
**Sample name:**	Desvenlafaxine (O-WLFX-20210723-P-2-1)	**Inj. volume:**	10
**Location:**	1:6	**Last Modified By:**	wujialing
**Acq. method:**	Related substance method-11-S08.amx	**Injection Date:**	2021-07-23 23:17:27+08:00
**Acq.Method Version:**	2021-0720-0726-37551	**Modified Date:**	2021-07-26 08:41:07+08:00
**Pro.Method:**	*Related Substance.pmx	**Printied Date:**	2021-07-26 08:48:50+08:00
**Pro.Method Version:**	2021-0726-0040-26813	**Result Set Version:**	2021-0726-0041-15761

Work station:: Agilent OpenLAB CDS Software Version:2.3(Build 2.3.0.468)

Result Path: /Desvenlafaxine Succinate/Technology Research/Results/Related Substance/Desvenlafaxine Succinate/20210723-Desvenlafaxine purity detection (synthesis process optimization).rslt

Data file: Desvenlafaxine (O-WLFX-20210723-P-2-1).dx

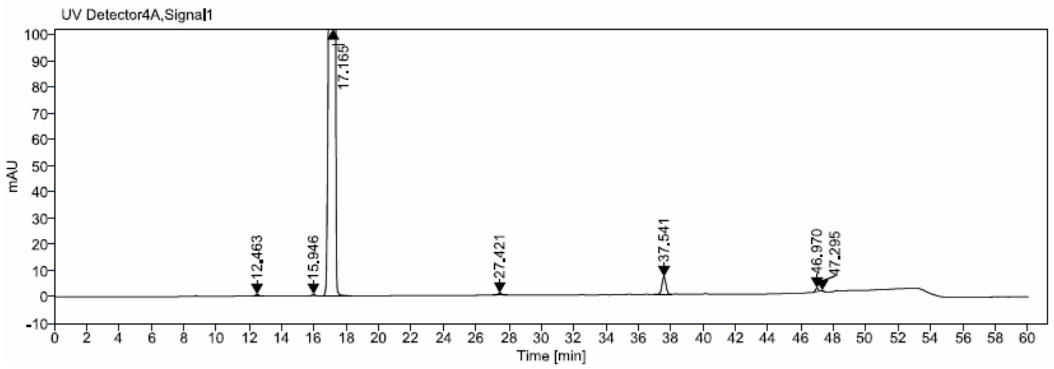

Signal: UV Detector4A,Signal1 Wavelength Ch1 225 nm

Peak Number	RT [min]	Area	Area%	Height	Theoretical Plates	Tail	Resolution
1	12.463	8.88	0.07	0.66	19504	1.09	
2	15.946	6.66	0.05	0.47	28331	1.24	9.47
3	17.165	13035.58	98.77	810.56	24009	0.74	2.96
4	27.421	15.24	0.12	0.90	58692	1.06	22.90
5	37.541	112.20	0.85	6.75	114794	1.02	22.59
6	46.970	15.77	0.12	1.37	353133	1.16	24.83
7	47.295	3.75	0.03	0.38	498007	0.99	1.11
	Sum	13198.07	100.00	821.08			

SI-64. HPLC spectrum of ODV-6

琥珀酸去甲文拉法辛分析报告

**Instrument:**	ShimadzuLC08	**Project Name:**	Technology Research
**Manual Modified:**	None	**Operator:**	wujialing
**Sample name:**	Desvenlafaxine (O-WLFX-20210723-P-3-1)	**Inj. volume:**	10
**Location:**	1:7	**Last Modified By:**	wujialing
**Acq. method:**	Related substance method-11-S08.amx	**Injection Date:**	2021-07-24 00:18:03+08:00
**Acq.Method Version:**	2021-0720-0726-37551	**Modified Date:**	2021-07-26 08:41:07+08:00
**Pro.Method:**	*Related Substance.pmx	**Printied Date:**	2021-07-26 08:49:11+08:00
**Pro.Method Version:**	2021-0726-0040-26813	**Result Set Version:**	2021-0726-0041-15761

Work station:: Agilent OpenLAB CDS Software Version:2.3(Build 2.3.0.468)

Result Path: /Desvenlafaxine Succinate/Technology Research/Results/Related Substance/Desvenlafaxine Succinate/20210723-Desvenlafaxine purity detection (synthesis process optimization).rslt

Data file: Desvenlafaxine (O-WLFX-20210723-P-3-1).dx

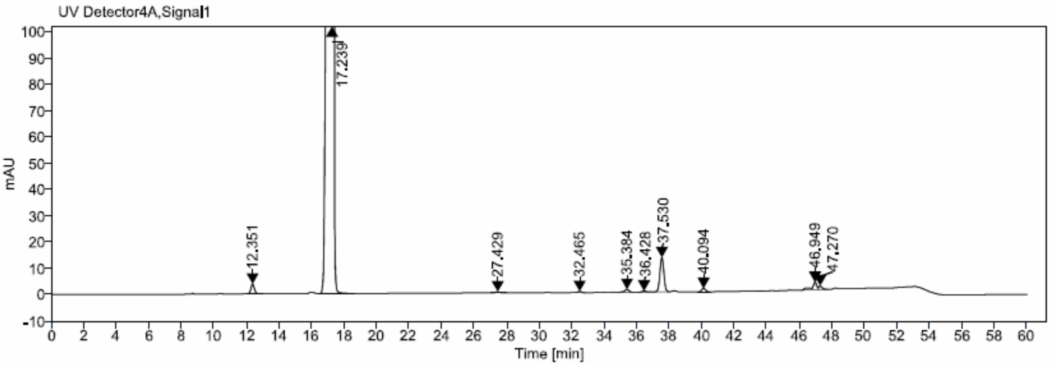

Signal: UV Detector4A,Signal1 Wavelength Ch1 225 nm

Peak Number	RT [min]	Area	Area%	Height	Theoretical Plates	Tail	Resolution
1	12.351	55.04	0.26	3.80	16824	1.13	
2	17.239	20666.17	98.00	1132.23	18717	0.68	11.04
3	27.429	8.55	0.04	0.50	59109	1.10	21.34
4	32.465	6.78	0.03	0.47	109567	0.97	11.94
5	35.384	22.15	0.11	1.12	89019	0.84	6.74
6	36.428	9.48	0.04	0.46	93647	1.62	2.20
7	37.530	217.57	1.03	13.08	114836	1.00	2.40
8	40.094	20.31	0.10	1.28	142841	1.09	5.91
9	46.949	60.11	0.29	2.90	238285	0.64	16.95
10	47.270	21.64	0.10	1.41	158320	2.26	0.75
	Sum	21087.80	100.00	1157.25			

SI-65. HPLC spectrum of ODV-7

琥珀酸去甲文拉法辛分析报告

**Instrument:**	ShimadzuLC08	**Project Name:**	Technology Research
**Manual Modified:**	None	**Operator:**	wujialing
**Sample name:**	Desvenlafaxine (O-WLFX-20210727-P-1-1)	**Inj. volume:**	10
**Location:**	1:5	**Last Modified By:**	wujialing
**Acq. method:**	Related substance method-11-S08.amx	**Injection Date:**	2021-07-27 17:33:01+08:00
**Acq.Method Version:**	2021-0720-0726-37551	**Modified Date:**	2021-07-28 07:57:56+08:00
**Pro.Method:**	*Related Substance.pmx	**Printied Date:**	2021-07-28 08:23:41+08:00
**Pro.Method Version:**	2021-0727-2356-31828	**Result Set Version:**	2021-0728-0000-20376

Work station:: Agilent OpenLAB CDS Software Version:2.3(Build 2.3.0.468)

Result Path: /Desvenlafaxine Succinate/Technology Research/Results/Related Substance/Desvenlafaxine Succinate/20210727-Desvenlafaxine purity detection (synthesis process optimization).rslt

Data file: Desvenlafaxine (O-WLFX-20210727-P-1-1).dx

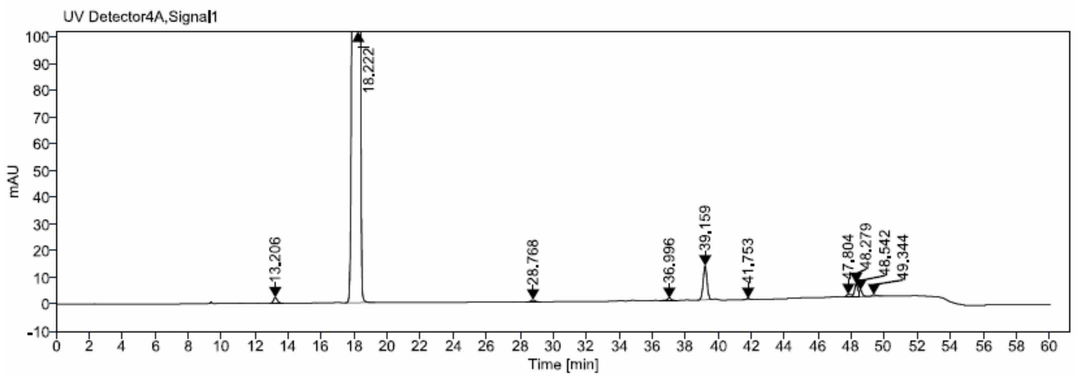

Signal: UV Detector4A,Signal1 Wavelength Ch1 225 nm

Peak Number	RT [min]	Area	Area%	Height	Theoretical Plates	Tail	Resolution
1	13.206	33.30	0.16	2.07	14854	1.16	
2	18.222	20934.29	98.13	1152.88	20951	0.69	10.71
3	28.768	12.27	0.06	0.72	63063	1.10	21.93
4	36.996	15.56	0.07	0.84	99564	0.90	17.75
5	39.159	210.84	0.99	12.74	124675	1.04	4.74
6	41.753	6.52	0.03	0.41	153964	1.27	5.97
7	47.804	17.89	0.08	0.87	54912	1.21	9.75
8	48.279	62.64	0.29	4.86	296918	0.89	0.81
9	48.542	29.65	0.14	2.35	157381	2.07	0.62
10	49.344	11.29	0.05	0.62	187049	1.85	1.70
	Sum	21334.27	100.00	1178.34			

SI-66. HPLC spectrum of ODV-8

琥珀酸去甲文拉法辛分析报告

**Instrument:**	ShimadzuLC08	**Project Name:**	Technology Research
**Manual Modified:**	None	**Operator:**	wujialing
**Sample name:**	Desvenlafaxine (O-WLFX-20210727-P-2-1)	**Inj. volume:**	10
**Location:**	1:6	**Last Modified By:**	wujialing
**Acq. method:**	Related substance method-11-S08.amx	**Injection Date:**	2021-07-27 18:33:37+08:00
**Acq.Method Version:**	2021-0720-0726-37551	**Modified Date:**	2021-07-28 07:57:56+08:00
**Pro.Method:**	*Related Substance.pmx	**Printed Date:**	2021-07-28 08:24:32+08:00
**Pro.Method Version:**	2021-0727-2356-31828	**Result Set Version:**	2021-0728-0000-20376

Work station:: Agilent OpenLAB CDS Software Version:2.3(Build 2.3.0.468)

Result Path: /Desvenlafaxine Succinate/Technology Research/Results/Related Substance/Desvenlafaxine Succinate/20210727-Desvenlafaxine purity detection (synthesis process optimization).rslt

Data file: Desvenlafaxine (O-WLFX-20210727-P-2-1).dx

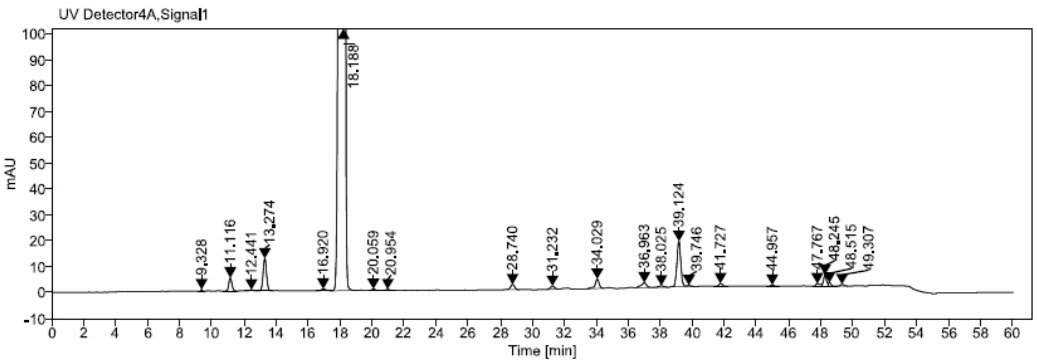

Signal: UV Detector4A,Signal1 Wavelength Ch1 225 nm

Peak Number	RT [min]	Area	Area%	Height	Theoretical Plates	Tail	Resolution
1	9.328	3.75	0.02	0.70	74624	1.00	
2	11.116	70.26	0.39	5.15	15286	1.12	7.21
3	12.441	7.55	0.04	0.48	18926	1.57	3.67
4	13.274	183.09	1.01	12.46	18236	1.03	2.20
5	16.920	12.91	0.07	0.69	19265	1.41	8.28
6	18.188	17298.26	95.05	1008.83	23562	0.71	2.64
7	20.059	8.70	0.05	0.58	40440	1.07	4.28
8	20.954	8.46	0.05	0.46	31962	0.91	2.06
9	28.740	33.33	0.18	2.01	65750	0.99	16.98
10	31.232	22.53	0.12	1.28	68046	1.17	5.37

琥珀酸去甲文拉法辛分析报告

Peak Number	RT [min]	Area	Area%	Height	Theoretical Plates	Tail	Resolution
11	34.029	64.58	0.35	3.65	106304	0.79	6.24
12	36.963	42.69	0.23	1.91	79972	0.86	6.24
13	38.025	10.61	0.06	0.61	127708	1.14	2.24
14	39.124	294.10	1.62	17.53	121468	1.02	2.51
15	39.746	6.55	0.04	0.41	131133	1.00	1.40
16	41.727	18.54	0.10	1.11	140318	1.06	4.48
17	44.957	8.94	0.05	0.54	164940	1.23	7.27
18	47.767	20.17	0.11	1.04	105036	1.23	5.44
19	48.245	55.98	0.31	4.41	326510	0.95	1.03
20	48.515	18.13	0.10	1.42	134999	2.32	0.62
21	49.307	10.58	0.06	0.71	261933	1.44	1.73
	Sum	18199.72	100.00	1065.99			

SI-67. HPLC spectrum of ODV-9

琥珀酸去甲文拉法辛分析报告

**Instrument:**	ShimadzuLC08	**Project Name:**	Technology Research
**Manual Modified:**	None	**Operator:**	wujialing
**Sample name:**	Desvenlafaxine (O-WLFX-20210727-P-3-1)	**Inj. volume:**	10
**Location:**	1:7	**Last Modified By:**	wujialing
**Acq. method:**	Related substance method-11-S08.amx	**Injection Date:**	2021-07-27 19:34:11+08:00
**Acq.Method Version:**	2021-0720-0726-37551	**Modified Date:**	2021-07-28 07:57:56+08:00
**Pro.Method:**	*Related Substance.pmx	**Printied Date:**	2021-07-28 08:25:03+08:00
**Pro.Method Version:**	2021-0727-2356-31828	**Result Set Version:**	2021-0728-0000-20376

Work station:: Agilent OpenLAB CDS Software Version:2.3(Build 2.3.0.468)

Result Path: /Desvenlafaxine Succinate/Technology Research/Results/Related Substance/Desvenlafaxine Succinate/20210727-Desvenlafaxine purity detection (synthesis process optimization).rslt

Data file: Desvenlafaxine (O-WLFX-20210727-P-3-1).dx

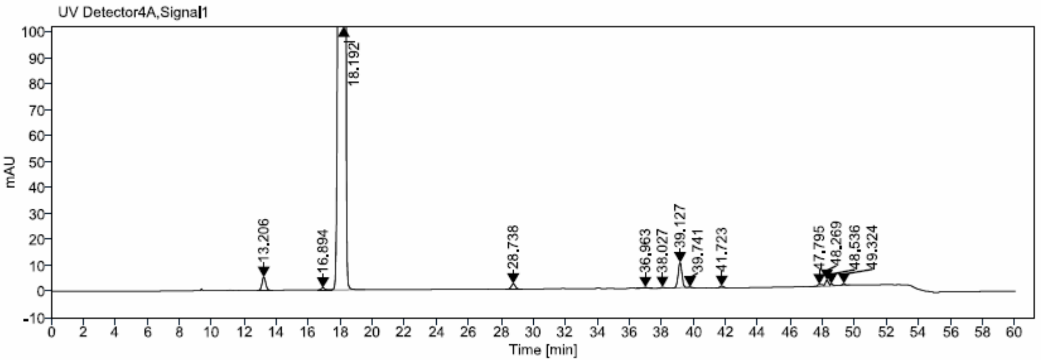

Signal: UV Detector4A,Signal1 Wavelength Ch1 225 nm

Peak Number	RT [min]	Area	Area%	Height	Theoretical Plates	Tail	Resolution
1	13.206	79.81	0.39	5.05	15590	1.10	
2	16.894	13.07	0.06	0.83	26756	1.30	8.82
3	18.192	20148.98	98.11	1126.04	21516	0.70	2.85
4	28.738	34.86	0.17	2.03	62697	1.06	22.08
5	36.963	6.44	0.03	0.34	97402	0.95	17.63
6	38.027	5.77	0.03	0.36	132922	1.22	2.39
7	39.127	162.78	0.79	9.72	122037	1.01	2.54
8	39.741	4.82	0.02	0.29	127008	0.99	1.37
9	41.723	11.11	0.05	0.70	155560	1.10	4.56
10	47.795	16.70	0.08	0.77	72223	1.25	10.70

琥珀酸去甲文拉法辛分析报告

Peak Number	RT [min]	Area	Area%	Height	Theoretical Plates	Tail	Resolution
11	48.269	30.77	0.15	2.41	311317	0.97	0.90
12	48.536	11.58	0.06	0.91	151536	2.88	0.64
13	49.324	11.04	0.05	0.75	264680	1.49	1.79
	Sum	20537.73	100.00	1150.20			

SI-68. HPLC spectrum of ODV-10

琥珀酸去甲文拉法辛分析报告

**Instrument:**	ShimadzuLC08	**Project Name:**	Technology Research
**Manual Modified:**	None	**Operator:**	wujialing
**Sample name:**	Desvenlafaxine (O-WLFX-20210729-P-1-1)	**Inj. volume:**	10
**Location:**	1:5	**Last Modified By:**	wujialing
**Acq. method:**	Related substance method-11-S08.amx	**Injection Date:**	2021-07-29 18:31:23+08:00
**Acq.Method Version:**	2021-0720-0726-37551	**Modified Date:**	2021-07-30 08:14:48+08:00
**Pro.Method:**	*Related Substance.pmx	**Printied Date:**	2021-07-30 08:20:02+08:00
**Pro.Method Version:**	2021-0730-0010-14803	**Result Set Version:**	2021-0730-0015-31270

Work station:: Agilent OpenLAB CDS Software Version:2.3(Build 2.3.0.468)

Result Path: /Desvenlafaxine Succinate/Technology Research/Results/Related Substance/Desvenlafaxine Succinate/20210729-Desvenlafaxine purity detection (synthesis process optimization).rslt

Data file: Desvenlafaxine (O-WLFX-20210729-P-1-1).dx

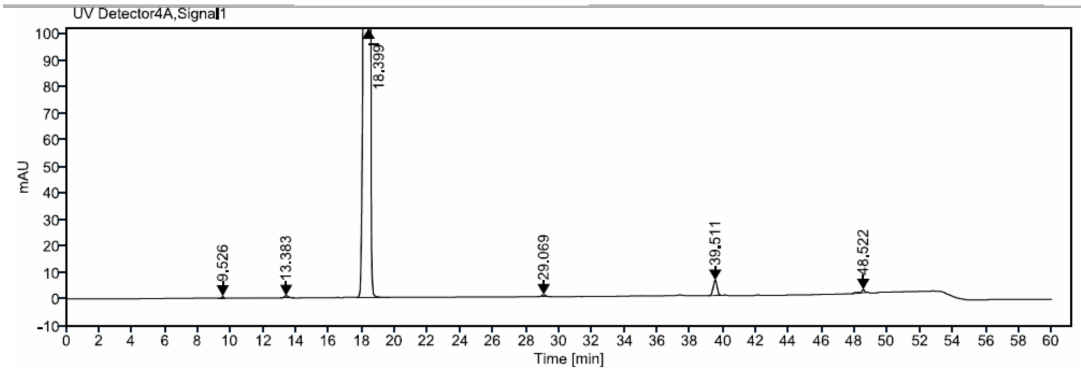

Signal: UV Detector4A,Signal1 Wavelength Ch1 225 nm

Peak Number	RT [min]	Area	Area%	Height	Theoretical Plates	Tail	Resolution
1	9.526	4.05	0.03	0.74	77725	0.70	
2	13.383	13.75	0.09	0.86	15524	1.26	13.62
3	18.399	15992.45	99.13	930.62	24164	0.73	11.11
4	29.069	12.18	0.08	0.71	63989	1.09	22.87
5	39.511	93.46	0.58	5.74	130493	1.04	23.28
6	48.522	16.32	0.10	1.17	502221	1.17	25.33
	Sum	16132.21	100.00	939.83			

SI-69. HPLC spectrum of ODV-11

琥珀酸去甲文拉法辛分析报告

Instrument: ShimadzuLC08

Project Name: Technology Research

Manual Modified: None

Operator: wujialing

Sample name: Desvenlafaxine (O-WLFX-20210729-P-2-1)

Inj. volume: 10

Location: 1:6

Last Modified By: wujialing

Acq. method: Related substance method-11-S08.amx

Injection Date: 2021-07-29 19:31:57+08:00

Acq.Method Version: 2021-0720-0726-37551

Modified Date: 2021-07-30 08:14:48+08:00

Pro.Method: *Related Substance.pmx

Printied Date: 2021-07-30 08:20:43+08:00

Pro.Method Version: 2021-0730-0010-14803

Result Set Version: 2021-0730-0015-31270

Work station:: Agilent OpenLAB CDS Software Version:2.3(Build 2.3.0.468)

Result Path: /Desvenlafaxine Succinate/Technology Research/Results/Related Substance/Desvenlafaxine Succinate/20210729-Desvenlafaxine purity detection (synthesis process optimization).rslt

Data file: Desvenlafaxine (O-WLFX-20210729-P-2-1).dx

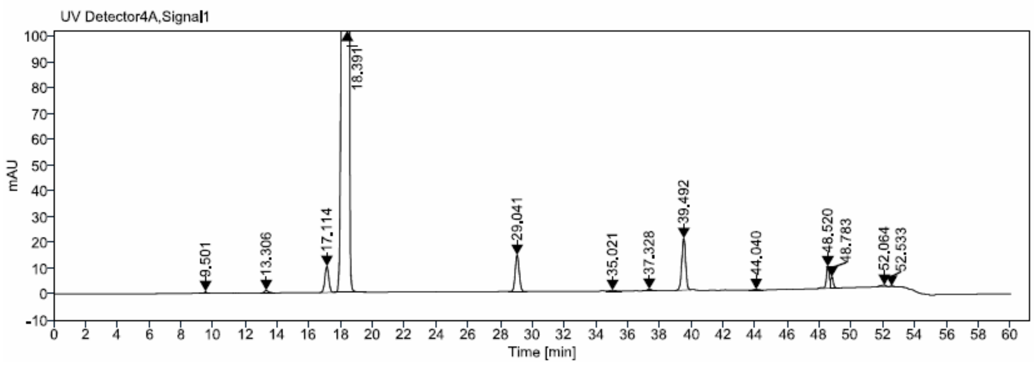

Signal: UV Detector4A,Signal1 Wavelength Ch1 225 nm

Peak Number	RT [min]	Area	Area%	Height	Theoretical Plates	Tail	Resolution
1	9.501	4.09	0.02	0.74	78282	0.99	
2	13.306	15.72	0.08	0.98	16223	1.29	13.74
3	17.114	170.93	0.85	10.16	23171	1.01	8.78
4	18.391	19198.91	95.12	1049.66	21291	0.70	2.68
5	29.041	245.22	1.21	14.21	63686	1.06	22.09
6	35.021	6.68	0.03	0.28	47396	1.09	10.84
7	37.328	11.44	0.06	0.68	111813	1.08	4.23
8	39.492	336.59	1.67	20.14	127209	0.99	4.86
9	44.040	5.83	0.03	0.27	100155	1.19	9.10
10	48.520	111.11	0.55	8.44	316085	0.89	9.94

琥珀酸去甲文拉法辛分析报告

Peak Number	RT [min]	Area	Area%	Height	Theoretical Plates	Tail	Resolution
11	48.783	52.61	0.26	4.45	208939	1.62	0.68
12	52.064	16.88	0.08	0.71	109502	0.83	6.21
13	52.533	7.00	0.03	0.36	356888	1.11	0.95
	Sum	20183.00	100.00	1111.11			

SI-70. HPLC spectrum of ODV-12

琥珀酸去甲文拉法辛分析报告

**Instrument:**	ShimadzuLC08	**Project Name:**	Technology Research
**Manual Modified:**	None	**Operator:**	wujialing
**Sample name:**	Desvenlafaxine (O-WLFX-20210731-P-3-1)	**Inj. volume:**	10
**Location:**	1:3	**Last Modified By:**	wujialing
**Acq. method:**	Related substance method-11-S08.amx	**Injection Date:**	2021-08-03 16:59:40+08:00
**Acq.Method Version:**	2021-0720-0726-37551	**Modified Date:**	2021-08-04 08:22:22+08:00
**Pro.Method:**	*Related Substance.pmx	**Printied Date:**	2021-08-04 08:25:04+08:00
**Pro.Method Version:**	2021-0804-0021-55126	**Result Set Version:**	2021-0804-0022-32034

Work station:: Agilent OpenLAB CDS Software Version:2.3(Build 2.3.0.468)

Result Path: /Desvenlafaxine Succinate/Technology Research/Results/Related Substance/Desvenlafaxine Succinate/20210803-Desvenlafaxine purity detection (synthesis process optimization).rslt

Data file: Desvenlafaxine (O-WLFX-20210731-P-3-1).dx

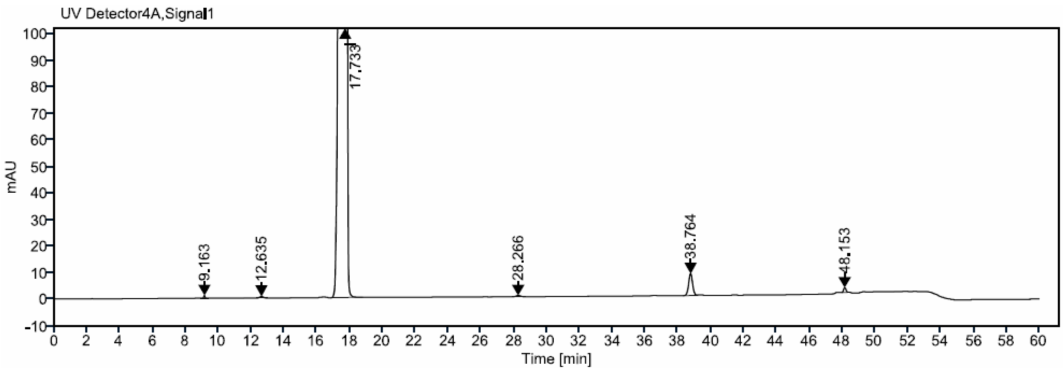

Signal: UV Detector4A,Signal1 Wavelength Ch1 225 nm

Peak Number	RT [min]	Area	Area%	Height	Theoretical Plates	Tail	Resolution
1	9.163	5.26	0.02	1.05	77903	1.05	
2	12.635	8.21	0.04	0.60	19090	1.11	13.97
3	17.733	21722.61	99.20	1092.17	17243	0.67	11.25
4	28.266	7.99	0.04	0.46	60308	1.16	21.05
5	38.764	135.67	0.62	8.20	121855	1.04	23.21
6	48.153	19.13	0.09	1.80	438106	1.17	25.54
	Sum	21898.87	100.00	1104.27			

SI-71 HPLC spectrum of ODV-13

No solids precipitated

SI-72. HPLC spectrum of ODV-14

琥珀酸去甲文拉法辛分析报告

**Instrument:**	ShimadzuLC08	**Project Name:**	Technology Research
**Manual Modified:**	None	**Operator:**	wujialing
**Sample name:**	Desvenlafaxine (O-WLFX-20210803-P-1)	**Inj. volume:**	10
**Location:**	1:4	**Last Modified By:**	wujialing
**Acq. method:**	Related substance method-11-S08.amx	**Injection Date:**	2021-08-03 18:00:13+08:00
**Acq.Method Version:**	2021-0720-0726-37551	**Modified Date:**	2021-08-04 08:22:22+08:00
**Pro.Method:**	*Related Substance.pmx	**Printied Date:**	2021-08-04 08:25:51+08:00
**Pro.Method Version:**	2021-0804-0021-55126	**Result Set Version:**	2021-0804-0022-32034

Work station:: Agilent OpenLAB CDS Software Version:2.3(Build 2.3.0.468)

Result Path: /Desvenlafaxine Succinate/Technology Research/Results/Related Substance/Desvenlafaxine Succinate/20210803-Desvenlafaxine purity detection (synthesis process optimization).rslt

Data file: Desvenlafaxine (O-WLFX-20210803-P-1).dx

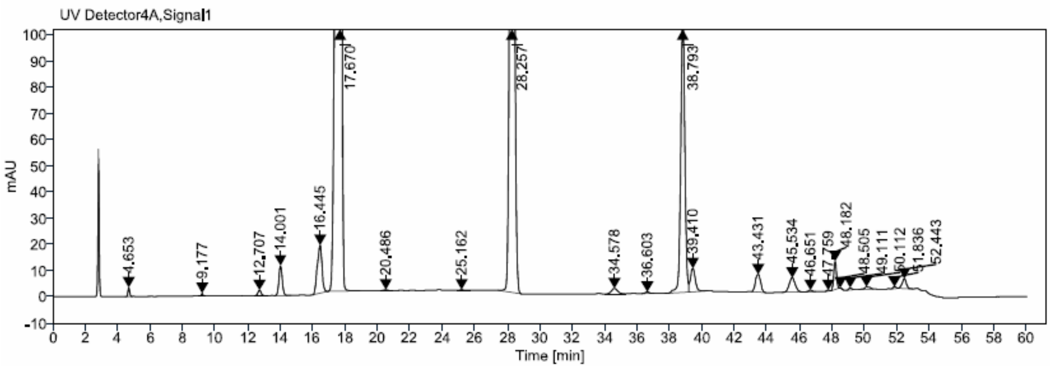

Signal: UV Detector4A,Signal1 Wavelength Ch1 225 nm

琥珀酸去甲文拉法辛分析报告

Peak Number	RT [min]	Area	Area%	Height	Theoretical Plates	Tail	Resolution
1	4.653	24.68	0.10	3.40	9381	1.16	
2	9.177	5.30	0.02	1.05	78795	1.03	28.01
3	12.707	26.67	0.11	2.02	20695	1.04	14.58
4	14.001	164.11	0.68	11.38	21297	1.10	3.51
5	16.445	368.84	1.52	18.30	13779	1.00	5.18
6	17.670	14810.47	61.09	817.49	20455	0.71	2.32
7	20.486	7.58	0.03	0.47	36146	1.07	6.09
8	25.162	4.67	0.02	0.33	70020	1.12	11.52
9	28.257	6220.71	25.66	353.41	58198	1.09	7.29
10	34.578	52.22	0.22	2.05	41910	1.09	11.05
11	36.603	10.16	0.04	0.61	107436	1.08	3.61
12	38.793	1824.28	7.52	103.73	115560	1.03	4.85
13	39.410	188.58	0.78	9.00	78497	1.46	1.21
14	43.431	149.38	0.62	6.79	91425	0.97	7.07
15	45.534	140.34	0.58	5.44	70680	1.08	3.34
16	46.651	8.73	0.04	0.55	198806	1.18	2.02
17	47.759	3.72	0.02	0.35	452166	1.08	3.15
18	48.182	124.13	0.51	10.68	377048	1.14	1.42
19	48.505	3.62	0.01	0.38	679516	1.04	1.18
20	49.111	9.91	0.04	0.77	321596	1.27	2.09
21	50.112	20.99	0.09	0.90	139188	1.09	2.26
22	51.836	9.28	0.04	0.69	309891	1.24	3.79
23	52.443	66.33	0.27	3.75	201351	0.89	1.44
	Sum	24244.68	100.00	1353.55			

SI-73. HPLC spectrum of ODV-15

琥珀酸去甲文拉法辛分析报告

**Instrument:**	ShimadzuLC08	**Project Name:**	Technology Research
**Manual Modified:**	None	**Operator:**	wujialing
**Sample name:**	Desvenlafaxine (O-WLFX-20210804-P-1)	**Inj. volume:**	10
**Location:**	1:2	**Last Modified By:**	wujialing
**Acq. method:**	Related substance method-11-S08.amx	**Injection Date:**	2021-08-04 13:53:16+08:00
**Acq.Method Version:**	2021-0720-0726-37551	**Modified Date:**	2021-08-04 17:00:12+08:00
**Pro.Method:**	*Related Substance.pmx	**Printied Date:**	2021-08-04 17:02:06+08:00
**Pro.Method Version:**	2021-0804-0859-46903	**Result Set Version:**	2021-0804-0900-18011

Work station:: Agilent OpenLAB CDS Software Version:2.3(Build 2.3.0.468)

Result Path: /Desvenlafaxine Succinate/Technology Research/Results/Related Substance/Desvenlafaxine Succinate/20210804-Desvenlafaxine purity detection (synthesis process optimization).rslt

Data file: Desvenlafaxine (O-WLFX-20210804-P-1).dx

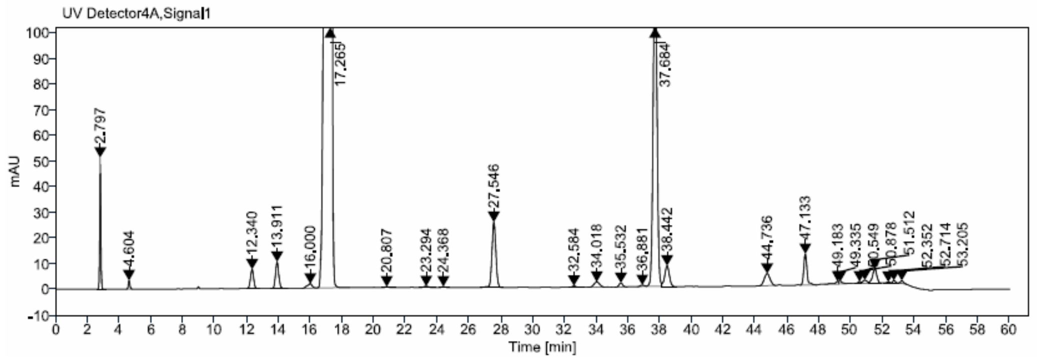

Signal: UV Detector4A,Signal1 Wavelength Ch1 225 nm

Peak Number	RT [min]	Area	Area%	Height	Theoretical Plates	Tail	Resolution
1	2.797	242.71	0.98	51.37	8444	1.02	
2	4.604	23.86	0.10	3.38	9695	1.17	11.71
3	12.340	101.75	0.41	7.48	18548	0.98	28.15
4	13.911	146.87	0.59	10.28	21403	1.09	4.23
5	16.000	36.05	0.15	1.72	14364	0.88	4.57
6	17.265	20854.00	84.18	1048.49	16564	0.66	2.36
7	20.807	6.86	0.03	0.42	38668	1.70	7.38
8	23.294	11.51	0.05	0.55	28443	1.04	5.10
9	24.368	5.18	0.02	0.36	67207	1.21	2.32
10	27.546	437.79	1.77	25.51	59204	1.04	7.67

琥珀酸去甲文拉法辛分析报告

Peak Number	RT [min]	Area	Area%	Height	Theoretical Plates	Tail	Resolution
11	32.584	8.21	0.03	0.54	103032	1.04	11.73
12	34.018	49.12	0.20	2.01	42756	1.04	2.69
13	35.532	24.84	0.10	1.53	105733	1.12	2.76
14	36.881	9.05	0.04	0.55	106950	1.07	3.04
15	37.684	2163.88	8.74	130.24	116193	1.03	1.80
16	38.442	161.65	0.65	8.09	86412	0.98	1.57
17	44.736	121.54	0.49	4.72	67615	1.08	10.39
18	47.133	169.15	0.68	12.21	294533	1.47	4.63
19	49.183	15.44	0.06	0.95	27776	0.56	2.68
20	49.335	14.63	0.06	0.96	29254	4.65	0.13
21	50.549	9.02	0.04	0.77	306290	0.85	1.60
22	50.878	24.00	0.10	1.28	157678	1.15	0.75
23	51.512	103.00	0.42	5.52	175928	0.88	1.26
24	52.352	6.35	0.03	0.43	260271	0.94	1.86
25	52.714	11.45	0.05	1.01	481648	1.04	1.01
26	53.205	14.12	0.06	1.12	402558	0.89	1.54
	Sum	24772.04	100.00	1321.49			

SI-74. HPLC spectrum of ODV-16

琥珀酸去甲文拉法辛分析报告

**Instrument:**	ShimadzuLC08	**Project Name:**	Technology Research
**Manual Modified:**	None	**Operator:**	wujialing
**Sample name:**	Desvenlafaxine (O-WLFX-20210804-P-2)	**Inj. volume:**	10
**Location:**	1:3	**Last Modified By:**	wujialing
**Acq. method:**	Related substance method-11-S08.amx	**Injection Date:**	2021-08-04 14:53:49+08:00
**Acq.Method Version:**	2021-0720-0726-37551	**Modified Date:**	2021-08-04 17:00:12+08:00
**Pro.Method:**	*Related Substance.pmx	**Printied Date:**	2021-08-04 17:02:56+08:00
**Pro.Method Version:**	2021-0804-0859-46903	**Result Set Version:**	2021-0804-0900-18011
**Work station::**	Agilent OpenLAB CDS Software Version:2.3(Build 2.3.0.468)		
**Result Path:**	/Desvenlafaxine Succinate/Technology Research/Results/Related Substance/Desvenlafaxine Succinate/20210804-Desvenlafaxine purity detection (synthesis process optimization).rslt		
**Data file:**	Desvenlafaxine (O-WLFX-20210804-P-2).dx		

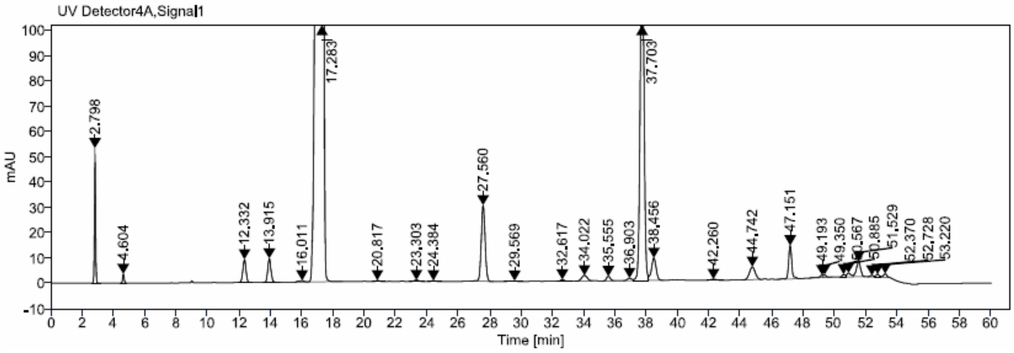

Signal: UV Detector4A,Signal1 Wavelength Ch1 225 nm

Peak Number	RT [min]	Area	Area%	Height	Theoretical Plates	Tail	Resolution
1	2.798	253.97	0.95	53.42	8410	1.00	
2	4.604	25.98	0.10	3.65	9618	1.18	11.65
3	12.332	126.19	0.47	9.09	17766	0.96	27.71
4	13.915	134.69	0.50	9.42	21390	1.10	4.22
5	16.011	14.63	0.05	0.64	10058	0.94	4.11
6	17.283	22580.67	84.02	1096.87	15750	0.65	2.14
7	20.817	8.01	0.03	0.51	41254	1.53	7.35
8	23.303	12.32	0.05	0.59	28361	1.05	5.16
9	24.384	5.88	0.02	0.40	64411	1.53	2.30
10	27.560	517.31	1.92	30.09	59061	1.05	7.58

琥珀酸去甲文拉法辛分析报告

Peak Number	RT [min]	Area	Area%	Height	Theoretical Plates	Tail	Resolution
11	29.569	6.40	0.02	0.21	19558	0.86	3.09
12	32.617	9.92	0.04	0.64	102957	1.05	4.87
13	34.022	54.06	0.20	2.20	42129	1.05	2.63
14	35.555	29.07	0.11	1.80	105856	1.11	2.79
15	36.903	18.45	0.07	0.96	74954	0.94	2.76
16	37.703	2343.82	8.72	140.37	116099	1.03	1.63
17	38.456	182.20	0.68	8.88	83115	1.00	1.54
18	42.260	9.88	0.04	0.44	86481	1.18	6.87
19	44.742	129.78	0.48	5.02	67188	1.08	3.92
20	47.151	184.29	0.69	13.23	295712	1.36	4.65
21	49.193	15.18	0.06	1.00	35053	0.62	2.92
22	49.350	13.15	0.05	0.99	30700	3.30	0.15
23	50.567	12.13	0.05	1.03	285672	0.85	1.62
24	50.885	29.51	0.11	1.58	151226	1.21	0.71
25	51.529	111.96	0.42	6.00	175832	0.89	1.27
26	52.370	8.32	0.03	0.53	204797	0.84	1.76
27	52.728	15.71	0.06	1.18	379485	1.18	0.89
28	53.220	20.55	0.08	1.41	323520	0.92	1.37
	Sum	26874.04	100.00	1392.14			

SI-75. HPLC spectrum of ODV-17

琥珀酸去甲文拉法辛分析报告

**Instrument:**	ShimadzuLC08	**Project Name:**	Technology Research
**Manual Modified:**	None	**Operator:**	wujialing
**Sample name:**	Desvenlafaxine (O-WLFX-20210804-P-3)	**Inj. volume:**	10
**Location:**	1:4	**Last Modified By:**	wujialing
**Acq. method:**	Related substance method-11-S08.amx	**Injection Date:**	2021-08-04 15:54:22+08:00
**Acq.Method Version:**	2021-0720-0726-37551	**Modified Date:**	2021-08-04 17:00:12+08:00
**Pro.Method:**	*Related Substance.pmx	**Printied Date:**	2021-08-04 17:03:18+08:00
**Pro.Method Version:**	2021-0804-0859-46903	**Result Set Version:**	2021-0804-0900-18011

Work station:: Agilent OpenLAB CDS Software Version:2.3(Build 2.3.0.468)

Result Path: /Desvenlafaxine Succinate/Technology Research/Results/Related Substance/Desvenlafaxine Succinate/20210804-Desvenlafaxine purity detection (synthesis process optimization).rslt

Data file: Desvenlafaxine (O-WLFX-20210804-P-3).dx

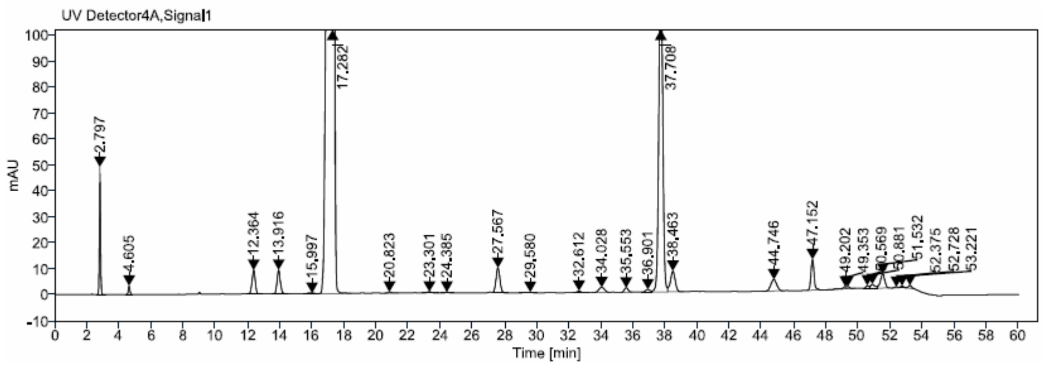

Signal: UV Detector4A,Signal1 Wavelength Ch1 225 nm

Peak Number	RT [min]	Area	Area%	Height	Theoretical Plates	Tail	Resolution
1	2.797	231.98	0.96	49.25	8448	1.03	
2	4.605	23.37	0.10	3.29	9646	1.18	11.69
3	12.364	125.45	0.52	9.15	18347	0.96	28.08
4	13.916	131.29	0.54	9.17	21379	1.09	4.16
5	15.997	5.11	0.02	0.21	10828	0.96	4.18
6	17.282	20652.16	85.19	1050.76	16873	0.67	2.24
7	20.823	7.87	0.03	0.49	40839	1.70	7.50
8	23.301	11.23	0.05	0.54	28476	1.05	5.14
9	24.385	5.26	0.02	0.36	64981	1.53	2.32
10	27.567	163.65	0.68	9.60	59082	1.04	7.61

琥珀酸去甲文拉法辛分析报告

Peak Number	RT [min]	Area	Area%	Height	Theoretical Plates	Tail	Resolution
11	29.580	6.10	0.03	0.21	21343	0.84	3.19
12	32.612	9.39	0.04	0.63	105429	1.03	5.00
13	34.028	48.47	0.20	1.97	42205	1.04	2.66
14	35.553	28.12	0.12	1.74	105621	1.11	2.77
15	36.901	19.24	0.08	1.01	78021	0.94	2.79
16	37.708	2126.13	8.77	127.14	115921	1.02	1.66
17	38.463	165.13	0.68	7.96	82227	0.99	1.54
18	44.746	115.12	0.47	4.44	67212	1.08	10.24
19	47.152	160.33	0.66	11.90	299038	1.21	4.65
20	49.202	19.13	0.08	0.94	32053	0.58	2.84
21	49.353	12.36	0.05	0.93	24443	3.02	0.13
22	50.569	12.50	0.05	1.05	279825	0.84	1.48
23	50.881	28.64	0.12	1.54	151976	1.25	0.69
24	51.532	102.42	0.42	5.48	175482	0.89	1.28
25	52.375	5.90	0.02	0.41	273270	0.94	1.89
26	52.728	9.40	0.04	0.86	518903	1.03	1.02
27	53.221	15.66	0.06	1.19	360258	0.85	1.52
	Sum	24241.42	100.00	1302.20			

SI-76. HPLC spectrum of ODV-18

琥珀酸去甲文拉法辛分析报告

**Instrument:**	ShimadzuLC08	**Project Name:**	Technology Research
**Manual Modified:**	None	**Operator:**	wujialing
**Sample name:**	Desvenlafaxine (O-WLFX-20210807-P)	**Inj. volume:**	10
**Location:**	1:2	**Last Modified By:**	wujialing
**Acq. method:**	Related substance method-11-S08.amx	**Injection Date:**	2021-08-09 15:56:00+08:00
**Acq.Method Version:**	2021-0720-0726-37551	**Modified Date:**	2021-08-10 08:14:38+08:00
**Pro.Method:**	*Related Substance.pmx	**Printied Date:**	2021-08-10 08:19:19+08:00
**Pro.Method Version:**	2021-0810-0010-42579	**Result Set Version:**	2021-0810-0015-18776

Work station:: Agilent OpenLAB CDS Software Version:2.3(Build 2.3.0.468)

Result Path: /Desvenlafaxine Succinate/Technology Research/Results/Related Substance/Desvenlafaxine Succinate/20210809-Desvenlafaxine purity detection (synthesis process optimization).rslt

Data file: Desvenlafaxine (O-WLFX-20210807-P).dx

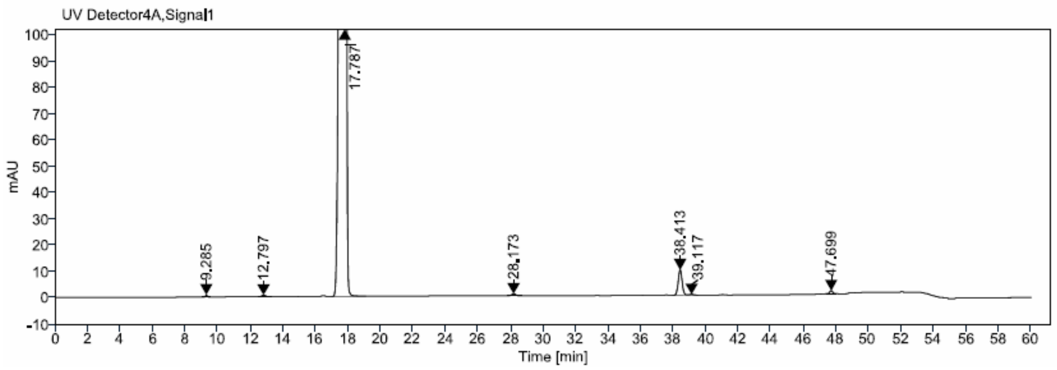

Signal: UV Detector4A,Signal1 Wavelength Ch1 225 nm

Peak Number	RT [min]	Area	Area%	Height	Theoretical Plates	Tail	Resolution
1	9.285	4.47	0.02	0.81	69451	1.05	
2	12.797	9.39	0.05	0.62	16477	1.21	13.01
3	17.787	20582.39	99.02	1112.50	19362	0.69	10.97
4	28.173	13.70	0.07	0.81	61903	1.06	21.54
5	38.413	157.31	0.76	9.59	122071	1.04	22.94
6	39.117	5.40	0.03	0.31	109599	0.94	1.55
7	47.699	13.02	0.06	1.15	384514	1.17	21.99
	Sum	20785.67	100.00	1125.81			

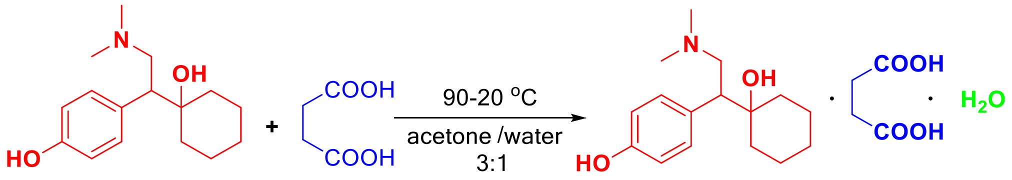

ODV DVS

SI-77. HPLC spectrum of DVS-1

琥珀酸去甲文拉法辛分析报告

Instrument: ShimadzuLC08

Project Name: Technology Research

Manual Modified: None

Operator: wujialing

Sample name: Desvenlafaxine (O-WLFX-20210915-P-hps-1)

Inj. volume: 10

Location: 1:2

Last Modified By: wujialing

Acq. method: Related substance method-11-S08.amx

Injection Date: 2021-09-16 18:16:24+08:00

Acq.Method Version: 2021-0720-0726-37551

Modified Date: 2021-09-17 08:16:14+08:00

Pro.Method: *Related Substance.pmx

Printied Date: 2021-09-17 08:18:03+08:00

Pro.Method Version: 2021-0917-0013-23050

Result Set Version: 2021-0917-0016-18845

Work station:: Agilent OpenLAB CDS Software Version:2.3(Build 2.3.0.468)

Result Path: /Desvenlafaxine Succinate/Technology Research/Results/Related Substance/Desvenlafaxine Succinate/20210916-Desvenlafaxine Succinate purity detection (synthesis process optimization).rslt

Data file: Desvenlafaxine (O-WLFX-20210915-P-hps-1).dx

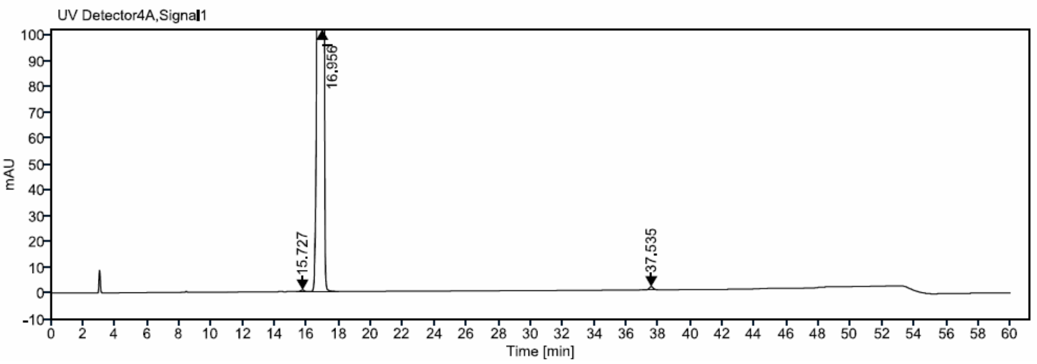

Signal: UV Detector4A,Signal1 Wavelength Ch1 225 nm

Peak Number	RT [min]	Area	Area%	Height	Theoretical Plates	Tail	Resolution
1	15.727	10.00	0.07	0.63	22498	1.07	
2	16.956	13909.00	99.79	816.25	21073	0.72	2.77
3	37.535	19.47	0.14	1.17	115851	1.05	45.32
	Sum	13938.47	100.00	818.05			

SI-78. HPLC spectrum of DVS-2

琥珀酸去甲文拉法辛分析报告

**Instrument:**	ShimadzuLC08	**Project Name:**	Technology Research
**Manual Modified:**	None	**Operator:**	wujialing
**Sample name:**	Desvenlafaxine (O-WLFX-20210915-P-hps-2)	**Inj. volume:**	10
**Location:**	1:3	**Last Modified By:**	wujialing
**Acq. method:**	Related substance method-11-S08.amx	**Injection Date:**	2021-09-16 19:16:58+08:00
**Acq.Method Version:**	2021-0720-0726-37551	**Modified Date:**	2021-09-17 08:16:14+08:00
**Pro.Method:**	*Related Substance.pmx	**Printied Date:**	2021-09-17 08:18:55+08:00
**Pro.Method Version:**	2021-0917-0013-23050	**Result Set Version:**	2021-0917-0016-18845

Work station:: Agilent OpenLAB CDS Software Version:2.3(Build 2.3.0.468)

Result Path: /Desvenlafaxine Succinate/Technology Research/Results/Related Substance/Desvenlafaxine Succinate/20210916-Desvenlafaxine Succinate purity detection (synthesis process optimization).rslt

Data file: Desvenlafaxine (O-WLFX-20210915-P-hps-2).dx

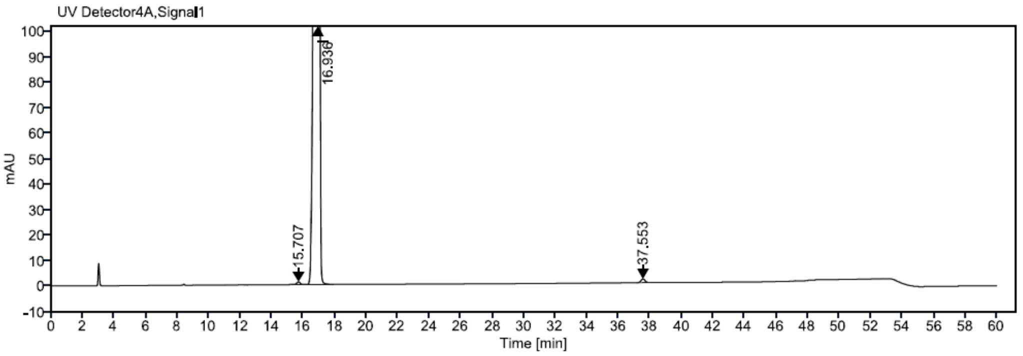

Signal: UV Detector4A,Signal1 Wavelength Ch1 225 nm

Peak Number	RT [min]	Area	Area%	Height	Theoretical Plates	Tail	Resolution
1	15.707	15.04	0.11	0.96	22446	1.04	
2	16.936	13792.30	99.72	811.49	21110	0.71	2.78
3	37.553	23.48	0.17	1.43	115539	1.05	45.40
	Sum	13830.82	100.00	813.88			

SI-79. HPLC spectrum of DVS-3

琥珀酸去甲文拉法辛分析报告

**Instrument:**	ShimadzuLC08	**Project Name:**	Technology Research
**Manual Modified:**	None	**Operator:**	wujialing
**Sample name:**	Desvenlafaxine (O-WLFX-20210915-P-hps-3)	**Inj. volume:**	10
**Location:**	1:4	**Last Modified By:**	wujialing
**Acq. method:**	Related substance method-11-S08.amx	**Injection Date:**	2021-09-16 20:17:33+08:00
**Acq.Method Version:**	2021-0720-0726-37551	**Modified Date:**	2021-09-17 08:16:14+08:00
**Pro.Method:**	*Related Substance.pmx	**Printied Date:**	2021-09-17 08:19:15+08:00
**Pro.Method Version:**	2021-0917-0013-23050	**Result Set Version:**	2021-0917-0016-18845

Work station:: Agilent OpenLAB CDS Software Version:2.3(Build 2.3.0.468)

Result Path: /Desvenlafaxine Succinate/Technology Research/Results/Related Substance/Desvenlafaxine Succinate/20210916-Desvenlafaxine Succinate purity detection (synthesis process optimization).rslt

Data file: Desvenlafaxine (O-WLFX-20210915-P-hps-3).dx

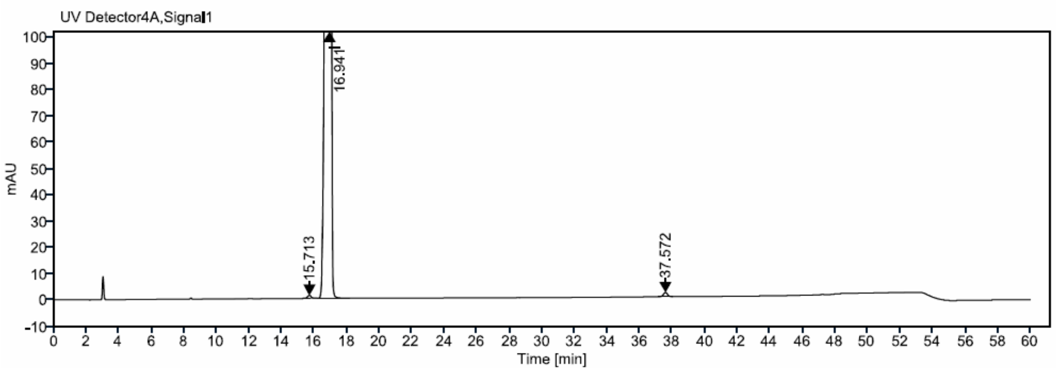

Signal: UV Detector4A,Signal1 Wavelength Ch1 225 nm

Peak Number	RT [min]	Area	Area%	Height	Theoretical Plates	Tail	Resolution
1	15.713	17.53	0.13	1.12	22319	1.02	
2	16.941	13916.19	99.69	817.62	21032	0.71	2.77
3	37.572	26.39	0.19	1.61	116372	1.04	45.45
	Sum	13960.12	100.00	820.34			

SI-80. HPLC spectrum of DVS-4

琥珀酸去甲文拉法辛分析报告

**Instrument:**	ShimadzuLC08	**Project Name:**	Technology Research
**Manual Modified:**	None	**Operator:**	wujialing
**Sample name:**	Desvenlafaxine (O-WLFX-20210918-Ph-1)	**Inj. volume:**	10
**Location:**	1:2	**Last Modified By:**	wujialing
**Acq. method:**	Related substance method-11-S08.amx	**Injection Date:**	2021-09-22 13:58:22+08:00
**Acq.Method Version:**	2021-0720-0726-37551	**Modified Date:**	2021-09-22 17:08:58+08:00
**Pro.Method:**	*Related Substance.pmx	**Printied Date:**	2021-09-22 17:11:15+08:00
**Pro.Method Version:**	2021-0922-0908-15189	**Result Set Version:**	2021-0922-0909-16085

Work station:: Agilent OpenLAB CDS Software Version:2.3(Build 2.3.0.468)

Result Path: /Desvenlafaxine Succinate/Technology Research/Results/Related Substance/Desvenlafaxine Succinate/20210922-Desvenlafaxine Succinate purity detection (synthesis process optimization).rslt

Data file: Desvenlafaxine (O-WLFX-20210918-Ph-1).dx

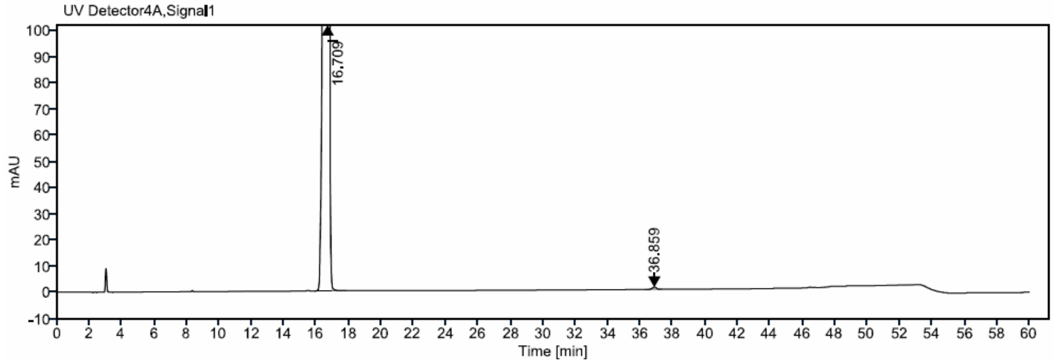

Signal:	UV Detector4A,Signal1		Wavelength Ch1	225 nm			
Peak Number	RT [min]	Area	Area%	Height	Theoretical Plates	Tail	Resolution
1	16.709	14409.72	99.90	833.93	20010	0.71	
2	36.859	15.06	0.10	0.87	103600	1.03	43.30
	Sum	14424.78	100.00	834.80			

SI-81. HPLC spectrum of DVS-5

琥珀酸去甲文拉法辛分析报告

**Instrument:**	ShimadzuLC08	**Project Name:**	Technology Research
**Manual Modified:**	None	**Operator:**	wujialing
**Sample name:**	Desvenlafaxine (O-WLFX-20210918-Ph-2)	**Inj. volume:**	10
**Location:**	1:3	**Last Modified By:**	wujialing
**Acq. method:**	Related substance method-11-S08.amx	**Injection Date:**	2021-09-22 14:58:56+08:00
**Acq.Method Version:**	2021-0720-0726-37551	**Modified Date:**	2021-09-22 17:08:58+08:00
**Pro.Method:**	*Related Substance.pmx	**Printied Date:**	2021-09-22 17:12:17+08:00
**Pro.Method Version:**	2021-0922-0908-15189	**Result Set Version:**	2021-0922-0909-16085
**Work station::**	Agilent OpenLAB CDS Software Version:2.3(Build 2.3.0.468)		
**Result Path:**	/Desvenlafaxine Succinate/Technology Research/Results/Related Substance/Desvenlafaxine Succinate/20210922-Desvenlafaxine Succinate purity detection (synthesis process optimization).rslt		
**Data file:**	Desvenlafaxine (O-WLFX-20210918-Ph-2).dx		

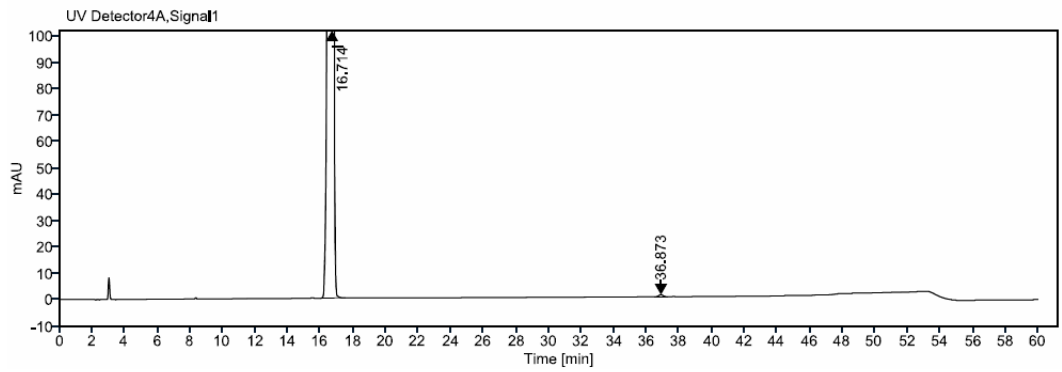

Signal:	UV Detector4A,Signal1	Wavelength Ch1	225 nm

Peak Number	RT [min]	Area	Area%	Height	Theoretical Plates	Tail	Resolution
1	16.714	13235.34	99.90	785.99	21010	0.72	
2	36.873	13.15	0.10	0.77	105326	1.02	44.03
	Sum	13248.48	100.00	786.76			

SI-82. HPLC spectrum of DVS-6

琥珀酸去甲文拉法辛分析报告

**Instrument:**	ShimadzuLC08	**Project Name:**	Technology Research
**Manual Modified:**	None	**Operator:**	wujialing
**Sample name:**	Desvenlafaxine (O-WLFX-20210918-Ph-3)	**Inj. volume:**	10
**Location:**	1:4	**Last Modified By:**	wujialing
**Acq. method:**	Related substance method-11-S08.amx	**Injection Date:**	2021-09-22 15:59:30+08:00
**Acq.Method Version:**	2021-0720-0726-37551	**Modified Date:**	2021-09-22 17:08:58+08:00
**Pro.Method:**	*Related Substance.pmx	**Printied Date:**	2021-09-22 17:12:39+08:00
**Pro.Method Version:**	2021-0922-0908-15189	**Result Set Version:**	2021-0922-0909-16085

Work station:: Agilent OpenLAB CDS Software Version:2.3(Build 2.3.0.468)

Result Path: /Desvenlafaxine Succinate/Technology Research/Results/Related Substance/Desvenlafaxine Succinate/20210922-Desvenlafaxine Succinate purity detection (synthesis process optimization).rslt

Data file: Desvenlafaxine (O-WLFX-20210918-Ph-3).dx

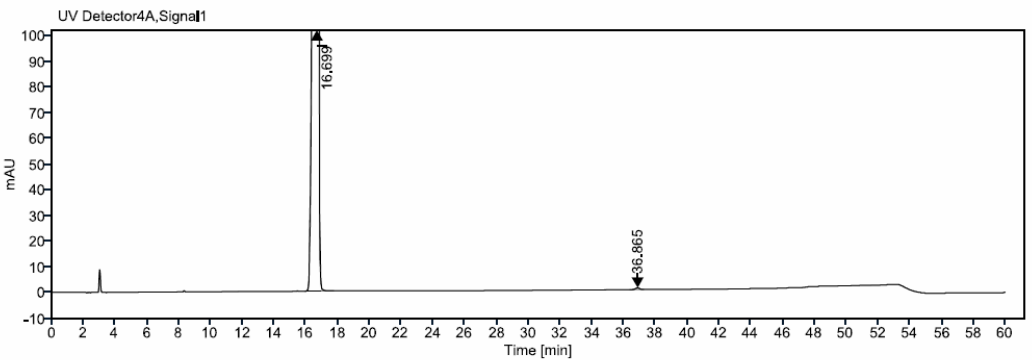

Signal: UV Detector4A,Signal1 Wavelength Ch1 225 nm

Peak Number	RT [min]	Area	Area%	Height	Theoretical Plates	Tail	Resolution
1	16.699	14093.85	99.92	820.72	20211	0.72	
2	36.865	11.31	0.08	0.63	97092	1.00	42.77
	Sum	14105.16	100.00	821.35			

SI-83. HPLC spectrum of DVS-7

琥珀酸去甲文拉法辛分析报告

**Instrument:**	ShimadzuLC08	**Project Name:**	Technology Research
**Manual Modified:**	None	**Operator:**	wujialing
**Sample name:**	Desvenlafaxine Succinate (O-WLFX-20210922-Ph-1)	**Inj. volume:**	10
**Location:**	1:2	**Last Modified By:**	wujialing
**Acq. method:**	Related substance method-11-S08.amx	**Injection Date:**	2021-09-23 11:22:27+08:00
**Acq.Method Version:**	2021-0720-0726-37551	**Modified Date:**	2021-09-23 14:26:43+08:00
**Pro.Method:**	*Related Substance.pmx	**Printied Date:**	2021-09-23 14:28:29+08:00
**Pro.Method Version:**	2021-0923-0626-25918	**Result Set Version:**	2021-0923-0627-01100

Work station:: Agilent OpenLAB CDS Software Version:2.3(Build 2.3.0.468)

Result Path: /Desvenlafaxine Succinate/Technology Research/Results/Related Substance/Desvenlafaxine Succinate/20210923-Desvenlafaxine Succinate purity detection (synthesis process optimization).rslt

Data file: Desvenlafaxine Succinate (O-WLFX-20210922-Ph-1).dx

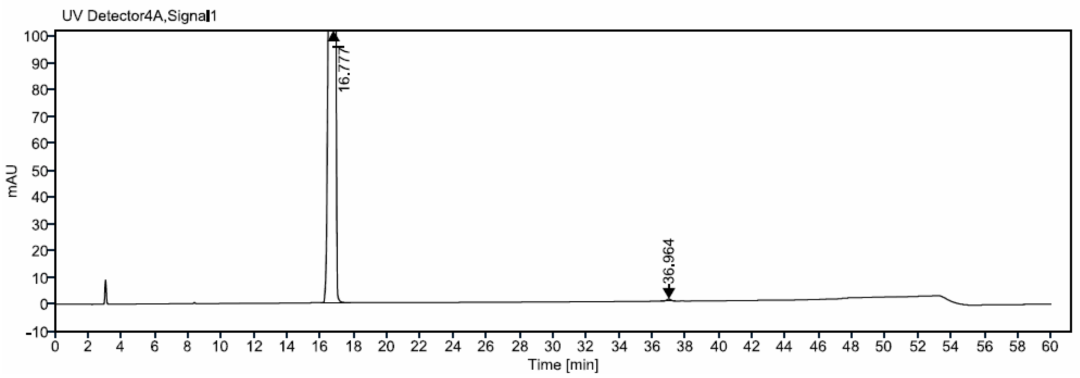

Signal: UV Detector4A,Signal1 Wavelength Ch1 225 nm

Peak Number	RT [min]	Area	Area%	Height	Theoretical Plates	Tail	Resolution
1	16.777	14380.77	99.90	834.85	20273	0.71	
2	36.964	13.91	0.10	0.82	107633	1.03	43.79
	Sum	14394.68	100.00	835.67			

SI-84. HPLC spctrum of DVS-8

琥珀酸去甲文拉法辛分析报告

**Instrument:**	ShimadzuLC08	**Project Name:**	Technology Research
**Manual Modified:**	None	**Operator:**	wujialing
**Sample name:**	Desvenlafaxine Succinate (O-WLFX-20210922-Ph-2)	**Inj. volume:**	10
**Location:**	1:3	**Last Modified By:**	wujialing
**Acq. method:**	Related substance method-11-S08.amx	**Injection Date:**	2021-09-23 12:23:00+08:00
**Acq.Method Version:**	2021-0720-0726-37551	**Modified Date:**	2021-09-23 14:26:43+08:00
**Pro.Method:**	*Related Substance.pmx	**Printied Date:**	2021-09-23 14:29:23+08:00
**Pro.Method Version:**	2021-0923-0626-25918	**Result Set Version:**	2021-0923-0627-01100

Work station:: Agilent OpenLAB CDS Software Version:2.3(Build 2.3.0.468)

Result Path: /Desvenlafaxine Succinate/Technology Research/Results/Related Substance/Desvenlafaxine Succinate/20210923-Desvenlafaxine Succinate purity detection (synthesis process optimization).rslt

Data file: Desvenlafaxine Succinate (O-WLFX-20210922-Ph-2).dx

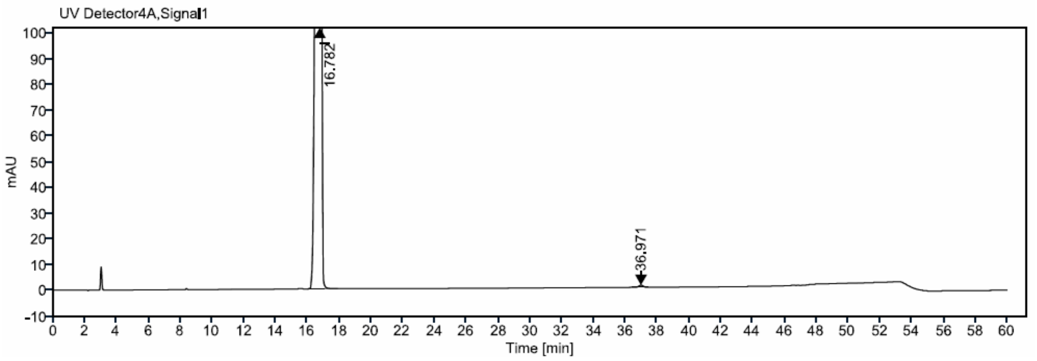

Signal: UV Detector4A,Signal1 Wavelength Ch1 225 nm

Peak Number	RT [min]	Area	Area%	Height	Theoretical Plates	Tail	Resolution
1	16.782	14369.25	99.90	836.46	20374	0.71	
2	36.971	14.71	0.10	0.86	106603	1.01	43.74
	Sum	14383.95	100.00	837.32			

SI-85. HPLC spectrum of DVS-9

琥珀酸去甲文拉法辛分析报告

**Instrument:**	ShimadzuLC08	**Project Name:**	Technology Research
**Manual Modified:**	None	**Operator:**	wujialing
**Sample name:**	Desvenlafaxine Succinate (O-WLFX-20210922-Ph-3)	**Inj. volume:**	10
**Location:**	1:4	**Last Modified By:**	wujialing
**Acq. method:**	Related substance method-11-S08.amx	**Injection Date:**	2021-09-23 13:23:35+08:00
**Acq.Method Version:**	2021-0720-0726-37551	**Modified Date:**	2021-09-23 14:26:43+08:00
**Pro.Method:**	*Related Substance.pmx	**Printied Date:**	2021-09-23 14:29:49+08:00
**Pro.Method Version:**	2021-0923-0626-25918	**Result Set Version:**	2021-0923-0627-01100

Work station:: Agilent OpenLAB CDS Software Version:2.3(Build 2.3.0.468)

Result Path: /Desvenlafaxine Succinate/Technology Research/Results/Related Substance/Desvenlafaxine Succinate/20210923-Desvenlafaxine Succinate purity detection (synthesis process optimization).rslt

Data file: Desvenlafaxine Succinate (O-WLFX-20210922-Ph-3).dx

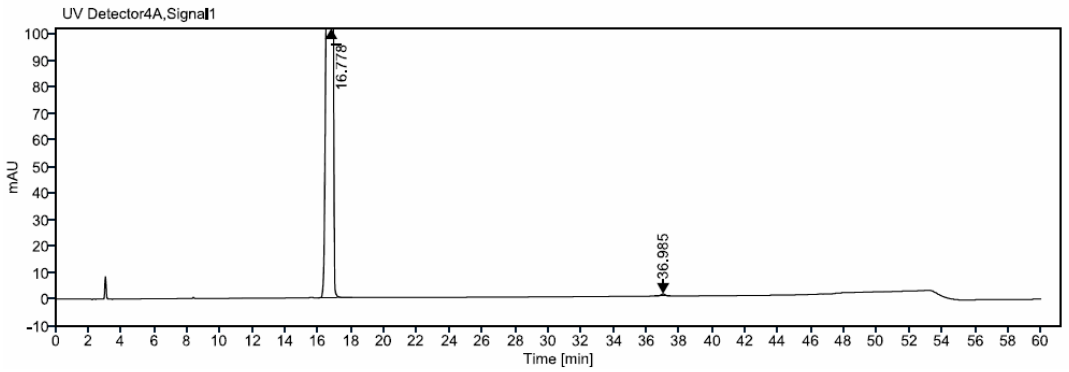

Signal: UV Detector4A,Signal1 Wavelength Ch1 225 nm

Peak Number	RT [min]	Area	Area%	Height	Theoretical Plates	Tail	Resolution
1	16.778	13396.11	99.89	797.52	21241	0.72	
2	36.985	14.25	0.11	0.84	107089	1.03	44.29
	Sum	13410.35	100.00	798.36			

SI-86. HPLC spectrum of DVS-10

琥珀酸去甲文拉法辛分析报告

**Instrument:**	ShimadzuLC08	**Project Name:**	Technology Research
**Manual Modified:**	None	**Operator:**	wujialing
**Sample name:**	Desvenlafaxine Succinate (O-WLFX-20210924-Ph-1)	**Inj. volume:**	10
**Location:**	1:2	**Last Modified By:**	wujialing
**Acq. method:**	Related substance method-11-S08.amx	**Injection Date:**	2021-09-24 18:22:46+08:00
**Acq.Method Version:**	2021-0720-0726-37551	**Modified Date:**	2021-09-26 07:43:41+08:00
**Pro.Method:**	*Related Substance.pmx	**Printied Date:**	2021-09-26 08:29:12+08:00
**Pro.Method Version:**	2021-0925-2343-27389	**Result Set Version:**	2021-0926-0027-30142

Work station:: Agilent OpenLAB CDS Software Version:2.3(Build 2.3.0.468)

Result Path: /Desvenlafaxine Succinate/Technology Research/Results/Related Substance/Desvenlafaxine Succinate/20210924-Desvenlafaxine Succinate purity detection (synthesis process optimization).rslt

Data file: Desvenlafaxine Succinate (O-WLFX-20210924-Ph-1).dx

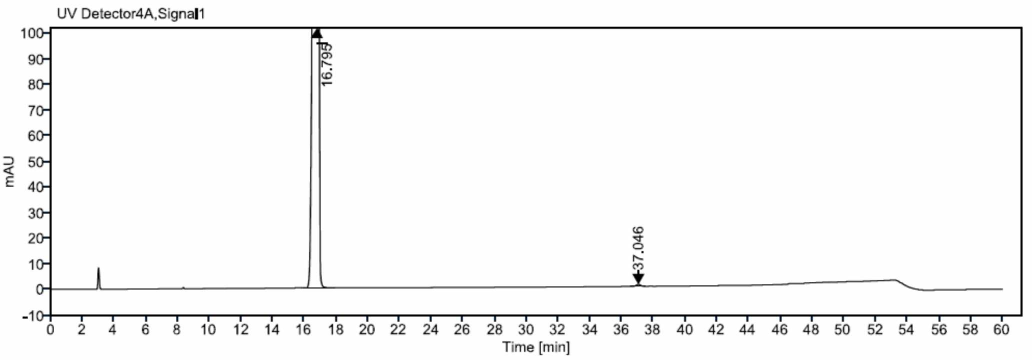

Signal:	UV Detector4A,Signal1	Wavelength Ch1	225 nm				
Peak Number	RT [min]	Area	Area%	Height	Theoretical Plates	Tail	Resolution
1	16.795	13551.95	99.90	798.87	20880	0.73	
2	37.046	13.63	0.10	0.80	106238	1.06	44.04
	Sum	13565.59	100.00	799.67			

SI-87. HPLC spectrum of DVS-11

琥珀酸去甲文拉法辛分析报告

**Instrument:**	ShimadzuLC08	**Project Name:**	Technology Research
**Manual Modified:**	None	**Operator:**	wujialing
**Sample name:**	Desvenlafaxine Succinate (O-WLFX-20210924-Ph-2)	**Inj. volume:**	10
**Location:**	1:3	**Last Modified By:**	wujialing
**Acq. method:**	Related substance method-11-S08.amx	**Injection Date:**	2021-09-24 19:23:19+08:00
**Acq.Method Version:**	2021-0720-0726-37551	**Modified Date:**	2021-09-26 07:43:41+08:00
**Pro.Method:**	*Related Substance.pmx	**Printied Date:**	2021-09-26 08:29:58+08:00
**Pro.Method Version:**	2021-0925-2343-27389	**Result Set Version:**	2021-0926-0027-30142

Work station:: Agilent OpenLAB CDS Software Version:2.3(Build 2.3.0.468)

Result Path: /Desvenlafaxine Succinate/Technology Research/Results/Related Substance/Desvenlafaxine Succinate/20210924-Desvenlafaxine Succinate purity detection (synthesis process optimization).rslt

Data file: Desvenlafaxine Succinate (O-WLFX-20210924-Ph-2).dx

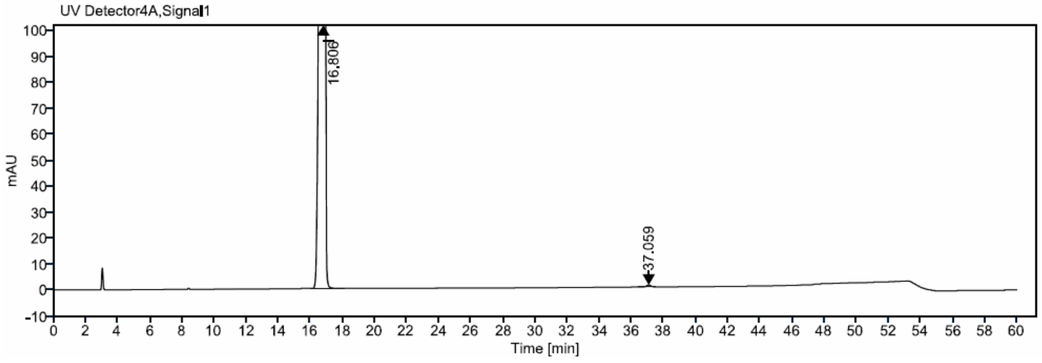

Signal: UV Detector4A,Signal1 Wavelength Ch1 225 nm

Peak Number	RT [min]	Area	Area%	Height	Theoretical Plates	Tail	Resolution
1	16.806	13600.82	99.90	800.78	20858	0.73	
2	37.059	13.34	0.10	0.78	106547	1.04	44.05
	Sum	13614.16	100.00	801.56			

SI-88. HPLC spectrum of DVS-12

琥珀酸去甲文拉法辛分析报告

**Instrument:**	ShimadzuLC08	**Project Name:**	Technology Research
**Manual Modified:**	None	**Operator:**	wujialing
**Sample name:**	Desvenlafaxine Succinate (O-WLFX-20210924-Ph-3)	**Inj. volume:**	10
**Location:**	1:4	**Last Modified By:**	wujialing
**Acq. method:**	Related substance method-11-S08.amx	**Injection Date:**	2021-09-24 20:23:53+08:00
**Acq.Method Version:**	2021-0720-0726-37551	**Modified Date:**	2021-09-26 07:43:41+08:00
**Pro.Method:**	*Related Substance.pmx	**Printied Date:**	2021-09-26 08:30:19+08:00
**Pro.Method Version:**	2021-0925-2343-27389	**Result Set Version:**	2021-0926-0027-30142

Work station:: Agilent OpenLAB CDS Software Version:2.3(Build 2.3.0.468)

Result Path: /Desvenlafaxine Succinate/Technology Research/Results/Related Substance/Desvenlafaxine Succinate/20210924-Desvenlafaxine Succinate purity detection (synthesis process optimization).rslt

Data file: Desvenlafaxine Succinate (O-WLFX-20210924-Ph-3).dx

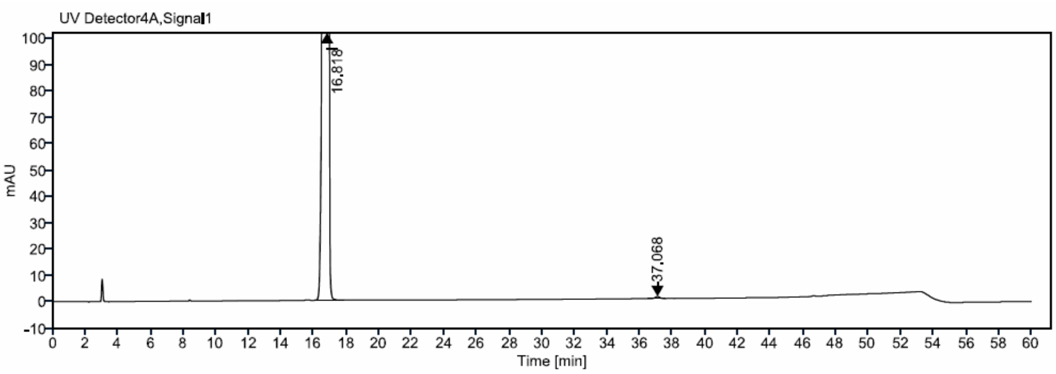

Signal: UV Detector4A,Signal1 Wavelength Ch1 225 nm

Peak Number	RT [min]	Area	Area%	Height	Theoretical Plates	Tail	Resolution
1	16.818	13673.61	99.90	806.05	20938	0.72	
2	37.068	13.65	0.10	0.79	106751	1.03	44.08
	Sum	13687.26	100.00	806.84			

SI-89. HPLC spectrum of DVS-13

琥珀酸去甲文拉法辛分析报告

**Instrument:**	ShimadzuLC08	**Project Name:**	Technology Research
**Manual Modified:**	None	**Operator:**	wujialing
**Sample name:**	Desvenlafaxine Succinate (O-WLFX-20210926-Ph-1)	**Inj. volume:**	10
**Location:**	1:2	**Last Modified By:**	wujialing
**Acq. method:**	Related substance method-11-S08.amx	**Injection Date:**	2021-09-26 19:40:13+08:00
**Acq.Method Version:**	2021-0720-0726-37551	**Modified Date:**	2021-09-27 08:08:04+08:00
**Pro.Method:**	*Related Substance.pmx	**Printied Date:**	2021-09-27 08:10:29+08:00
**Pro.Method Version:**	2021-0927-0007-51686	**Result Set Version:**	2021-0927-0008-37178

Work station:: Agilent OpenLAB CDS Software Version:2.3(Build 2.3.0.468)

Result Path: /Desvenlafaxine Succinate/Technology Research/Results/Related Substance/Desvenlafaxine Succinate/20210926-Desvenlafaxine Succinate purity detection (synthesis process optimization).rslt

Data file: Desvenlafaxine Succinate (O-WLFX-20210926-Ph-1).dx

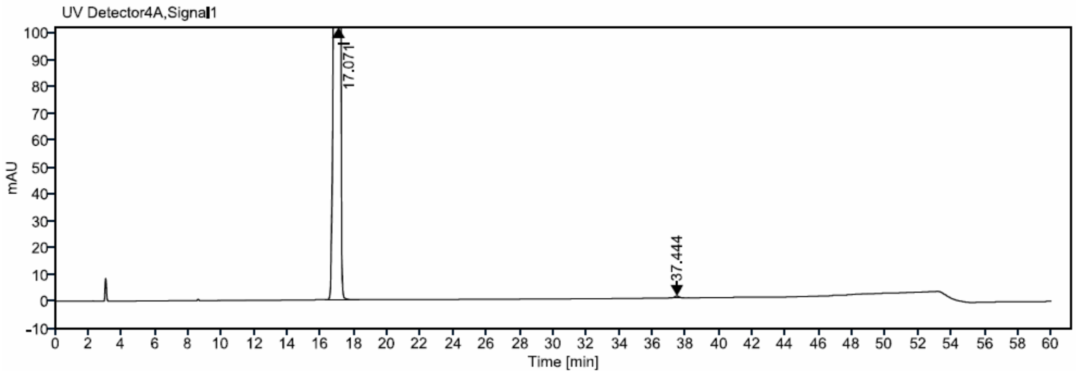

Signal: UV Detector4A,Signal1 Wavelength Ch1 225 nm

Peak Number	RT [min]	Area	Area%	Height	Theoretical Plates	Tail	Resolution
1	17.071	13835.74	99.91	811.87	21371	0.73	
2	37.444	12.66	0.09	0.74	107458	1.09	44.10
	Sum	13848.40	100.00	812.61			

SI-90. HPLC spectrum of DVS-14

琥珀酸去甲文拉法辛分析报告

**Instrument:**	ShimadzuLC08	**Project Name:**	Technology Research
**Manual Modified:**	None	**Operator:**	wujialing
**Sample name:**	Desvenlafaxine Succinate (O-WLFX-20210926-Ph-2)	**Inj. volume:**	10
**Location:**	1:3	**Last Modified By:**	wujialing
**Acq. method:**	Related substance method-11-S08.amx	**Injection Date:**	2021-09-26 20:40:46+08:00
**Acq.Method Version:**	2021-0720-0726-37551	**Modified Date:**	2021-09-27 08:08:04+08:00
**Pro.Method:**	*Related Substance.pmx	**Printied Date:**	2021-09-27 08:11:05+08:00
**Pro.Method Version:**	2021-0927-0007-51686	**Result Set Version:**	2021-0927-0008-37178

Work station:: Agilent OpenLAB CDS Software Version:2.3(Build 2.3.0.468)

Result Path: /Desvenlafaxine Succinate/Technology Research/Results/Related Substance/Desvenlafaxine Succinate/20210926-Desvenlafaxine Succinate purity detection (synthesis process optimization).rslt

Data file: Desvenlafaxine Succinate (O-WLFX-20210926-Ph-2).dx

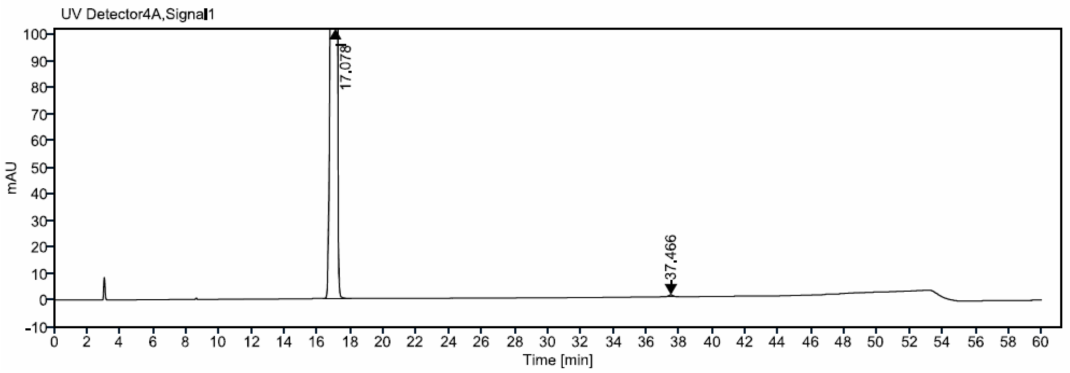

Signal: UV Detector4A,Signal1 Wavelength Ch1 225 nm

Peak Number	RT [min]	Area	Area%	Height	Theoretical Plates	Tail	Resolution
1	17.078	13710.39	99.90	807.40	21519	0.73	
2	37.466	13.04	0.10	0.76	108374	1.05	44.28
	Sum	13723.43	100.00	808.16			

SI-91. HPLC spectrum of DVS-15

琥珀酸去甲文拉法辛分析报告

**Instrument:**	ShimadzuLC08	**Project Name:**	Technology Research
**Manual Modified:**	None	**Operator:**	wujialing
**Sample name:**	Desvenlafaxine Succinate (O-WLFX-20210926-Ph-3)	**Inj. volume:**	10
**Location:**	1:4	**Last Modified By:**	wujialing
**Acq. method:**	Related substance method-11-S08.amx	**Injection Date:**	2021-09-26 21:41:21+08:00
**Acq.Method Version:**	2021-0720-0726-37551	**Modified Date:**	2021-09-27 08:08:04+08:00
**Pro.Method:**	*Related Substance.pmx	**Printied Date:**	2021-09-27 08:11:24+08:00
**Pro.Method Version:**	2021-0927-0007-51686	**Result Set Version:**	2021-0927-0008-37178

Work station:: Agilent OpenLAB CDS Software Version:2.3(Build 2.3.0.468)

Result Path: /Desvenlafaxine Succinate/Technology Research/Results/Related Substance/Desvenlafaxine Succinate/20210926-Desvenlafaxine Succinate purity detection (synthesis process optimization).rslt

Data file: Desvenlafaxine Succinate (O-WLFX-20210926-Ph-3).dx

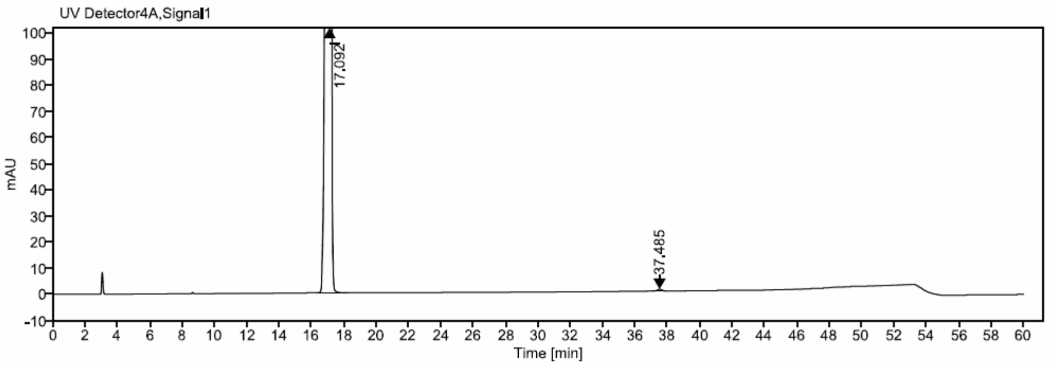

Signal: UV Detector4A,Signal1 Wavelength Ch1 225 nm

Peak Number	RT [min]	Area	Area%	Height	Theoretical Plates	Tail	Resolution
1	17.092	13627.42	99.91	806.04	21742	0.73	
2	37.485	12.41	0.09	0.72	107831	1.04	44.32
	Sum	13639.83	100.00	806.76			

SI-92 HPLC spectrum of DVS-16

琥珀酸去甲文拉法辛分析报告

**Instrument:**	ShimadzuLC08	**Project Name:**	Technology Research
**Manual Modified:**	None	**Operator:**	wujialing
**Sample name:**	Desvenlafaxine Succinate (O-WLFX-20210927-Ph-1)	**Inj. volume:**	10
**Location:**	1:3	**Last Modified By:**	wujialing
**Acq. method:**	Related substance method-11-S08.amx	**Injection Date:**	2021-09-27 19:56:16+08:00
**Acq.Method Version:**	2021-0720-0726-37551	**Modified Date:**	2021-09-28 08:27:27+08:00
**Pro.Method:**	*Related Substance.pmx	**Printied Date:**	2021-09-28 08:30:17+08:00
**Pro.Method Version:**	2021-0928-0027-14507	**Result Set Version:**	2021-0928-0027-48807

Work station:: Agilent OpenLAB CDS Software Version:2.3(Build 2.3.0.468)

Result Path: /Desvenlafaxine Succinate/Technology Research/Results/Related Substance/Desvenlafaxine Succinate/20210927-Desvenlafaxine Succinate purity detection (synthesis process optimization).rslt

Data file: Desvenlafaxine Succinate (O-WLFX-20210927-Ph-1).dx

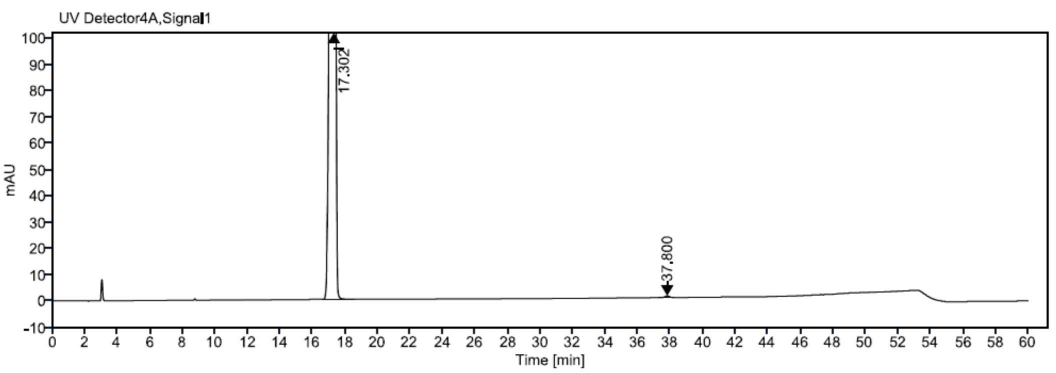

Signal: UV Detector4A,Signal1 Wavelength Ch1 225 nm

Peak Number	RT [min]	Area	Area%	Height	Theoretical Plates	Tail	Resolution
1	17.302	13273.74	99.92	794.40	22820	0.74	
2	37.800	11.27	0.08	0.65	109117	1.05	44.76
	Sum	13285.01	100.00	795.05			

SI-93. HPLC spectrum of DVS-17

琥珀酸去甲文拉法辛分析报告

**Instrument:**	ShimadzuLC08	**Project Name:**	Technology Research
**Manual Modified:**	None	**Operator:**	wujialing
**Sample name:**	Desvenlafaxine Succinate (O-WLFX-20210927-Ph-2)	**Inj. volume:**	10
**Location:**	1:4	**Last Modified By:**	wujialing
**Acq. method:**	Related substance method-11-S08.amx	**Injection Date:**	2021-09-27 20:56:51+08:00
**Acq.Method Version:**	2021-0720-0726-37551	**Modified Date:**	2021-09-28 08:27:27+08:00
**Pro.Method:**	*Related Substance.pmx	**Printied Date:**	2021-09-28 08:30:43+08:00
**Pro.Method Version:**	2021-0928-0027-14507	**Result Set Version:**	2021-0928-0027-48807

Work station:: Agilent OpenLAB CDS Software Version:2.3(Build 2.3.0.468)

Result Path: /Desvenlafaxine Succinate/Technology Research/Results/Related Substance/Desvenlafaxine Succinate/20210927-Desvenlafaxine Succinate purity detection (synthesis process optimization).rslt

Data file: Desvenlafaxine Succinate (O-WLFX-20210927-Ph-2).dx

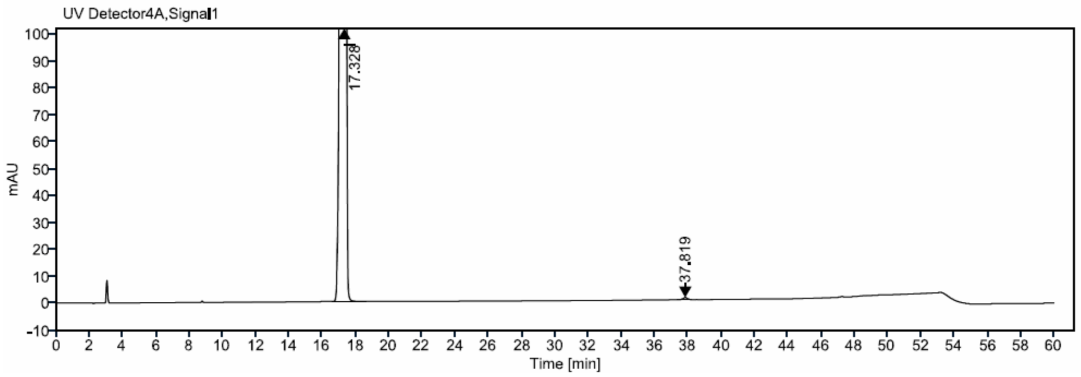

Signal: UV Detector4A,Signal1 Wavelength Ch1 225 nm

Peak Number	RT [min]	Area	Area%	Height	Theoretical Plates	Tail	Resolution
1	17.328	13634.60	99.90	812.90	22681	0.73	
2	37.819	14.05	0.10	0.83	112393	1.04	44.96
	Sum	13648.64	100.00	813.73			

SI-94. HPLC spectrum of DVS-18

琥珀酸去甲文拉法辛分析报告

**Instrument:**	ShimadzuLC08	**Project Name:**	Technology Research
**Manual Modified:**	None	**Operator:**	wujialing
**Sample name:**	Desvenlafaxine Succinate (O-WLFX-20210927-Ph-3)	**Inj. volume:**	10
**Location:**	1:5	**Last Modified By:**	wujialing
**Acq. method:**	Related substance method-11-S08.amx	**Injection Date:**	2021-09-27 21:57:25+08:00
**Acq.Method Version:**	2021-0720-0726-37551	**Modified Date:**	2021-09-28 08:27:27+08:00
**Pro.Method:**	*Related Substance.pmx	**Printied Date:**	2021-09-28 08:31:03+08:00
**Pro.Method Version:**	2021-0928-0027-14507	**Result Set Version:**	2021-0928-0027-48807

Work station:: Agilent OpenLAB CDS Software Version:2.3(Build 2.3.0.468)

Result Path: /Desvenlafaxine Succinate/Technology Research/Results/Related Substance/Desvenlafaxine Succinate/20210927-Desvenlafaxine Succinate purity detection (synthesis process optimization).rslt

Data file: Desvenlafaxine Succinate (O-WLFX-20210927-Ph-3).dx

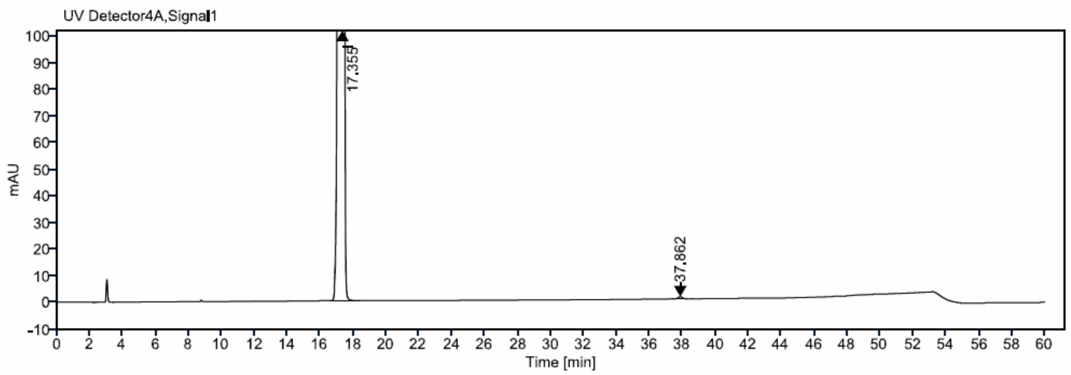

Signal: UV Detector4A,Signal1 Wavelength Ch1 225 nm

Peak Number	RT [min]	Area	Area%	Height	Theoretical Plates	Tail	Resolution
1	17.355	13826.16	99.90	821.22	22605	0.73	
2	37.862	14.04	0.10	0.82	112793	1.04	44.94
	Sum	13840.21	100.00	822.05			
